# Genome Mining Enabled by Biosynthetic Characterization Uncovers a Class of Benzoxazolinate‐Containing Natural Products in Diverse Bacteria

**DOI:** 10.1002/anie.202206106

**Published:** 2022-11-17

**Authors:** Yi‐Ming Shi, Jan J. Crames, Laura Czech, Kenan A. J. Bozhüyük, Yan‐Ni Shi, Merle Hirschmann, Stefanie Lamberth, Peter Claus, Nicole Paczia, Christian Rückert, Jörn Kalinowski, Gert Bange, Helge B. Bode

**Affiliations:** ^1^ Department of Natural Products in Organismic Interactions Max Planck Institute for Terrestrial Microbiology 35043 Marburg Germany; ^2^ Molecular Biotechnology Department of Biosciences Goethe University Frankfurt 60438 Frankfurt am Main Germany; ^3^ Center for Synthetic Microbiology (SYNMIKRO) & Faculty of Chemistry Philipps University of Marburg 35043 Marburg Germany; ^4^ Core Facility for Metabolomics and Small Molecule Mass Spectrometry Max Planck Institute for Terrestrial Microbiology 35043 Marburg Germany; ^5^ Microbial Genomics and Biotechnology Center for Biotechnology (CeBiTec) Bielefeld University 33615 Bielefeld Germany; ^6^ Senckenberg Gesellschaft für Naturforschung 60325 Frankfurt am Main Germany; ^7^ Chemical Biology Department of Chemistry Philipps University of Marburg 35043 Marburg Germany

**Keywords:** Biosynthesis, Heterocycles, Natural Products, Non-Proteinogenic Amino Acids, Non-Ribosomal Peptide Synthetase

## Abstract

Benzoxazolinate is a rare bis‐heterocyclic moiety that interacts with proteins and DNA and confers extraordinary bioactivities on natural products, such as C‐1027. However, the biosynthetic gene responsible for the key cyclization step of benzoxazolinate remains unclear. Herein, we show a putative acyl AMP‐ligase responsible for the last cyclization step. We used the enzyme as a probe for genome mining and discovered that the orphan benzobactin gene cluster in entomopathogenic bacteria prevails across Proteobacteria and Firmicutes. It turns out that *Pseudomonas chlororaphis* produces various benzobactins, whose biosynthesis is highlighted by a synergistic effect of two unclustered genes encoding enzymes on boosting benzobactin production; the formation of non‐proteinogenic 2‐hydroxymethylserine by a serine hydroxymethyltransferase; and the types I and II NRPS architecture for structural diversity. Our findings reveal the biosynthetic potential of a widespread benzobactin gene cluster.

## Graphical abstract

Benzoxazolinate is a rare bis‐heterocyclic moiety that interacts with proteins and DNA. It was found that a putative acyl AMP‐ligase mediates the last cyclization step to afford benzoxazolinate. The enzyme was then used as a probe for genome mining, which led to the discovery of a new class of benzoxazolinate‐containing compounds in diverse bacteria.

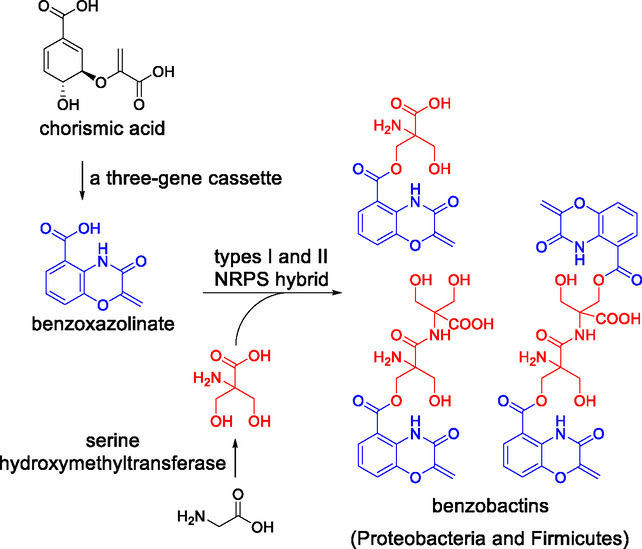

## Introduction

**Figure 1:**
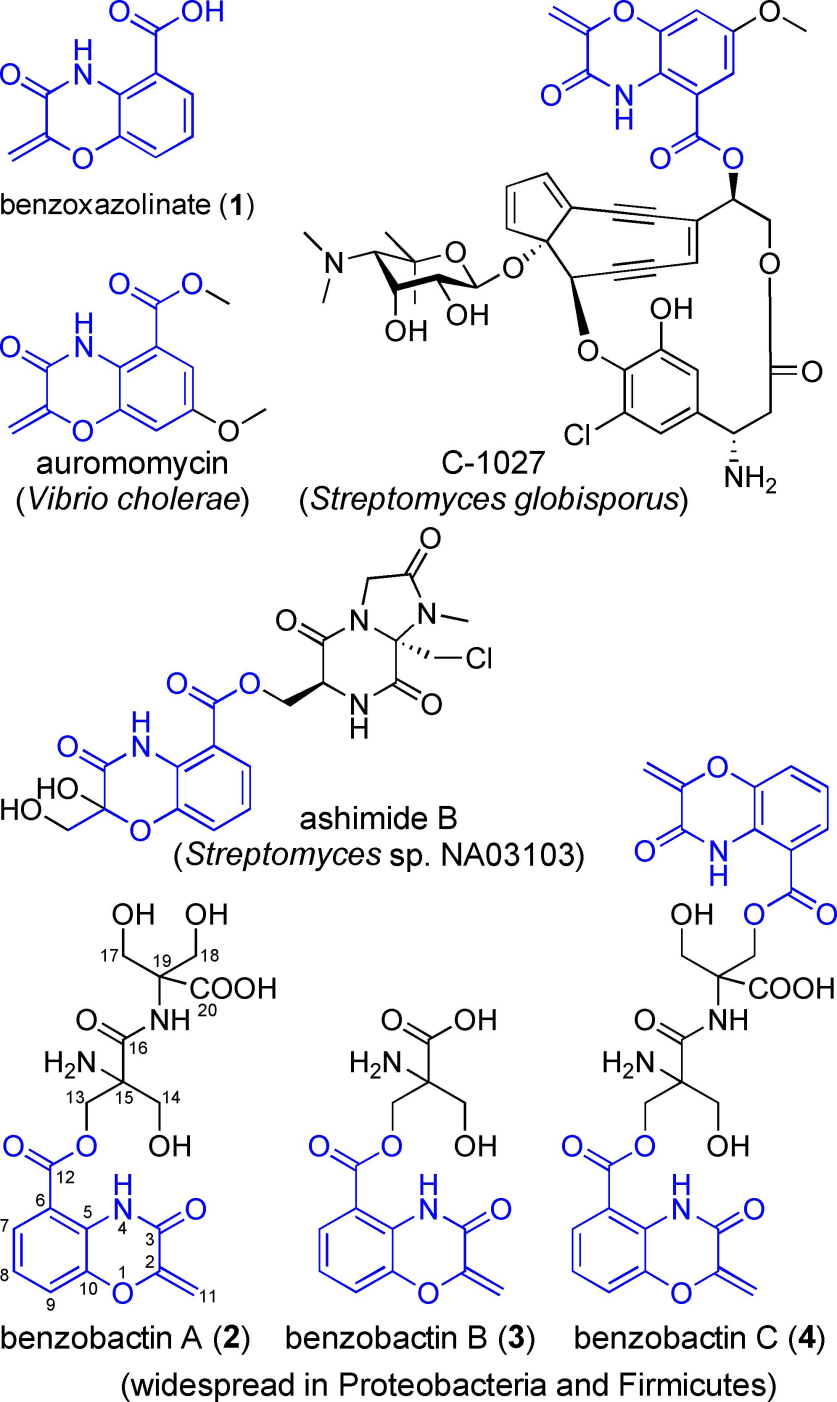
Representative natural products containing a benzoxazolinate moiety reported thus far, as well as previously unknown benzobactins B (**3**) and C (**4**).

**Figure 2:**
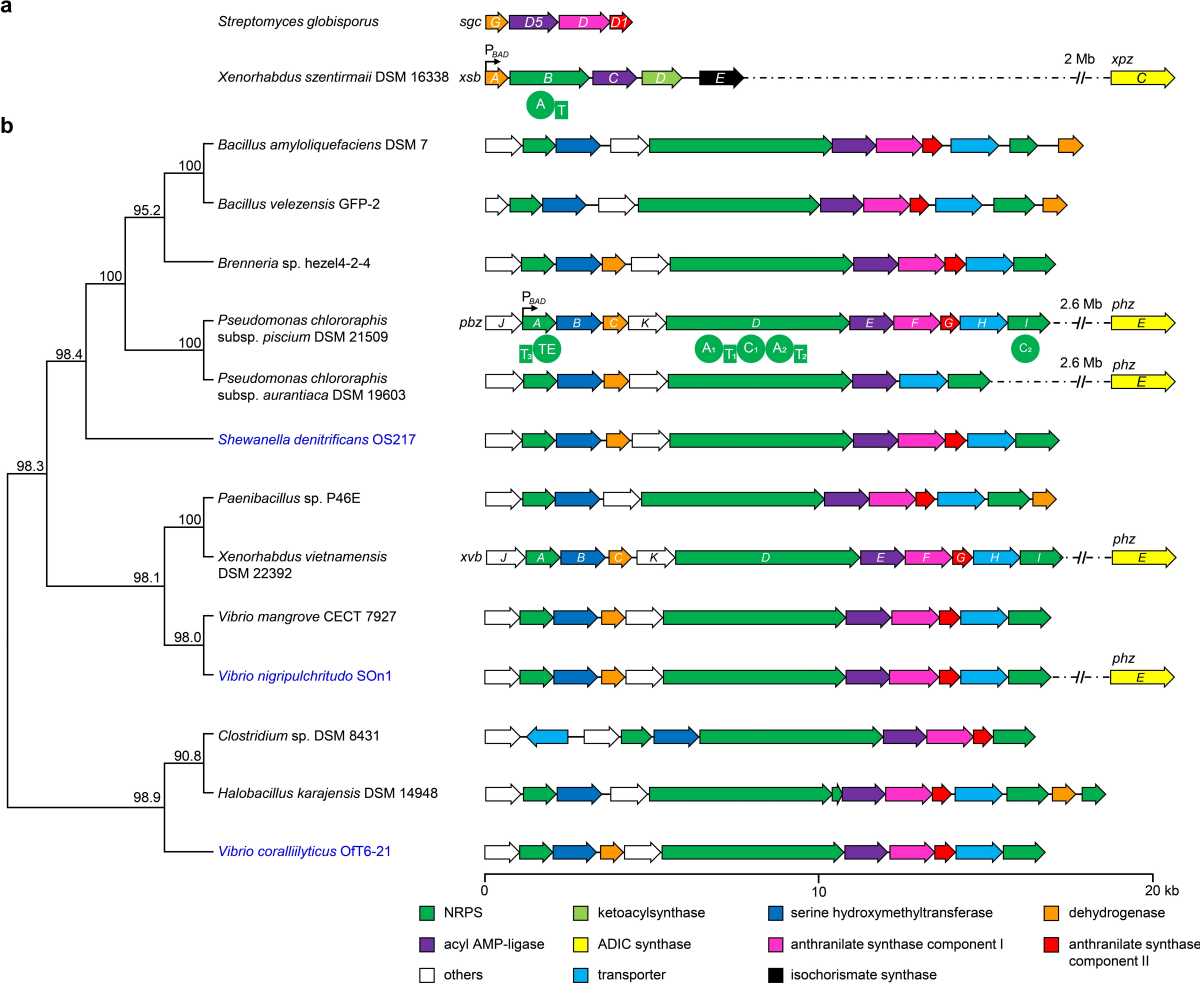
BGCs encoding the biosynthesis of benzoxazolinate and benzobactins in different strains. a) BGCs encoding the biosynthesis of **1** from *S. globisporus* (*sgc*) and *X. szentirmaii* DSM 16338 (*xsb*). b) Phylogenetic tree and gene organization of the *pbz* and *xvb* BGCs and their representative homologs. The tree is based on protein sequences of putative benzobactin BGCs. Numbers next to the branch indicate the percentage of replicate trees in which this topology was reached using a bootstrap test of 1000 replicates. Microbes that originate from marine resources are highlighted in blue. ADIC synthases are encoded by *xpzC* and *phzE*, which are unclustered with the benzoxazolinate and benzobactin BGCs, and their distances from the BGC in the genome are indicated if available. A black arrow in the BGC shows the position where an arabinose‐inducible promoter P_*BAD*_ is inserted. A, adenylation; T, thiolation; C, condensation; and TE, thioester domain. kb, kilobase.

**Figure 3:**
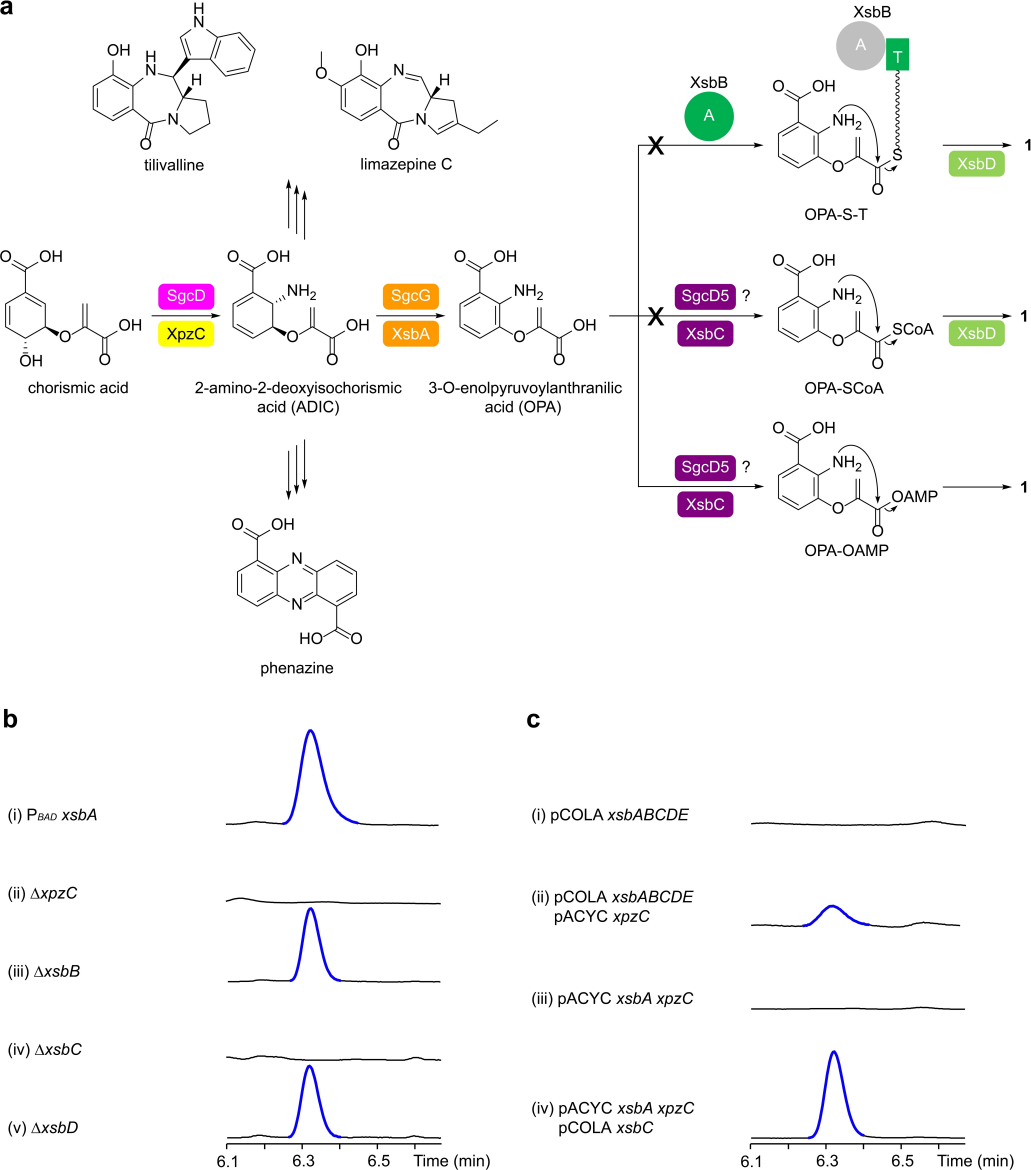
Characterization of the benzoxazolinate biosynthesis in *X. szentirmaii* DSM 16338. a) Biosynthetic pathway of **1** in *X. szentirmaii* (*xsb*) and *S. globisporus* (*sgc*). ADIC serves as a key branching point for the biosynthesis of tilivalline, limazepine, and phenazine. The color codes enzymes are corresponding to the encoded genes in Figure 2. A, adenylation; and T, thiolation domains. b) HPLC‐UV analysis of (i) the constructed promoter exchange mutant *X. szentirmaii* P_*BAD*_ *xsbA* and (ii‐v) deletion mutants. Deletions were carried out in the *X. szentirmaii* P_*BAD*_ *xsbA* mutant. Mutants expressing *xsb* BGC were induced by l‐arabinose. c) HPLC‐UV analysis of (i‐iv) heterologous expression of *xsb* and *xpz* genes in *E. coli* BL21(DE3) induced by IPTG. **1** (blue) is highlighted in traces.

Benzoxazolinate (**1**) moiety is a rare feature in natural products and was first found in C‐1027 (Figure 1),^[1]^ endowing C‐1027 with remarkable biological activities. The bis‐heterocyclic moiety not only stabilizes the enediyne chromophore of C‐1027 during biosynthesis and secretion by noncovalently binding to the CagA apoprotein,[^2^, ^3^] but also intercalates DNA and positions the enediyne core in the minor groove^[4]^ to induce DNA cleavage.^[5]^ The biosynthetic genes responsible for synthesizing **1** were first identified in *Streptomyces globisporus*.^[6]^ Subsequent in vitro functional characterization^[7]^ of two key enzymes encoded by the *sgc* biosynthetic gene cluster (BGC; Figure 2a) revealed that SgcD, an anthranilate synthase component I homolog, unexpectedly converts chorismic acid into 2‐amino‐2‐deoxyisochorismic acid (ADIC; Figure 3a), which is devoid of cleaving pyruvate activity as usually observed for anthranilate synthase component I. Then, an FMN‐dependent and [Fe−S]‐containing enzyme, SgcG, dehydrogenates the dihydroaromatic ring of ADIC to an aromatic scaffold without loss of the enolpyruvate, leading to 3‐*O*‐enolpyruvoylanthranilic acid (OPA) formation. However, the enzyme catalyzing intramolecular attack of the NH_2_ on the carboxylate of the enolpyruvate to afford a 1,4‐oxazolinate ring remains undefined.

Auromomycin^[8]^ and ashimides^[9]^ (Figure 1) are two additional classes of benzoxazolinate‐containing natural products, of which auromomycin is a nonbacteridical, potent inhibitor against biofilm formation in *Vibrio*.^[8]^ Cytotoxic benzobactin A (**2**) is the product of the *xvb* pathway in the entomopathogenic bacterium *Xenorhabdus vietnamensis*,^[10]^ featuring two non‐proteinogenic amino acid residues, 2‐hydroxymethylserines, that are a rare building block only found in antrimycin^[11]^ thus far. The *xvb* BGC coding for types I and II non‐ribosomal peptide synthetase (NRPS) hybrid^[12]^ was identified by extensive mapping of *Xenorhabdus* and *Photorhabdus* BGCs to the MIBiG database,^[10]^ and the biosynthetic machinery of **2** is yet to be characterized. To date, benzoxazolinate‐containing natural products were only accessible with these four bacterial producers. Here, we present that genome mining enabled by gene functional characterization of benzoxazolinate biosynthesis provides a prolific producer for a class of benzoxazolinate derivatives, whose BGC is widely distributed across Gram‐negative and Gram‐positive strains.

## Results and Discussion

### Biosynthetic Crosstalk

To explore potential alternative benzoxazolinate‐producing hosts among *Xenorhabdus* and *Photorhabdus*, we used the protein sequence of SgcG as a probe to search for putative BGCs. We found that in addition to XvbC encoded by the known *xvb* BGC in *X. vietnamensis*,^[10]^ another two SgcG homologs present in the genomes of *X. szentirmaii* DSM 16338 and *X. szentirmaii* US are located in two highly similar gene clusters, of which the BGC in *X. szentirmaii* DSM 16338 is designated as *xsb*. The *xsb* BGC (Figure 2a and Table S4) is strikingly different from the part of *sgc* BGC encoding benzoxazolinate biosynthesis (hereafter “*sgc* BGC” refers to the benzoxazolinate cluster in *S. globisporus*). The *xsb* BGC lacks an SgcD homolog, and only *xsbA* (encoding a dehydrogenase) and *xsbC* (encoding a phenylacetate‐CoA ligase family enzyme) contain a counterpart in the *sgc* BGC. The remaining *xsb* BGC is made up of *xsbB* which encodes an NRPS with an adenylation (A) and a thiolation (T) domain, *xsbD* that encodes a 3‐oxoacyl‐ACP synthase, and *xsbE* that encodes an isochorismate synthase.

To explore the biosynthetic theme of *xsb* BGC, we inserted an arabinose‐inducible P_*BAD*_ promoter^[13]^ in front of *xsbA* and found that the induced P_*BAD*_ *xsbA* mutant yielded a single peak (Figure 3b, trace i) via untargeted metabolite searches. The compound was isolated and determined to be **1** by HRMS (Table S5) and NMR spectroscopy (Table S6 and Figure S1). However, in contrast to the homologous expression of *xsb* BGC, the heterologous expression of *xsbABCDE* in *Escherichia coli* BL21(DE3) did not produce **1** (Figure 3c, trace i). These results suggest that the *xsb* BGC alone is insufficient to fulfill production of **1** and that most likely, an enzyme encoded by a discrete gene for ADIC synthesis is required. Given that ADIC is a common precursor of phenazine^[14]^ and pyrrolobenzodiazepine^[15]^ (e.g. tilivalline and limazepines) (Figure 3a), we postulated that the deficiency of *xsb* BGC in synthesizing ADIC precursor is complemented by the phenazine or pyrrolobenzodiazepine pathway. The genome of *X. szentirmaii* does not encode pyrrolobenzodiazepine‐related pathways, and therefore it is highly likely that the *xsb* BGC “borrows” ADIC derived from the phenazine pathway (*xpz*), which has a high expression level in the wild‐type strain.^[16]^ Consistent with our hypothesis, co‐expression of *xsbABCDE* with the ADIC synthase encoding gene *xpzC* (homologous to PhzE^[17]^) from a typical phenazine operon^[16]^ yielded **1** (Figure 3c, trace ii). Interestingly, XpzC which is the only ADIC synthase present in *X. szentirmaii* DSM 16338 is functionally identical to SgcD, but both share a low sequence identity (14.5 %), consistent with previously described.^[7]^ Two putative anthranilate synthase component I encoding genes, *xsze*_*03053* and *xsze*_*03255*, were found when using SgcD as a probe to search for homologs in the genome of *X. szentirmaii* DSM 16338. We were concerned that Xsze_03053 and Xsze_03255 could also synthesize ADIC and such genetic redundancy within *X. szentirmaii* DSM 16338 could functionally complement XpzC. Therefore, we deleted *xpzC* and the obtained P_*BAD*_ *xsbA ΔxpzC* mutant lost production of **1** (Figure 3b, trace ii), which points to the essential role of *xpzC* from a phenazine operon in generating the ADIC precursor for the *xsb* pathway (Figure 3a).

### Cyclization of OPA Intermediate

We next explored the cyclization step of OPA to give **1** in the *xsb* BGC. Upon closer examination of the gene component, we notice that *xsbB* features an A domain that might adenylate non‐amino acid substrates, due to the lack of a conserved aspartate residue in motif A4 that typically interacts with the α‐NH_2_ of amino acid substrates^[18]^ (Figure S2). Also, a 3‐oxoacyl‐ACP synthase encoded by *xsbD* has a high sequence identity (83.6 %) with XpzS^[16]^ that catalyzes amide‐ and ester‐bond formation between the carrier‐protein‐bound aminoacyl with phenazines. Therefore, there are three conceivable pathways to achieve OPA cyclization (Figure 3a): 1) the A domain of XsbB adenylates OPA for loading onto the T domain, and subsequently, XsbD catalyzes intramolecular amide‐bond formation; 2) XsbC thioesterifies the carboxylate of enolpyruvate with a CoASH, followed by amide‐bond formation under XsbD catalysis; or 3) the cyclization occurs in a non‐thiotemplated fashion, during which XsbC activates the carboxylate of enolpyruvate through ATP‐dependent adenylation, followed by direct nucleophilic attack of the NH_2_ on the carboxylate without forming a CoA‐bound thioester intermediate, as observed during the biosyntheses of coumermycin A1,^[19]^ dapdiamide,^[20]^ and yatakemycin.^[21]^ To confirm the involvement of genes in OPA cyclization, we individually deleted *xsbB*, *xsbC*, and *xsbD*. While the P_*BAD*_ *xsbA ΔxsbB* and P_*BAD*_ *xsbA ΔxsbD* mutants did not affect the product profile (Figure 3b, traces iii and v), **1** was completely absent in the P_*BAD*_ *xsbA ΔxsbC* mutant (Figure 3b, trace iv), revealing *xsbC* being solely responsible for the formation of **1** from OPA. We then verified the function of XsbC by in vivo experiments. *E. coli* BL21(DE3) harboring *xsbA*, *xpzC*, and *xsbC* produced **1**, while no production of **1** was observed in *E. coli* expressing *xsbA* and *xpzC* (Figure 3c, traces iii and iv).

To support our hypothesis that XsbC belonging to the phenylacetate‐CoA ligase family mediates OPA cyclization via an ATP‐dependent adenylating manner (Figure 3a), we pursued a maximum‐likelihood‐based phylogenetic analysis^[22]^ to infer the relationships between XsbC and enzymes in the ANL (acyl‐CoA synthetases, NRPS adenylation domains, and luciferase enzymes) superfamily. The resultant tree (Figure S3) showed that XsbC is distantly related to NatL2 and BomJ, both of which are acyl‐AMP ligases involved in intermolecular ester‐bond formation.[^23^, ^24^] Although a multi‐sequence alignment indicated a low sequence identity (19.8 %) of XsbC with NatL2, a closer inspection of the alignment (Figure S4) and homology modeling (Figures S5–8) revealed that XsbC harbors diagnostic binding sites and conserved motifs that are observed in NatL2.^[23]^ The Zn^2+^‐binding tetrad Cys263‐His269‐Cys321‐Cys323 is a unique feature in distinguishing NatL2 from canonical Mg^2+^‐binding CoA ligases and is well conserved in XsbC (Figures S4 and S6). Most importantly, a C‐terminal extension with Lys429 in NatL2 is also present in XsbC. Lys429 from the other monomer forms a salt bridge with the AMP (Figure S7), and thus C‐terminal extension crossing to the other monomer is proposed to lock the functional homodimer in the “close” conformation, which prevents a CoA from entering the binding pocket.

Taking all these data into account, we propose a three‐gene cassette consisting of an anthranilate synthase component I homolog (e.g. SgcD) or ADIC synthase (e.g. XpzC), an FMN‐dependent and [Fe−S]‐containing dehydrogenase (e.g. SgcG and XsbA), and an acyl‐AMP ligase (e.g. XsbC) being responsible for benzoxazolinate biosynthesis.

### Benzobactin BGC in Diverse Bacteria

To expand the biosynthetic repertoire of benzoxazolinate‐containing natural products, we carried out a survey of all bacterial genomes available in the NCBI database using the protein sequence of XsbC as a query. Besides C‐1027^[6]^ and ashimides^[9]^ homologous BGCs found in Actinobacteria as we expected, the Blast result revealed that the orphan *xvb* BGC responsible for benzobactin biosynthesis in entomopathogenic bacteria *Xenorhabdus* and *Photorhabdus* ^[10]^ abounds in *Pseudomonas*, and is prevalent across two phyla of bacteria (Figure 2b), the Proteobacteria (e.g. *Pseudomonas*, *Vibrio*, *Shewanella*, and *Brenneria*) and Firmicutes (e.g. *Bacillus*, *Paenibacillus*, *Halobacillus*, and *Clostridium*). Such extensive similarities to the *xvb* BGC suggest that benzoxazolinate‐containing natural products are more widespread in diverse bacteria—from ocean to land—than we thought, and that *Pseudomonas* being the most abundant genus among all these strains might be prolific producers for these rare natural products.

**Figure 4:**
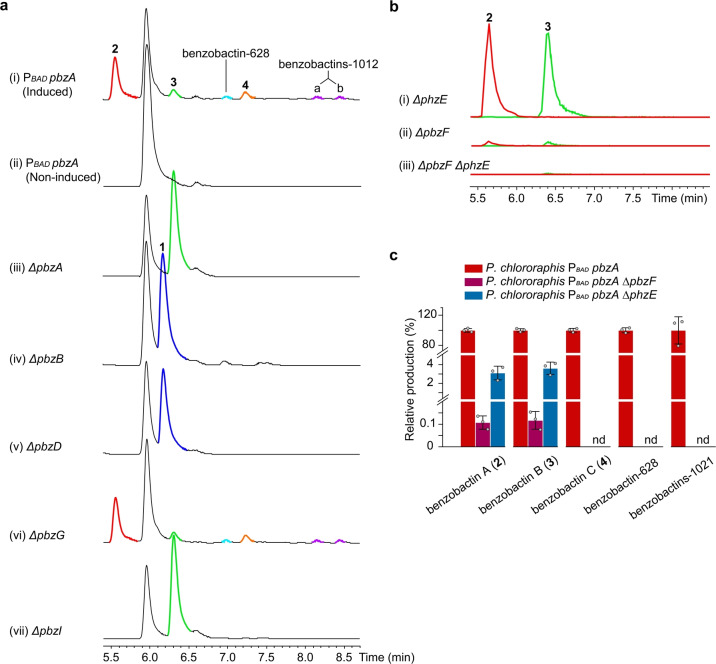
HPLC analysis of *P. chlororaphis* subsp. *piscium* DSM 21509 mutants. a) HPLC‐UV analysis of (i) the constructed promoter exchange mutant *P. chlororaphis* P_*BAD*_ *pbzA* and (iii–vii) deletion mutants. Deletions were carried out in the P_*BAD*_ *pbzA* mutant. Mutants expressing the *pbz* BGC were induced by l‐arabinose. **1** (dark blue) and **2** (red), **3** (green), and **4** (orange), as well as the as‐yet‐uncharacterized benzobactins‐628 (light blue) and 1021 (purple), are highlighted in the traces. (i) The P_*BAD*_ *pbzA* and (vi) P_*BAD*_ *pbzA ΔpbzG* mutants shared an identical metabolite profile, indicating that PbzG (homologous to anthranilate synthase component II) might not be involved in benzobactin biosyntheses, or that its function could be complemented by other genes in the genome. b) HPLC‐MS analysis of (i–iii) the *ΔpbzF*, *ΔphzE*, and *ΔpbzF ΔphzE* mutants for benzobactin production. Deletions were carried out in the *P. chlororaphis* P_*BAD*_ *pbzA* mutant. Shown are the EICs of **2** (red) and **3** (green) in deletion mutants. c) Production of benzobactins in the *ΔpbzF* and *ΔphzE* mutants. The production of individual compounds at each deletion mutant is relative to its production in the P_*BAD*_ *pbzA* mutant (in percentage, %). nd=not detected.

We selected different *Pseudomonas* strains for homologously expressing the target BGC by a promoter‐exchange strategy.^[25]^ A BGC, designated as *pbz*, from *Pseudomonas chlororaphis* subsp. *piscium* DSM 21509 was successfully activated as indicated by the significant distinction of HPLC profiles between induced and non‐induced mutants (Figure 4a, traces i and ii). In addition to **2** produced in a high amount, the *P. chlororaphis* P_*BAD*_ *pbzA* mutant yielded five more benzobactin derivatives with masses ranging from 305 to 1012 (Table S5) as determined by untargeted analysis, and therefore we attempted to isolate them for structure elucidation. The compound with a mass of 305, designated as benzobactin B (**3**) confirmed by NMR spectroscopy (Table S6 and Figure S1), has one 2‐hydroxymethylserine residue less than **2**. Compounds with larger masses (e.g. 609, 628, and 1012) were not isolated due to their low production levels and instability. Among them, the structure of a compound with a mass of 609, designated as benzobactin C (**4**), was tentatively assigned to be a dimer of **3** by tandem MS/MS and isotope labeling experiments (Figures S9 and S10a). Based on the sum formula (Table S5), we proposed that benzobactins‐628 and 1012 are a dimer and tetramer of **3**, respectively, while their exact chemical structures cannot be formulated at the current stage.

### Synergistic Effect of ADIC and Anthranilate Synthases

The *pbz* BGC contains a typical three‐gene benzoxazolinate cassette *pbzCEF*. An ADIC synthase (PhzE) in a phenazine biosynthetic operon that is 2.6 megabase pairs away from the *pbz* BGC is also present in the genome (Figure 2b). Again, consistent with the speculation that the ADIC precursor could be synthesized by PhzE and PbzF, both of which are functionally complementary, the P_*BAD*_ *pbzA ΔphzE* and P_*BAD*_ *pbzA ΔpbzF* mutants still yielded **2** and **3** (Figure 4b, traces i and ii), while the products completely lost in the P_*BAD*_ *pbzA ΔpbzF ΔphzE* mutant (Figure 4b, trace iii). Surprisingly, the production titers of **2** and **3** decreased ≈30‐fold in the P_*BAD*_ *pbzA ΔphzE* mutant and ≈900‐fold in the P_*BAD*_ *pbzA ΔpbzF* mutant, compared to that of the P_*BAD*_ *pbzA* mutant (Figure 4c). This suggests that PbzF and PhzE are not only functionally complementary but also have a synergistic positive effect on production level of the downstream benzobactin products, presumably because the ADIC metabolic flux is dramatically increased by both enzymes. The coexistence of *pbzF* and *phzE* also occurs in the genomes of *X. vietnamensis* DSM 22392 and *Vibrio nigripulchritudo* SOn1 (Figure 2b). These observations perhaps are unsurprising, because phenazines, which often have a high expression level being the pigmentation phenotype of wild‐type strains, are much more widely distributed in bacteria (particularly in *Pseudomonas*)[^26^, ^27^] than benzoxazolinate derivatives.

### Unusual Serine Hydroxymethyltransferase

**Figure 5:**
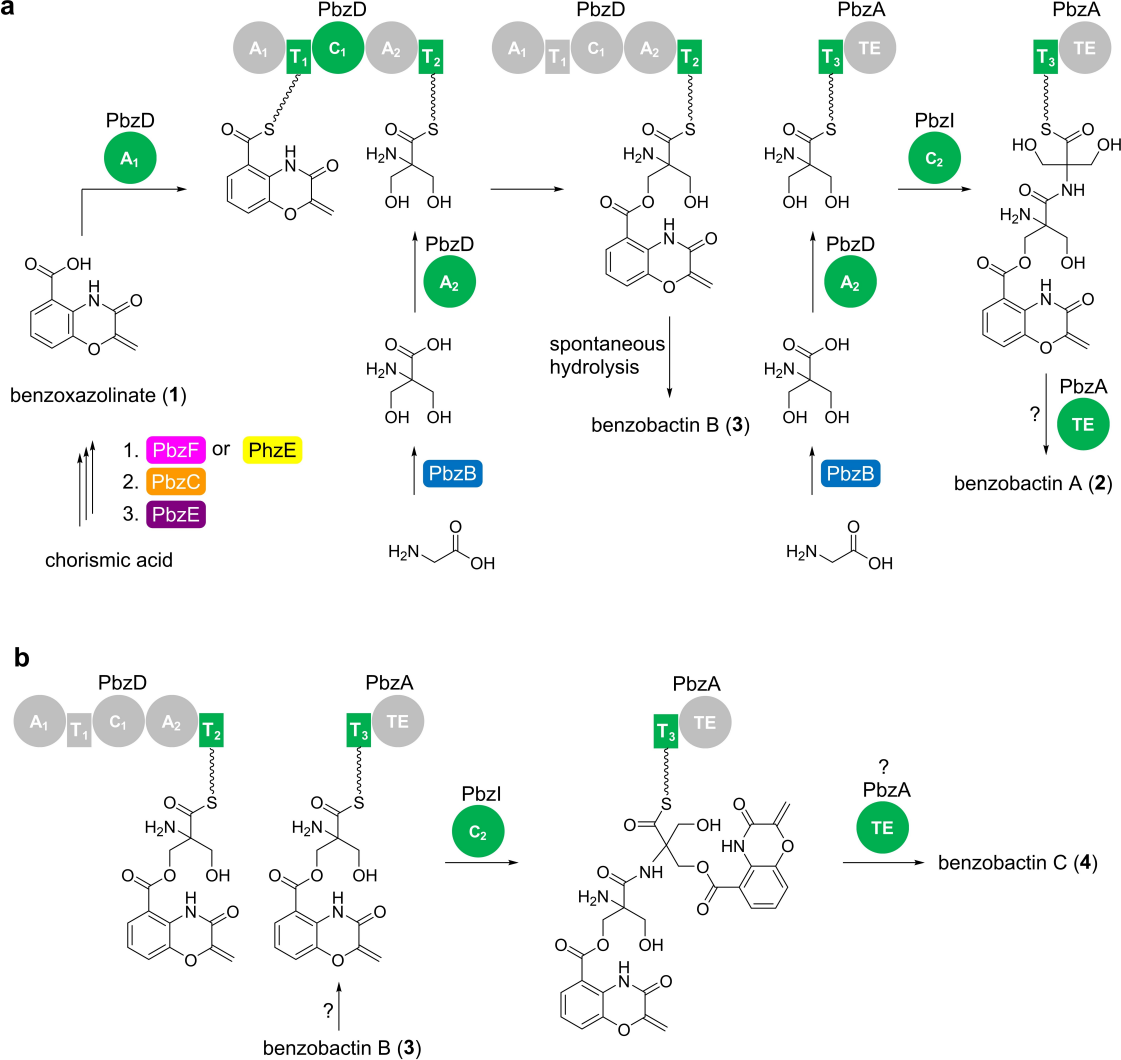
Proposed biosynthesis of benzobactins *in P. chlororaphis* subsp. *piscium* DSM 21509. a) Proposed biosynthetic pathways of **2** and **3**. b) Proposed biosynthetic pathway of **4**. Domain involved in the corresponding reaction step is highlighted in green. The color codes enzymes are corresponding to the encoded genes in Figure 2. A, adenylation; T, thiolation; C, condensation; and TE, thioester domains.

The bimodular NRPS, PbzD, appears to be responsible for loading and elongating **1** (Figure 5a), since the A_1_ domain is non‐amino‐acid substrate specific, as indicated by its motif A_4_ (Figure S2). This is supported by the P_*BAD*_ *pbzA ΔpbzD* mutant that abolished production of benzobactins but accumulated that of **1** (Figure 4a, trace v). It is proposed that the incorporation of a 2‐hydroxymethylserine into the benzoxazolinate moiety is performed by the second module of PbzD with the assistance of PbzB which is a putative serine hydroxymethyltransferase. Pyridoxal‐5′‐phosphate‐dependent (PLP) serine hydroxymethyltransferases were reported to catalyze the transfer of a hydroxymethyl group from 5,10‐methylenetetrahydrofolate (mTHF) to glycine or alanine to afford l‐serine or α‐methylsereine, as found during the biosyntheses of ashimides^[9]^ and JBIR‐34/35 (ref. [28]). PbzB has high sequence similarity with AsmD^[9]^ and FmoH,^[28]^ and contains a conserved PLP‐binding loop (Figure S11) in which the lysine residue forms a Schiff base with PLP.^[29]^ Therefore, we assumed that PbzB could catalyze mono‐hydroxymethylation of serine or/and bis‐hydroxymethylation of glycine to afford 2‐hydroxymethylserine.

To verify the origin of building blocks in vivo, we cultivated the P_*BAD*_ *pbzA* mutant in ^15^N media supplemented with l‐serine and glycine. Exemplified by compound **3**, while no inverse mass shifts were obtained in the ^15^N medium supplemented with l‐serine, we observed—1 Da mass shift in the ^15^N medium supplemented with glycine, which resulted from the incorporation of a glycine residue (Figure S10b). Phylogenetic analysis revealed that the PbzD‐A_2_ domain unexpectedly falls into the clade of A domains with cysteine specificity and is separate from those with glycine or serine specificity (Figure S12). This suggests that PbzD‐A_2_ domain might be evolved for larger substrates and adenylate non‐glycine substrates for loading onto PbzD‐T_2_ domain. Thus, we propose that PbzB catalyzes the conversion of glycine into 2‐hydroxymethylserine prior to being subjected to the PbzD‐A_2_ domain activation (Figure 5a).

**Figure 6:**
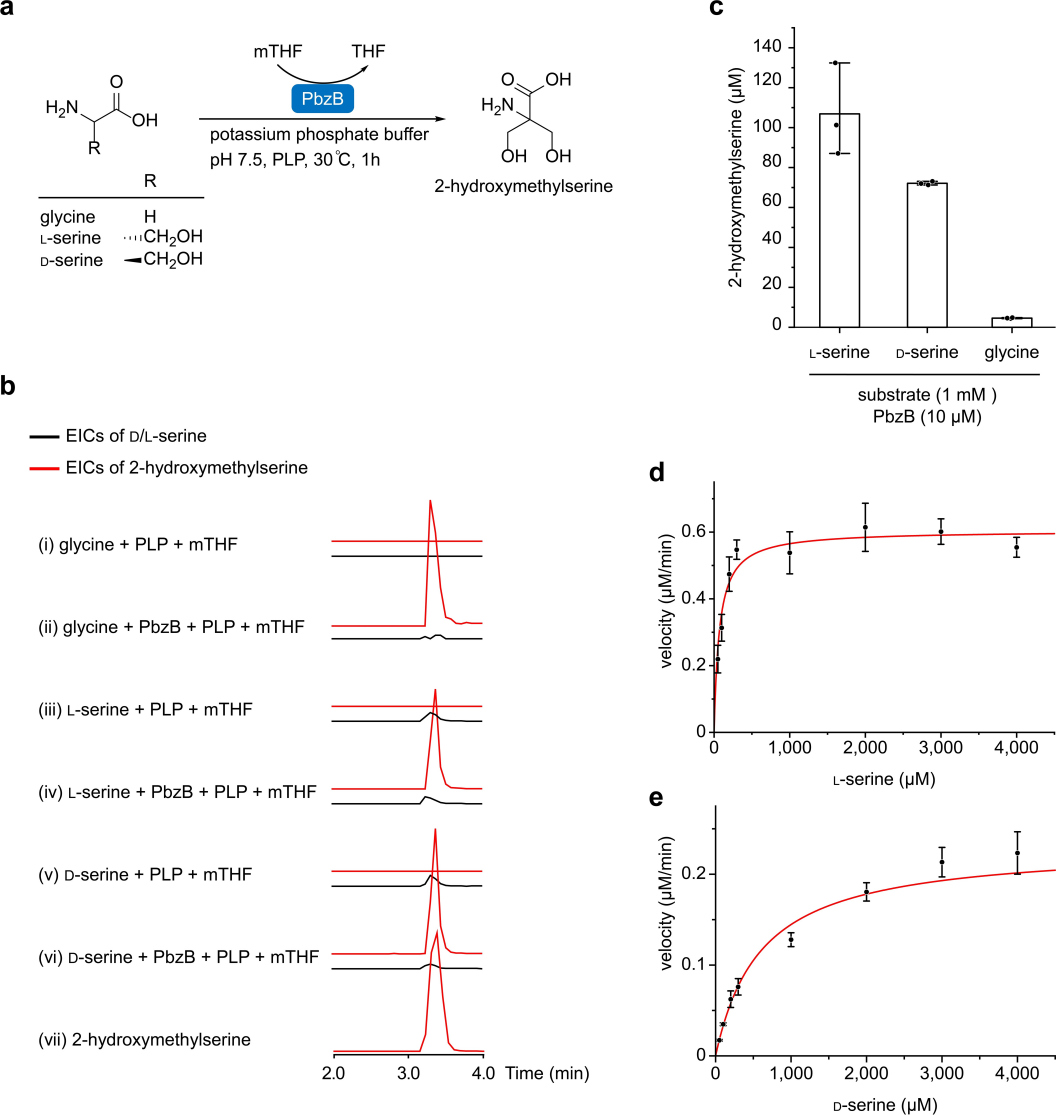
In vitro characterization of 2‐hydroxymethylserine conversions. a) Enzymatic conversion of glycine and d‐/l‐serine to 2‐hydroxymethylserine using the recombinant serine hydroxymethyltransferase PbzB. b) EICs of d‐/l‐serine (black, 106.0492 [*M*+H]^+^) and 2‐hydroxymethylserine (red, 136.0604 [*M*+H]^+^) are shown, while the mass of glycine is too small to be detected. c) Quantification of 2‐hydroxymethylserine production with d‐/l‐serine and glycine substrates. Steady‐state kinetics of formation of 2‐hydroxymethylserine from d) l‐serine (*K*_m_=69.52±0.03 μM, *k*_cat_=0.151±0.006 min^−1^) and e) d‐serine (*K*_m_=602.90±56.67 μM, *k*_cat_=0.058±0.003 min^−1^).

To verify the conversion of glycine into 2‐hydroxymethylserine by PbzB, we expressed and purified PbzB with a His‐SUMO‐tag from *E. coli* BL21(DE3) (Figure S13a) and conducted in vitro enzymatic assays (Figure 6a). Incubation of PbzB with glycine and cofactors, PLP and mTHF, in the potassium phosphate buffer at pH 7.5 showed the formation of 2‐hydroxymethylserine and a trace amount of serine (Figure 6b, trace ii). This finding motivated us to examine whether PbzB can also catalyze the conversion of d‐/l‐serine into 2‐hydroxymethylserine. Surprisingly, under the same enzymatic reaction condition, the production of 2‐hydroxymethylserine that was converted from d‐/l‐serine was much higher than that from glycine (Figure 6c). While glycine was the authentic building block in vivo, serine was a more favored in vitro substrate for PbzB than glycine. Two possible explanations for the seemingly conflicting results are that 1) in in vitro, the one‐step conversion of serine to 2‐hydroxymethylserine is faster than the two‐step conversion of glycine to 2‐hydroxymethylserine; 2) in in vivo, the degradation of serine to glycine catalyzed by the serine hydroxymethyl transferase from primary metabolism is faster than the formation of serine to 2‐hydroxymethylserine catalyzed by PbzB. We then carried out a steady‐state kinetic analysis for the formation of 2‐hydroxymethylserine using d‐/l‐serine (Figure 6d and e), which suggested that the affinity of PbzB for l‐serine is 9‐fold higher than that for d‐serine as indicated by the *K*_m_ values, and that PbzB is 22‐fold more efficient at catalyzing the formation of 2‐hydroxymethylserine with l‐serine than that with d‐serine based on the *k*_cat_/*K*_m_ values.

### Bioinformatics and Structure of PbzB

Serine hydroxymethyltransferases involved in the central metabolism have been studied in various organisms.^[30]^ While GlyA from *E. coli*, one of the best‐studied examples, catalyzes reversible conversion between serine and glycine and thereby affords mTHF as the major source of C1 unit in cells,^[31]^ the newly identified PbzB involved in the benzobactin biosynthesis catalyzes production of 2‐hydroxymethylserine using l‐serine, d‐serine, or glycine. Sequence alignment analysis (Figure S11b) of PbzB and its homologs AsmD,^[9]^ FmoH,^[28]^ and XvbB^[10]^ (PbzB type) in comparison with structurally and biochemically characterized GlyA‐type enzymes revealed a high sequence similarity of over 42 %. Almost all residues involved in coordinating *N*‐pyridoxyl‐glycine‐5‐phosphate (PLG) and mTHF were conserved among the analyzed proteins (Figure S11b). While GlyA‐type enzymes contain a conserved **Y**AEG— ‐RY**Y** motif, where the two tyrosine residues (Y65 and Y55) are crucial for efficient substrate conversion and the formation of cation π‐interaction with the ligand,[^32^, ^33^] the PbzB‐type enzymes harbor a **T**AEG— ‐RY**H** motif (Figure S11b).

**Figure 7:**
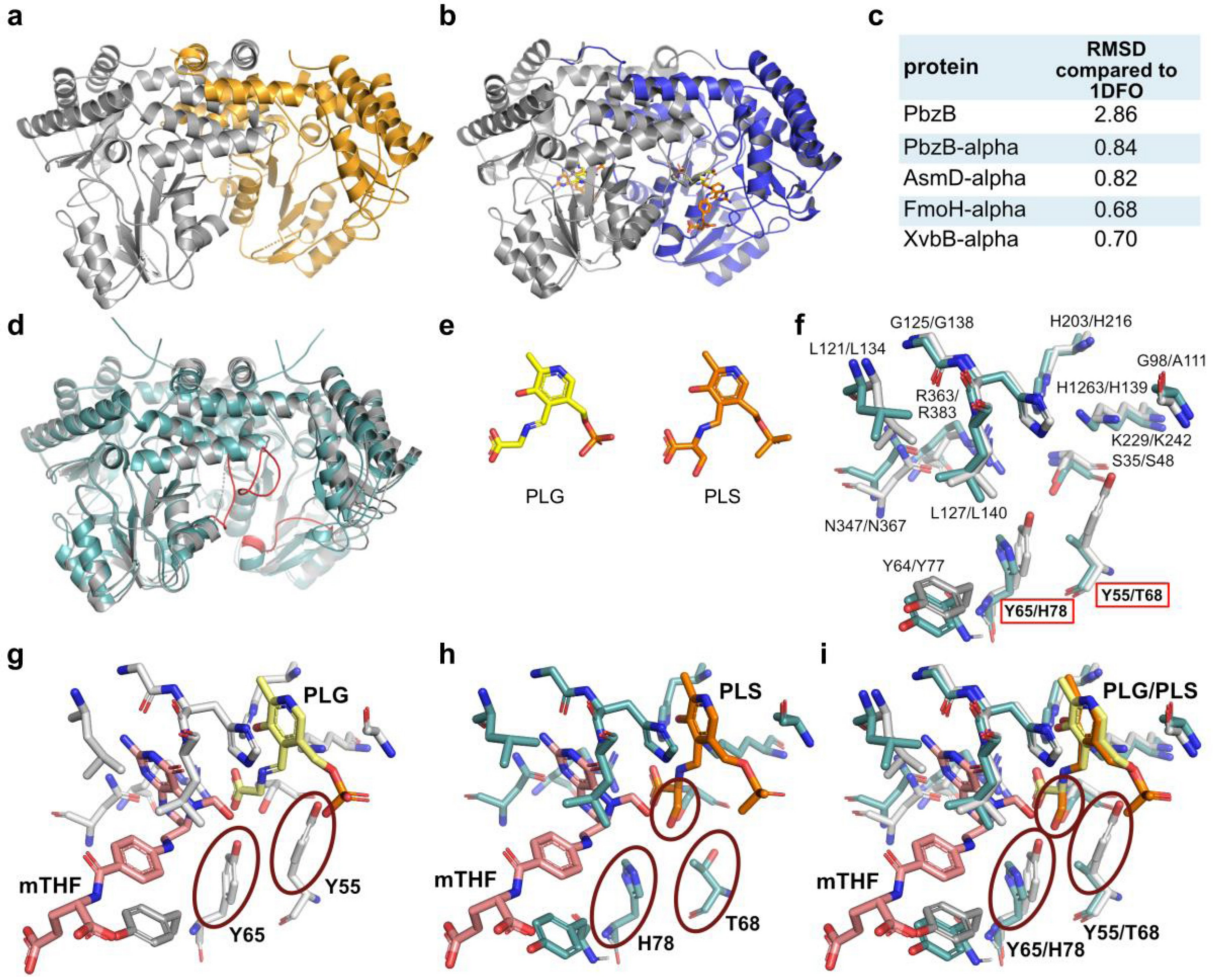
Structural and bioinformatical analysis of PbzB and its homologs. a) Overall‐fold of the crystal structure of apo PbzB. The individual monomers of the dimeric complex are shown in grey and orange (PDB 7QWZ). b) Overall‐fold of the crystal structure of GlyA in complex with PLG (yellow) and mTHF (pink). The two monomers of the GlyA dimer are shown in grey and blue (PDB 1DFO).^[31]^ c) Root mean square deviation (RMSD) of PbzB apo structure (PDB 7QWZ), PbzB model, AsmD model, FmoH model, and XvbB model (models obtained by Alphafold) in comparison to the crystal structure of GlyA (PDB 1DFO).^[31]^ d) Overlay of the PbzB Alphafold model (green) and the PbzB apo crystal structure (grey). Undissolved regions of the PbzB crystal structure are highlighted with red in the PbzB Alphafold model. e) Comparison of the PLP‐bound substrates PLG and PLS. f) Overlay of the residues present in the active site of PbzB (Alphafold model, green) and GlyA (crystal structure, PDB 1DFO, grey). g) Detailed view of the GlyA active site coordinating mTHF (pink) and PLG (yellow). h) Modeled view of the PbzB active site coordinating mTHF (pink) and PLS (orange). i) Overlay of the active sites of GlyA and the PbzB Alphafold model. In (g)–(i), the residues with red circles are critical for fitting the amino acid substrate into the active site. In i, possible stereo hinderance between serine and Y65/Y55 in GlyA is indicated.

To gain an insight into the substituted residues involved in substrate binding in PbzB, we set out to structurally analyze its apo‐ and substrate‐bound form. While crystals of the apo‐form diffracted to 2.8 Å, we only obtained poorly diffracting anisotropic crystals for PbzB co‐crystallized with l‐serine and mTHF. The crystal structure of apo‐PbzB (Figure 7a; Table S7; PDB 7QWZ) shared the overall fold of GlyA‐type enzymes with an RMSD value of 2.86 to GlyA (Figure 7b and c). PbzB, same as GlyA‐type enzymes, forms homodimers with two active sites shared between the two monomers (Figure 7a and b). However, the residues coordinating PLG and mTHF were not visible in the PbzB structure, since they were probably too flexible in the absence of a ligand (Figure 7d), and therefore, we modeled PbzB using Alphafold.^[34]^ The simulated structural model had a confidence of over 95 % and an RMSD of 0.97 and 0.84 for the crystal structures of apo‐PbzB and GlyA from *E. coli*,^[31]^ respectively (Figure 7c). Given that GlyA binds PLP catalyzing formation of PLG in presence of glycine, we postulated that in addition to the formation of PLG, PbzB generates *N*‐pyridoxyl‐serine‐5‐phosphate (PLS) as an external aldimine formed by PLP and l‐serine (Figure 7e). An in‐depth analysis of the active sites revealed that all residues involved in substrate binding within GlyA and PbzB are superimposable (Figure 7f). Even Y55 and Y65 in GlyA are superimposable with T68 and H78 in PbzB, but these alterations might enlarge the binding pocket of PbzB. While Y55 and Y65 in GlyA perfectly coordinate PLG, T68 and H78 in PbzB open the space for the binding of PLS. Particularly, the substitution of Y55 by T68 may lead to a reduction of negative charges in the binding pocket and thus enable coordination of serine bound to PLP (Figure 7g–i). We constructed a site‐directed mutant of PbzB to investigate the key residues and attempt to shrink the capacity of the binding pocket. T68 and H78 were both mutated to Tyr as in GlyA (Figure S13). However, the PbzB T68Y H78Y mutant did not catalyze formation of any detectable amount of serine or 2‐hydroxymethylserine, suggesting that T68 and H78 residues are crucial to maintaining the function of PbzB.

Consistent with a general observation that central metabolism enzymes have higher *k*_cat_ and *k*_cat_/*K*_m_ values than secondary metabolism enzymes,^[35]^ the *k*_cat_ and *k*_cat_/*K*_m_ values of GlyA^[36]^ is 4200‐fold and 360 000‐fold larger than those of PbzB for l‐serine as a substrate. However, the *K*_m_ value of PbzB is ≈9‐fold lower than GlyA, suggesting that PbzB has a better affinity for l‐serine than GlyA. Although PbzB is much less efficient than GlyA, a low flux of 2‐hydroxymethylserine formation can be maintained by PbzB that satisfies the requirement of benzobactin production without disturbing primary metabolisms. Therefore, one could assume that benzobactin‐producing bacteria remodel an enzyme from the central amino acid metabolism to deliver a building block for the NRPS biosynthetic pathway. Since only **1** and no benzobactins were observed in the P_*BAD*_ *pbzA ΔpbzB* mutant (Figure 4a, trace iv), 2‐hydroxymethylserine furnished by PbzB is crucial for the downstream assembly line.

### Atypical Condensation Domain/Enzyme

The PbzD‐C_1_ domain is proposed to catalyze ester‐bond formation between benzoxazolinyl‐S‐T_1_ and 2‐hydroxymethylserinyl‐S‐T_2_ (Figure 5a). Interestingly, PbzD‐C_1_ is homologous to a heterocyclization domain as indicated by the phylogenetic analysis (Figure S14a) and sequence alignment (Figure S14b). Moreover, although 2‐hydroxymethylserine is a substrate containing both free NH_2_ and OH, the PbzD‐C_1_ domain appears to have a preference for using the OH as a nucleophile, which might result from the primary hydroxyl group having less steric hindrance than the α‐tertiary amine. The off‐loading of benzoxazolinyl‐2‐hydroxymethylserinyl‐S‐T_2_ to afford **3** is irrelevant to the PbzA‐TE domain but instead might be achieved by spontaneous hydrolysis, as indicated by the P_*BAD*_ *pbzA ΔpbzA* mutant that still produced **3** (Figure 4a, trace iii).

The absence of **2** in the P_*BAD*_ *pbzA ΔpbzA* mutant (Figure 4a, trace ii) also validated the necessity of the PbzA‐T_3_ domain for the incorporation of a second 2‐hydroxymethylserine into benzoxazolinyl‐2‐hydroxymethylserinyl‐S‐T_2_ (Figure 5a). Since no extra A domains are encoded within the BGC, presumably, the PbzA‐T_3_ domain “borrows” the activity of PbzD‐A_2_ domain that activates 2‐hydroxymethylserine (Figure 5a). Subsequently, the condensation between benzoxazolinyl‐2‐hydroxymethylserinyl‐S‐T_2_ and a 2‐hydroxymethylserinyl‐S‐T_3_ occurs under a putative free‐standing condensation enzyme encoded by *pbzI*, which is supported by the deletion of *pbzI* that led to loss of **2** (Figure 4a, trace vii). Interestingly, benzobactin C (**4**), as well as the as‐yet‐uncharacterized benzobactins‐628 and 1012, were lost in both the P_*BAD*_ *pbzA ΔpbzA* and P_*BAD*_ *pbzA ΔpbzI* mutants (Figure 4a, traces iii and vii). This indicates that the free‐standing NRPSs might be used iteratively resulting in dimerization or even tetramerization (Figure 5b).

From a functional point of view, PbzI appears to be a canonical condensation enzyme catalyzing amide‐bond formation and regioselectively uses the α‐tertiary amine with steric hindrance as a nucleophile, which differs from the PbzD‐C_1_ domain. However, phylogenetic analysis showed that PbzI, together with the other homologs that are encoded by putative benzobactin‐related BGCs, falls into a clade that is distinct from all other condensation domains/enzymes (Figure S14a). In particular, a closer inspection of the core motif C3 [xH(D)HxxxDxx] revealed that PbzI and its homologs do not have any histidine or aspartic acid residues in the second and third positions (Figure S14b). PbzI as a free‐standing enzyme with an atypical core motif C3 might be a new example to understand the catalytic mechanism of condensation domains/enzymes.

## Conclusion

We first characterized the biosynthesis of benzoxazolinate (*xsb*) in the entomopathogenic bacterium *X. szentirmaii* DSM 16338 (Figure 3a): the *xsb* BGC devoid of an ADIC synthase encoded gene “borrows” the ADIC precursor from the phenazine pathway (*xpz*); an FMN‐dependent and [Fe−S]‐containing dehydrogenase (XsbA, homologous to SgcG) is assumed to convert ADIC into OPA as previously described;^[7]^ and **1** is formed by intramolecular cyclization of OPA under the mediation of a putative acyl AMP‐ligase (XsbC). Given the structural uniqueness and the fascinating biological function of benzoxazolinate and benzoxazolinate‐containing natural products, we then performed a targeted BGC‐based genome mining by using the protein sequence of XsbC, and discovered that the orphan benzobactin pathway (*xvb*) in entomopathogenic bacteria *Xenorhabdus* and *Photorhabdus* ^[10]^ exists in diverse bacteria across Proteobacteria and Firmicutes, as exemplified by the *pbz* BGC in *P. chlororaphis* subsp. *piscium* DSM 21509 (Figure 2b). We homologously overexpressed the *pbz* BGC and discovered two new benzobactins (**3** and **4**), as well as benzobactins‐628 and 1012 that are yet to be characterized. Our recent whole‐genome‐sequencing of *X. vietnamensis* DSM 22392 revealed that the *xvb* cluster resides on a 51‐kb plasmid and is flanked with transposase and integrases, while the *pbz* cluster resides on the chromosome. This suggests that the benzobactin BGC is likely to spread horizontally in bacteria at a great evolutionary distance.

During the biosyntheses of benzobactins A–C (**2**–**4**), we observe that the anthranilate synthase component I (PbzF in the benzobactin pathway) and the ADIC synthase (PhzE in the phenazine pathway), which share low protein sequence similarity, have a synergistic positive effect on the production level of benzobactins (Figure 4c). Furthermore, our findings might indicate the immense biosynthetic potential of the *pbz* BGC, in which the serine hydroxymethyltransferase (PbzB) catalyzes mono‐ and bis‐hydroxymethylation to afford 2‐hydroxymethylserine which is an unusual building block in natural products (Figures 5a and 6a), and the free‐standing NRPS gene organization and non‐canonical condensation domain/enzyme (Figure S14) might have an iterative function.

## Conflict of interest

The authors declare no conflict of interest.

1

## Supporting information

As a service to our authors and readers, this journal provides supporting information supplied by the authors. Such materials are peer reviewed and may be re‐organized for online delivery, but are not copy‐edited or typeset. Technical support issues arising from supporting information (other than missing files) should be addressed to the authors.

Supporting Information

Supporting Information

## Data Availability

The data that support the findings of this study are available in the Supporting Information of this article.
