# Supplementary material for: Genome Mining Enabled by Biosynthetic Characterization Uncovers a Class of Benzoxazolinate‐Containing Natural Products in Diverse Bacteria
Source: Angew Chem Int Ed Engl. 2022 Nov 17;61(51):e202206106. doi: 10.1002/anie.202206106 (PMC10098953; doi:10.1002/anie.202206106)
Supplement: Supplementary file 1 — Supporting Information [file ANIE-61-0-s002.pdf]

Angewandte Chemie
Eine Zeitschrift der Gesellschaft Deutscher Chemiker

Supporting Information

Genome Mining Enabled by Biosynthetic Characterization Uncovers a Class of Benzoxazolinate-Containing Natural Products in Diverse Bacteria

Y.-M. Shi, J. J. Crames, L. Czech, K. A. J. Bozhüyük, Y.-N. Shi, M. Hirschmann, S. Lamberth, P. Claus, N. Paczia, C. Rückert, J. Kalinowski, G. Bange, H. B. Bode**

Supporting Information

Genome Mining Enabled by Biosynthetic Characterization Uncovers a Class of Benzoxazolinate-Containing Natural Products in Diverse Bacteria

Yi-Ming Shi*⊥[1,2], Jan J. Crames⊥[1,2], Laura Czech[3], Kenan A. J. Bozhüyük[1,2], Yan-Ni Shi[1,2], Merle Hirschmann[2], Stefanie Lamberth[2], Peter Claus[4], Nicole Paczia[4], Christian Rückert[5], Jörn Kalinowski[5], Gert Bange[3], Helge B. Bode*[1,2,6,7]

[1]Department of Natural Products in Organismic Interactions, Max Planck Institute for Terrestrial Microbiology, 35043 Marburg, Germany

[2]Molecular Biotechnology, Department of Biosciences, Goethe University Frankfurt, 60438 Frankfurt am Main, Germany

[3]Center for Synthetic Microbiology (SYNMIKRO) & Faculty of Chemistry, Philipps University of Marburg, 35043 Marburg, Germany

[4]Core Facility for Metabolomics and Small Molecule Mass Spectrometry, Max Planck Institute for Terrestrial Microbiology, 35043 Marburg, Germany

[5]Microbial Genomics and Biotechnology, Center for Biotechnology (CeBiTec), Bielefeld University, 33615 Bielefeld, Germany

[6]Senckenberg Gesellschaft für Naturforschung, 60325 Frankfurt am Main, Germany

[7]Chemical Biology, Department of Chemistry, Philipps University of Marburg, 35043 Marburg, Germany

⊥These authors contributed equally

*Corresponding author e-mail: yi-ming.shi@mpi-marburg.mpg.de; helge.bode@mpi-marburg.mpg.de

Methods

General experimental procedures

All chemicals were purchased from Sigma-Aldrich, Acros Organics, or Iris BIOTECH. Isotope-labeled chemicals were purchased from Cambridge Isotope Laboratories, Inc. Genomic DNA of selected *Xenorhabdus* and *Pseudomonas* strains were isolated using the Qiagen Gentra Puregene Yeast/Bact Kit. DNA polymerases (Taq, Phusion, and Q5) and restriction enzymes were purchased from New England Biolabs or Thermo Fisher Scientific. DNA primers were purchased from Eurofins MWG Operon. DNA fragments were purchased from Twist Bioscience. PCR amplifications were carried out on thermocyclers (SensoQuest). Polymerases were used according to the manufacturers' instructions. DNA purification was performed from 1% TAE agarose gel using Invisorb® Spin DNA Extraction Kit (STRATEC Biomedical AG). Plasmids in *E. coli* were isolated by alkaline lysis. HPLC–UV–MS analysis was conducted on an UltiMate 3000 system (Thermo Fisher) coupled to an AmaZonX mass spectrometer (Bruker) with an ACQUITY UPLC BEH C18 column (130 Å, 2.1 mm × 100 mm, 1.7 µm particle size, Waters) at a flow of 0.6 mL/min (5–95% acetonitrile/water with 0.1% formic acid, v/v, 16 min, UV detection wavelength 190–800 nm). HPLC–UV–HRMS analysis was conducted on an UltiMate 3000 system (Thermo Fisher) coupled to an Impact II qTof mass spectrometer (Bruker) with an ACQUITY UPLC BEH C18 column (130 Å, 2.1 mm × 100 mm, 1.7 µm particle size, Waters) at a flow of 0.4 mL/min (5–95% acetonitrile/water with 0.1% formic acid, v/v, 16 min, UV detection wavelength 190–800 nm). HPLC purification was performed on preparative and semipreparative Agilent 1260 systems coupled to a DAD and a single quadrupole detector with a C18 ZORBAX Eclipse XDB column (9.4 mm × 250 mm, 5 µm, 3 mL/min; 50 mm x 250 mm, 10 µm, 40 mL/min). Freeze drying was performed by BUCHI Lyovapor™ L-300 Continuous. NMR experiments were acquired on a Bruker AVANCE 500 or 600 MHz spectrometer equipped with a 5 mm cryoprobe.

Sequencing

Long and short DNA reads were generated by Nanopore and Illumina sequencing, respectively. For library preparation, a TruSeq DNA PCR-free high-throughput library prep kit (Illumina) and the SQK-LSK109 ligation sequencing kit (Oxford Nanopore Technologies, ONT) were used without prior shearing of the DNA. To generate the short reads, a 2 × 300-nucleotide run (MiSeq reagent kit v3, 600 cycles) was executed. The long reads were generated on a GridION platform using an R9.4.1 flow cell. Base-calling and demultiplexing were performed using Guppy v4.0.11 (ref. [1]). Both data sets were assembled using Unicycler v0.4.6. The region of interest was identified using antiSMASH (ref. [2]) to be located on a plasmid with a size of 182,126 bp in Xenorhabdus vietnamensis DSM 22392.

Strain and culture conditions

Wild-type strains and the mutants thereof and *E. coli* (Table S1) were cultivated on lysogeny broth (LB) agar plates at 30 ^{o}C overnight and were subsequently inoculated into liquid LB culture at 30 ^{o}C with shaking at 200 rpm. For compound production, the overnight LB culture of a mutant was transferred into 5 mL XPP medium[3] (1:100, v/v) with 2% (v/v) of Amberlite™ XAD-16 resins, 0.1 % of L-arabinose as an inducer, and selective antibiotics such as ampicillin (Am, 100 µg/mL), kanamycin (Km, 50 µg/mL), or chloramphenicol (Cm, 34 µg/mL) at 30 ^{o}C with shaking at 200 rpm.

Culture extraction and HPLC-UV-MS analysis

The XAD-16 resins were collected after 72 h and extracted with 5 mL methanol. The solvent was dried under rotary evaporators, and the dried extract was resuspended in 500 µL methanol, of which 5 µL was injected and analyzed by HPLC-UV-MS or HPLC-UV-HRMS. Unless otherwise specified, HPLC-UV-MS and HPLC-UV-HRMS chromatograms in the figures are shown on the same scale. Relative quantifications of benzobactins in *P. chlororaphis* P_{BAD} *pzbA*, *P. chlororaphis* P_{BAD} *pzbA ΔpbzF*, and *P. chlororaphis* P_{BAD} *pzbA ΔphzE* were measured by the peak area of extracted ion chromatograms (EICs) using the Bruker Compass DataAnalysis program. Peak areas were normalized by OD_{600} values at the harvesting time point.

Construction of insertion mutants

A 500–800-bp upstream of the target gene (*xsbA* and *pbzA*) was amplified with a corresponding primer pair listed in Table S3. The resulting fragments were cloned using Hot Fusion[4] into pCEP_kan or pCEP_cm backbone that was amplified by pCEP_Fw and pCEP_Rv. After the transformation of the constructed plasmid into *E. coli* S17-1 *λ* pir, clones were verified by PCR with primers pCEP-Ve-Fw and pDS132-Ve-Rv. The wild-type strain (recipient) was mated with *E. coli* S17-1 *λ* pir (donor) carrying constructed plasmids. Both strains were grown in LB medium to an OD_{600} of 0.6 to 0.7, and the cells were washed once with fresh LB medium. Subsequently, the donor and recipient strains were mixed on an LB agar plate in ratios of 1:3 and 3:1, and incubated at 37°C for 3 h followed by incubation at 30°C for 21 h. After that, the bacterial cell layer was harvested with an inoculating loop and resuspended in 2 mL fresh LB medium. 200 µL of the resuspended culture was spread out on an LB agar plate with ampicillin/kanamycin (or ampicillin/chloramphenicol) and incubated at 30°C for 2 days. Individual insertion clones were cultivated and analyzed by HPLC-UV-HRMS, and the genotype of all mutants was verified by plasmid- and genome-specific primers.

Construction of deletion mutants

A ~1000-bp upstream and a ~1000-bp downstream fragments (mutations were introduced by primers) of a target gene (*xsbB*, *xsbC*, *xsbD*, *pbzA*, *pbzB*, *pbzD*, *pbzF*, *pbzG*, *pbzI*, and *phzE*) were

amplified using primer pairs listed in Table S3. The amplified fragments were fused using the complementary overhangs introduced by primers and cloned into the pCKcipB or pEB17_KM vector (linearized with PstI and BglII) by Hot Fusion.[4] Transformation of *E. coli* S17-1 *λ* pir with the resulting plasmid and conjugation with a wild-type strain or mutant, as well as the generation of double crossover mutants via counterselection on LB plates containing 6% sucrose. Deletion mutants were verified via PCR using primer pairs listed in Table S3, which yielded a ~2000-bp fragment for mutants genetically equal to the WT strain and a ~1000-bp fragment for the desired deletion mutant.

Heterologous expression of *xsb* BGC

All plasmids carrying target genes for heterologous expression were constructed via Hot Fusion.[4] The biosynthetic gene cluster, *xsbABCDE*, was cloned into pCOLADuet-1. The ADIC synthase encoded gene in the *xpz* BGC (*xpzC*) was cloned into pACYCDuet-1. *xsbA* and *xpzC* were constructed separately into two multiple cloning sites of pACYCDuet-1. *xsbC* was cloned into pCOLADuet-1. *E. coli* BL21(DE3) was transformed with plasmids for (co-)expression.

Homology modeling

The protein sequence of XsbC (NCBI: WP_038235707.1) from *X. szentirmaii* and the crystal structure coordinates of both NatL2 (PDB ID: 6SIY)[5] and PtmA2 (PDB ID: 5UPS)[6] were loaded into the Molecular Operating Environment (MOE) 2019.0102 (ref[7]). The sequence identities of XsbC with NatL2 and PtmA2 are 19.8 % and 13.2 %, respectively. The alignments made by MOE were inspected and manually corrected if necessary. The alignment used for modelling is depicted in Figure S3, involving acyl-AMP ligases [NatL2, BomJ (NCBI: ALE27502.1; ref[8]), and PtmA1 (NCBI: ACO31267.1; ref[6])], acyl-CoA ligase (PtmA2), and A domains [DltA (PDB ID: 3FCC; ref[9]) and PheA (PDB ID: 1AMU; ref[10])]. Prior to modeling XsbC, the crystal structures of both NatL2 (6SIY) and PtmA2 (5UPS) were prepared (e.g. wrong protonation, chirality, and hybridization). A series of ten models each was constructed with MOE using a Boltzmann-weighted randomized procedure combined with specialized logic for the handling of sequence insertions and deletions.[11, 12] The model with the best packing quality function was selected for full energy minimization. Using Amber14:EHT MOE packing scores for models calculated with NatL2 (6SIY) and PtmA2 (5UPS) as templates for homology modeling have been 2.2527 and 2.3590, respectively. The stereochemical qualities of the model were assessed using Ramachandran plots and calculating the Root-Mean-Square-Deviation (RMSD) values of the superposed Cα-atoms of the model with its respective template structure (Figures S5–8).

Isotope labeling experiments

The cultivation of strains for labeling experiments was carried out as described above.[13] The cell pellets of the 100 μL overnight culture were washed once with 100-μL ISOGRO® ^{15}N medium before being transferred into the 5-mL ISOGRO® ^{15}N medium culture. For the purpose of inverse feedings, additional unlabeled L-serine or glycine was added into the ^{15}N medium culture at a final concentration of 1 mM.

Isolation and purification

2% of XAD-16 resins from a 3 L LB culture of *X. szentirmaii* P_{BAD} *xsbA* mutant induced by L-arabinose were harvested after 72 h of incubation at 30 °C with shaking at 120 rpm, and were washed with water and extracted with methanol (3 × 500 mL) to yield a crude extract 3.5 g. The extract was subjected to a Sephadex LH-20 column eluted with MeOH and afforded seven fractions. Fraction 3 (55.6 mg) was purified by semipreparative HPLC using an acetonitrile/water gradient (0.1% formic acid) 0–30 min, 15–50% to afford **1** (2.4 mg).

4% of XAD-16 resins from a 12 L culture cells of *P. chlororaphis* P_{BAD} *pbzA ΔpbzI* mutant induced by L-arabinose were harvested after 72 h of incubation at 30 °C with shaking at 120 rpm, and were washed with water and extracted with methanol (3 × 2 L) to yield a crude extract 95.4 g after evaporation. The extract was dissolved in methanol and was subjected to preparative HPLC using an acetonitrile/water gradient (0.1% formic acid) 0–18 min, 5–59% to afford ten fractions. Fraction 4 (70.0 mg) was further purified by semipreparative HPLC using an acetonitrile/water gradient (0.1% formic acid) 0–35 min, 5–95% to afford **3** (2.6 mg).

NMR spectroscopy

^{1}H and ^{13}C NMR, ^{1}H-^{13}C heteronuclear single quantum coherence (HSQC), ^{1}H-^{13}C heteronuclear multiple bond correlation (HMBC), and ^{1}H-^{1}H correlation spectroscopy (COSY) were measured. Chemical shifts (δ) were reported in parts per million (ppm) and referenced to the solvent signals. Data are reported as follows: chemical shift, multiplicity (br = broad, s = singlet, d = doublet, t = triplet, dd = doublet of doublet, m = multiplet, and ov = overlapped), and coupling constants in Hertz (Hz).

In vitro enzymatic assays of PbzB

pbzB with an N-terminal His-SUMO-tag was cloned into a pET11a vector. *E. coli* BL21(DE3) was transformed with the resulting plasmid. 10 mL overnight culture carrying the plasmid was transferred to 500 mL LB medium with ampicillin (Am, 100 μg/mL). The strain was grown to an OD_{600} of 0.8 at 37 °C, and then 0.5 mM IPTG for induction was added into the culture, followed by incubation at 22 °C for 24 h. The cells were collected by centrifugation (10,000 r.p.m., 15 min, 4 °C). Cell lysis was performed by resuspending the pellet in 100 mL BugBuster® (primary amine-free)

Extraction Reagent with 1 µL of Benzonase® Nuclease, 14 mg of cOmplete™ EDTA-free protease inhibitor, and lysozyme (200 µg/mL), followed by incubation at 4 °C for 45 min. Cell debris was removed by centrifugation at 20,000 x*g* for 30 min and the protein was purified by using Ni^{2+} affinity chromatography.

The reaction mixture (100 µL) contained 3.2 µM of PbzB, 1 mM substrate (glycine, L-serine, and D-serine), 500 µM mTHF, 5 µM PLP, and 50 mM potassium phosphate buff pH 7.5. After incubation at 30 °C for 1 h, the reaction was quenched by adding 100 µL of acetonitrile. Products were analyzed using HPLC–UV–HRMS conducted on an UltiMate 3000 system (Thermo Fisher) coupled to an Impact II qTof mass spectrometer (Bruker) with an ACQUITY UPLC BEH Amide column (130 Å, 2.1 mm × 50 mm, 1.7 µm particle size, Waters) at a flow of 0.4 mL/min (5–50% water/acetonitrile with 0.1% formic acid, v/v, 5 min, 90% water/acetonitrile with 0.1% formic acid, v/v, 2.1 min, UV detection wavelength 190–800 nm. 2-Hydroxymethylserine as a standard compound was prepared at different concentrations and these samples were measured by HPLC–UV–HRMS with the above-mentioned method to obtain a standard curve (Figures S27 and S28).

For the identification of the favored substrate of PbzB, a reaction mixture (100 µL) containing 10 µM of PbzB, 2 mM substrate (glycine, L-serine, or D-serine), 500 µM mTHF, 25 µM PLP, and 50 mM potassium phosphate buffer pH 7.5 was prepared, and incubated at 30 °C for 2.5 h. The determination of the steady-state kineticsof PbzB was performed by preparing reaction mixtures (100 µL) containing 4 µM of PbzB, 50 µM, 100 µM, 200 µM, 300 µM, 1000 µM, 2000 µM, 3000 µM, or 4000 µM substrate (D-/L-serine), 200 µM mTHF, 10 µM PLP, and 50 mM potassium phosphate buffer pH 7.5, which were incubated at 30 °C for 1 h. The reaction was quenched by adding 100 µL of acetonitrile. The quantification of 2-hydroxymethylserine was performed using LC-MS/MS. The chromatographic separation was performed on an Agilent Infinity II 1290 HPLC system using a ZicHILIC SeQuant column (150 × 2.1 mm, 3.5 µm particle size, 100 Å pore size) connected to a ZicHILIC guard column (20 × 2.1 mm, 5 µm particle size) (Merck KgAA) at a constant flow rate of 0.3 mL/min with mobile phase A being 0.1 % formic acid in 99:1 water:acetonitrile (Honeywell, Morristown, New Jersey, USA) and phase B being 0.1 % formic acid 99:1 water:acetonitrile (Honeywell, Morristown, New Jersey, USA) at 25° C. The injection volume was 1 µL. The mobile phase profile consisted of the following steps and linear gradients: 0–8 min from 80 to 60% B; 8–10 min from 60 to 10% B; 10–12 min constant at 10% B; 12–12.1 min from 10 to 80% B; 12.1 to 14 min constant at 80% B. An Agilent 6495 ion funnel mass spectrometer was used in positive mode with an electrospray ionization source and the following conditions: ESI spray voltage 4500 V, nozzle voltage 1500 V, sheath gas 400° C at 12 L/min, nebulizer pressure 30 psig, and drying gas 250° C at 11 L/min. Compounds were identified based on their mass transition and retention time compared to standards. Chromatograms were integrated using MassHunter software (Agilent, Santa Clara, CA, USA). Absolute concentrations were calculated based on an external calibration curve prepared in sample matrix. Mass transitions, collision energies, Cell accelerator voltages,

fragmentor voltages and Dwell times have been optimized using chemically pure standards. Parameter settings of all targets are given in the table below.

Q1	Q3	CE (V)	CA(V)	Fragmentor	Dwell time (msec)
136	118.1	7	5	380	80
136	90	10	5	380	80
136	72.1	18	5	380	80
136	60.1	20	5	380	80

PbzB crystallization

E. coli BL21(DE3) cells transformed with pET11a-SUMO1_*pbzB* with a His-SUMO tag were grown in 4 L LB medium supplemented 100 µg mL^{-1} ampicillin and 1% lactose to induce expression of the recombinant fusion protein and incubated for 18 h at 30°C in an aerial shaker. After harvesting (4,500 rpm, 15 min, 4°C), cells were lysed using a Microfluidizer (M110-L, Microfluidics). The lysis buffer contained 20 mM HEPES-Na (pH 8.0), 250 mM NaCl, 20 mM KCl, 20 mM $MgCl_2$, and 50 mM imidazole. Cell debris was then removed by high-speed centrifugation for 20 min at 20,000 rpm at 4°C. PbzB was then purified at 10°C by Ni-ion affinity chromatography and eluted using a lysis buffer supplemented with 250 mM imidazole. 250 Units of SUMO protease (Sigma-Aldrich) were added to the PbzB fusion protein elution (15 mL) and incubated at RT to remove the His_6 affinity purification and the SUMO solubility tag. After analysis of the cleavage efficiency using SDS-PAGE, PbzB was concentrated to 2 mL using an Amicon concentrator with a cutoff at 30 kDa. Size exclusion chromatography was performed on an S200 XK16 column (GE Healthcare). The SEC buffer consisted of 20 mM HEPES-Na (pH 7.5), 200 mM NaCl, 20 mM KCl, and 20 mM $MgCl_2$. Samples of each peak were analyzed on an SDS-PAGE and fractions containing cleaved PbzB protein were pooled and concentrated to a final concentration of 600 µM. The protein solution showed a yellow color.

Crystallization was performed by the sitting-drop method at 20 °C in 250 nL drops consisting of equal parts of protein and precipitation solutions. Protein solutions of 300-600 µM were used for crystallization. Crystallization conditions were: 0.1 M KCl, 0.1 M HEPES pH 7.5, 15% (w/v) PEG 6000. Prior to data collection, crystals were flash-frozen in liquid nitrogen using a cryo-solution that consisted of mother-liquor supplemented with 20% (v/v) glycerol. Data were collected under cryogenic conditions at the European Synchrotron Radiation Facility (Grenoble, France)[14]. Data were processed with XDS and scaled with XSCALE[15]. All structures were determined by molecular replacement with PHASER[16] manually built in COOT[17], and refined with PHENIX[18]. The search model for the PbzB structures was the glycine hydroxymethyltransferase from *Acinetobacter baumannii* (PDB 5VMB). Figures were prepared with Pymol (www.pymol.org)[19, 20].

Data availability

All data generated or analyzed in this study are available within the article and its Supplementary Information files. The genome sequence data involved in this study are accessible in the NCBI GenBank database under accession numbers NIBV00000000 (*X. szentirmaii* DSM 16338), OM622254 (the plasmid in *X. vietnamensis* DSM 22392), and LT629761 (*P. chlororaphis* subsp. *piscium* DSM 21509). Crystallographic data have been deposited in the Protein Data Bank (https://www.rcsb.org) under the PDB ID 7QCW.

Table S1. Strains used in this study.

Strain	Genotype/Description	Reference
BL21(DE3)	F^-, *ompT*, *gal*, *dcm*, *hsdSB*(*rB- mB-*), *lon*, *λ*(*DE3* [*lacI*, *lacUV5-T7*, *gene1*, *ind1*, *sam7*, *nin5*])	Invitrogen
BL21 pCOLA *xsbABCDE*	BL21(DE3), pCOLADuet-1 *Xsze_RS17950-RS17930*, *T7* promoter, Km^r	This study
BL21 pCOLA *xsbABCDE* pACYC_*xpzC*	BL21(DE3), pCOLADuet-1 *Xsze_RS17950-RS17930*, pACYCDuet-1 *Xsze_RS10860*, *T7* promoter, Km^r, Cm^r	This study
BL21 pACYC *xsbA xpzC*	BL21(DE3), pACYCDuet-1 *Xsze_RS17950 Xsze_RS10860*, *T7* promoter, Cm^r	This study
BL21 pACYC *xsbA xpzC* pCOLA *xsbC*	BL21(DE3), pACYCDuet-1 *Xsze_RS17950 Xsze_RS10860*, pCOLADuet-1 *Xsze_RS17940*, *T7* promoter, Cm^r, Km^r	This study
S17-1 *λ* pir	Tpr, Smr, *recA*, *thi*, *pro*, $hsdRM^+$, RP4::2-Tc::Mu::Km, Tn7, *λ* pir	Invitrogen
X. szentirmaii DSM 16338	wild type	[21]
X. szentirmaii P_{BAD} *xsbA*	*X. szentirmaii* pCEP *Xsze_RS17950*, *araBAD* promoter, Km^r	This study
X. szentirmaii P_{BAD} *xsbA* Δ*xsbB*	*X. szentirmaii* pCEP *Xsze_RS17950*, Δ*Xsze_RS17945*, *araBAD* promoter, Km^r	This study
X. szentirmaii P_{BAD} *xsbA* Δ*xsbC*	*X. szentirmaii* pCEP *Xsze_RS17950*, Δ*Xsze_RS17940*, *araBAD* promoter, Km^r	This study
X. szentirmaii P_{BAD} *xsbA* Δ*xsbD*	*X. szentirmaii* pCEP *Xsze_RS17950*, Δ*Xsze_RS17935*, *araBAD* promoter, Km^r	This study
X. szentirmaii P_{BAD} *xsbA* Δ*xpzC*	*X. szentirmaii* pCEP *Xsze_RS17950*, Δ*Xsze_RS10860*, *araBAD* promoter, Km^r	This study
P. chlororaphis subsp. *piscium* DSM 21509	wild type	[22]
P. chlororaphis P_{BAD} *pbzA*	*P. chlororaphis* subsp. *piscium* pCEP *BLU06_RS20875*, *araBAD* promoter, Km^r	This study
P. chlororaphis P_{BAD} *pbzA* Δ*pbzA*	*P. chlororaphis* subsp. *piscium* pCEP *BLU06_RS20875*, Δ*BLU06_RS20875*, *araBAD* promoter, Km^r	This study
P. chlororaphis P_{BAD} *pbzA* Δ*pbzB*	*P. chlororaphis* subsp. *piscium* pCEP *BLU06_RS20875*, Δ*BLU06_RS20870*, *araBAD* promoter, Km^r	This study
P. chlororaphis P_{BAD} *pbzA* Δ*pbzD*	*P. chlororaphis* subsp. *piscium* pCEP *BLU06_RS20875*, Δ*BLU06_RS20855*, *araBAD* promoter, Km^r	This study
P. chlororaphis P_{BAD} *pbzA* Δ*pbzF*	*P. chlororaphis* subsp. *piscium* pCEP *BLU06_RS20875*, Δ*BLU06_RS20845*, *araBAD* promoter, Km^r	This study
P. chlororaphis P_{BAD} *pbzA* Δ*pbzG*	*P. chlororaphis* subsp. *piscium* pCEP *BLU06_RS20875*, Δ*BLU06_RS20840*, *araBAD* promoter, Km^r	This study
P. chlororaphis P_{BAD} *pbzA* Δ*pbzI*	*P. chlororaphis* subsp. *piscium* pCEP *BLU06_RS20875*, Δ*BLU06_RS20830*, *araBAD* promoter, Km^r	This study
P. chlororaphis P_{BAD} *pbzA* Δ*phzE*	*P. chlororaphis* subsp. *piscium* pCEP *BLU06_RS20875*, Δ*BLU06_RS09605*, *araBAD* promoter, Km^r	This study
P. chlororaphis P_{BAD} *pbzA* Δ*pbzF*Δ*phzE*	*P. chlororaphis* subsp. *piscium* pCEP *BLU06_RS20875*, Δ*BLU06_RS20845, ΔBLU06_RS09605*, *araBAD* promoter, Km^r	This study
BL21 pET *pbzB*	BL21(DE3), pET11a-SUMO1 *pbzB*, *T7* promoter, Amp^r	This study
BL21 pET *pbzB* T68Y H79Y	BL21-Gold (DE3), pET11a-SUMO1 *pbzB*, T68Y, H79Y, *T7* promoter, Amp^r	This study

Table S2. Plasmids used in this study.

Plasmid	Genotype/Description	Reference
pCEP_kan	R6Kγ *ori*, *oriT*, *araC*, *araBAD* promoter, Km^r	[23]
pEB17_KM	pDS132 derivative with an additional *Bgl*II recognition site, R6Kγ *ori*, *oriT*, *cipB* promoter, Km^r	[3]
pCKcipB	pDS132 derivative with an additional *Bgl*II recognition site, R6Kγ *ori*, *oriT*, *cipB* promoter, Cm^r	[24]
pCOLADuet-1	*T7* promoter, Km^r	Novagen
pACYCDeut-1	*T7* promoter, Cm^r	Novagen
pET11a-modified	modified from pET11a, the operon under the control of T7 promoter was modified by introducing an N-terminal His_6-Smt3 tag, *ori pBR322*, Amp^r, *Ulp1* cleavage site	addgene
pCOLA *xsbABCDE*	pCOLADuet-1 *Xsze_RS17950-RS17930*, *T7* promoter, Km^r	This study
pACYC *xpzC*	pACYCDuet-1 *Xsze_RS10860*, *T7* promoter, Cm^r	This study
pACYC *xsbA xpzC*	pACYCDuet-1 *Xsze_RS17950 Xsze_RS10860*, *T7* promoter, Cm^r	This study
pCOLA *xsbC*	pCOLADuet-1_*Xsze_RS17940*, *T7* promoter, Km^r	This study
pCEP *xsbA*	pCEP *Xsze_RS15520'*, *araBAD* promoter, Cm^r	This study
pCKcipB *xsbB*	pCKcipB *Xsze_RS17945'*, Cm^r	This study
pCKcipB *xsbC*	pCKcipB *Xsze_RS17940'*, Cm^r	This study
pCKcipB *xsbD*	pCKcipB *Xsze_RS17935'*, Cm^r	This study
pCKcipB *xpzC*	pCKcipB *Xsze_RS10860'*, Cm^r	This study
pCEP *pbzA*	pCEP *BLU06_RS20875'*, *araBAD* promoter, Cm^r	This study
pEB17 *pbzA*	pEB17 *BLU06_RS20875'*, Km^r	This study
pEB17 *pbzB*	pEB17 *BLU06_RS20870'*, Km^r	This study
pEB17 *pbzD*	pEB17 *BLU06_RS20855'*, Km^r	This study
pEB17 *pbzF*	pEB17 *BLU06_RS20845'*, Km^r	This study
pEB17 *pbzG*	pEB17 *BLU06_RS20840*, Km^r	This study
pEB17 *pbzI*	pEB17 *BLU06_RS20830*, Km^r	This study
pEB17 *phzE*	pEB17 *BLU06_RS09605*, Km^r	This study
pET11a *pbzB*	pET11a-modified, *BLU06_RS20870*, *T7* promoter, His_6-Smt3, Amp^r	This study
pET11a *pbzB* T68Y H79Y	pET11a-modified, *BLU06_RS20870*, T68Y, H79Y, *T7* promoter, His_6-Smt3, Amp^r	This study

Table S3. Primers and DNA fragments used in this study.

Primer	Sequence (5'-3')	Purpose
pCEP_Fw<br>pCEP_Rv	ATGTGCATGCTCGAGCTC<br>ATGCTAGCCTCCTGTTAGC	Backbone amplification of pCEP_kan and pCEP_cm
pCEP-Ve-Fw<br>pDS132-Ve-Rv	GCTATGCCATAGCATTTTTATCCATAAG<br>ACATGTGGAATTGTGAGCGG	Verification of the pCEP_kan and pCEP_cm constructs
YS19-Fw4<br>YS19-Rv4	TTGTTTAACTTTAATAAGGAGATATACC_ATGACAAATAAAATCAGATATGTCG<br>CAATACGATTACTTTCTGTTCGACTTAAGCA_TCATTCATCAATCCTGTTCGTGAG	Amplification of *xsbABCDE*
YS20-Fw<br>YS20-Rv	TTGTTTAACTTTAATAAGGAGATATACC_ATGAGTAGCCTTCTGTCTTCGATTA<br>CAATACGATTACTTTCTGTTCGACTTAAGCA_TTACGATACTGTTAGGCATTTTTCT	Amplification of *xpzC*
YS26-Fw<br>YS26-Rv	TTAAGTATAAGAAGGAGATATACAT_ATGACAAATAAAATCAGATATGTCGC<br>CAGCGGTGGCAGCAGCCTAGGTTAA_CTAGCGCATATCGAGCTTTGATG	Amplification of *xsbA*
YS27-Fw<br>YS27-Rv	TTTAACTTTAATAAGGAGATATACC_ATGACTCATAAGCAATTACACCGG<br>GATTACTTTCTGTTCGACTTAAGCA_TCATAGATTCGAATTCCTCTTATC	Amplification of *xsbC*
MHp282<br>MHp283	GGCTAACAGGAGGCTAGCAT_ATGACAAATAAAATCAGATATGTCG<br>GCCGTTTAAACATTTAAATCTGCAG_GATGGGACGTAGAATTCACC	Promoter insertion of *xsbA*
YS-Bz-D3-Fw<br>YS-Bz-D3-Rv<br>YS-Bz-D4-Fw<br>YS-Bz-D4-Rv	CCTCTAGAGTCGACCTGCAG_ATAACCGAACACTCACCAACAAG<br>GCTAACACGTTGGCAAATTGCTA_ACATTTCAGATGCTTAGGCAGTG<br>AACACTGCCTAAGCATCTGAAATGT_TAGCAATTTGCCAACGTGTTAGC<br>TCCCGGGAGAGCTCAGATCT_GATTGCATGTGCTTCTTGTGAAC	Deletion of *xsbB*
YS-Bz-D5-Fw<br>YS-Bz-D5-Rv<br>YS-Bz-D6-Fw<br>YS-Bz-D6-Rv	CCTCTAGAGTCGACCTGCAG_GGACGGTCGGATTCATTAGTG<br>TCCTCTTATCTCTAATACGAGCATG_GGAATACCGTTAAGGCTCTTGAAT<br>TATTCAAGAGCCTTAACGGTATTCC_CATGCTCGTATTAGAGATAAGAGGAATTC<br>TCCCGGGAGAGCTCAGATCT_GTAATAAGAATGGTGAGTTCAGAGGG	Deletion of *xsbC*
YS-Bz-D7-Fw<br>YS-Bz-D7-Rv<br>YS-Bz-D8-Fw<br>YS-Bz-D8-Rv	CCTCTAGAGTCGACCTGCAG_CGTCCAGACTGATTACTGATCTTG<br>ACTCATAATAATATCATCACCGAGT_CAACCTCGTTAATACATGCAGGC<br>CGGCCTGCATGTATTAACGAGGTTG_ACTCGGTGATGATATTATTATGAGTACC<br>TCCCGGGAGAGCTCAGATCT_GCATCAGTAACATTGCTGAATAACC	Deletion of *xsbD*
YS-Bz-D9-Fw<br>YS-Bz-D9-Rv<br>YS-Bz-D10-Fw<br>YS-Bz-D10-Rv	CCTCTAGAGTCGACCTGCAG_AATGCGATGGTCGCGGTATTATTC<br>TTTGCTTCCGGCAAGTTGTGTGTGT_GTTCAGGCGAAGCGATAATCGAA<br>TCTTCGATTATCGCTTCGCCTGAAC_ACACACACAACTTGCCGGAAGC<br>TCCCGGGAGAGCTCAGATCT_CTCACAGACATCGCCATTCCGTG	Deletion of *xpzC*
YS-Pcp-Bz1-Fw<br>YS-Pcp-Bz1-Rv	TTTGGGCTAACAGGAGGCTAGCAT_ATGACTGAAAACATCCAGTTCGATG<br>TCTGCAGAGCTCGAGCATGCACAT_TGACAGCCTTTCGTGGCTATC	Promoter insertion of *pbzA*
YS-Pcp-Bz-D17-Fw<br>YS-Pcp-Bz-D17-Rv	CCTCTAGAGTCGACCTGCAG_AGCGTCCGAGCTGTTCATCAAC<br>GATTGACTGTCATTACTTGTTATCC_CCAGGTCTGGTCCGAAG	Deletion of *pbzA*

	TGATTTC	
YS-Pcp-Bz-D18-Fw	CGAAATCACTTCGGACCAGACCTGG_GGATAACAAGTAATGACAGTCAATCATC	
YS-Pcp-Bz-D18-Rv	TCCCGGGAGAGCTCAGATCT_GTAGTCAGCGATCGGATACTTCAC	
YS-Pcp-Bz-D3-Fw	CCTCTAGAGTCGACCTGCAG_AACATCCAGTTCGATGAACAAGC	Deletion of *pbzB*
YS-Pcp-Bz-D3-Rv	CTCGGCTTCCGCACAGAATTGCTTG_CGGTTGATGATTGACTGTCATTAC	
YS-Pcp-Bz-D4-Fw	GTAATGACAGTCAATCATCAACCG_CAAGCAATTCTGTGCGGAAG	
YS-Pcp-Bz-D4-Rv	TCCCGGGAGAGCTCAGATCT_AGTGTTCCGACACTATCCATTC	
YS-Pcp-Bz-D7-Fw	CCTCTAGAGTCGACCTGCAG_AAGACCGTACTGGCCATGGAAAAC	Deletion of *pbzD*
YS-Pcp-Bz-D7-Rv	AGTGGAGTCTTTCATGTCATTCCTC_TCGTCATGGATTCCCAGGATTAG	
YS-Pcp-Bz-D8-Fw	GGCTAATCCTGGGAATCCATGACGA_GAGGAATGACATGAAAGACTCCACTC	
YS-Pcp-Bz-D8-Rv	TCCCGGGAGAGCTCAGATCT_AAGAATCTCCGCCAGATAGTTGGTC	
YS-Pcp-Bz-D9-Fw	CCTCTAGAGTCGACCTGCAG_CACTTCTCCGAGACCAACTATCTG	Deletion of *pbzF*
YS-Pcp-Bz-D9-Rv	CGGGAAAACGACTTGATCTTCGCTC_TACATCCTGCTCGATGGCCATTC	
YS-Pcp-Bz-D10-Fw	GGGAATGGCCATCGAGCAGGATGTA_GAGCGAAGATCAAGTCGTTTTCC	
YS-Pcp-Bz-D10-Rv	TCCCGGGAGAGCTCAGATCT_AAGGAAAATGCGCCAAAAATCACTAC	
YS-Pcp-Bz-D11-Fw	CCTCTAGAGTCGACCTGCAG_AATGGCCATCGAGCAGGATGTAC	Deletion of *pbzG*
YS-Pcp-Bz-D11-Rv	GGCTAATGAGTTACGGATCATTTTG_GAGTAGTACTGCTCGATGACATGC	
YS-Pcp-Bz-D12-Fw	TGCATGTCATCGAGCAGTACTACTC_CAAAATGATCCGTAACTCATTAGCC	
YS-Pcp-Bz-D12-Rv	TCCCGGGAGAGCTCAGATCT_GTAGTCTTGCAGCAACAGTACAC	
YS-Pcp-Bz-D13-Fw	CCTCTAGAGTCGACCTGCAG_AAACGCTCGGAAAACGGCATTATTC	Deletion of *pbzI*
YS-Pcp-Bz-D13-Rv	GGGATACAAGGCTGCCGGTTCTATG_ACCAGTTGTTCTCCTTCGATGTC	
YS-Pcp-Bz-D14-Fw	TCGACATCGAAGGAGAACAACTGGT_CATAGAACCGGCAGCCTTGTATC	
YS-Pcp-Bz-D14-Rv	TCCCGGGAGAGCTCAGATCT_TCATCAGACCCGGATATTATTGTCC	
JJC-PCP-Bz-phzE-D1_Fw	CCTCTAGAGTCGACCTGCAG_CCTTCAGGCATGCCGTGACCTC	Deletion of *phzE*
JJC-PCP-Bz-phzE-D1_Rv	GATGATGCGCGGACCTTCCTGGG_GGGAACCGGTTGCAGGATGCGTTC	
JJC-PCP-Bz-phzE-D2_Fw	ATGGAACGCATCCTGCAACCGGTTCCC_CCCAGGAAGGTCCGCGCATCAT	
JJC-PCP-Bz-phzE-D2_Rv	TCCCGGGAGAGCTCAGATCT_CCCAATAGCGGTTTGCCTTGTATTG	
JJC-PCP-Bz-pbzB-SUMO_Fw	CATTGAGGCCCATCGTGAACAGATTGGTGGT_ACAGTCAATCATCAACCGCTT	Expression of *pbzB*
JJC-PCP-Bz-pbzB-SUMO_Rv	CTTTCGGGCTTTGTTAGCAGCCGGATCCTTA_TCAGATTGCCAGGTAGTCAG	

DNA sequence of PbzB T68Y H78Y (mutations are indicated in red)

CATTGAGGCCCATCGTGAACAGATTGGTGGTACAGTCAATCATCAACCGCTTCTTACCCAGGCCGATCTGCTCAAGCG

TGGCCTGGCCGATCTTCAGGAACATGATGCCGAACTGGCGCGGATCCTCGACGCCGAGGTCGCTCGCCAGCAACGC

ACCCTCTCGCTGGTCGCCTCTTGCTGCGCGGTCAAGCCCCGCACCCTGGCGGCTTCGTCCTCCGCGCTGGTCAATG

TC**TAT**GCCGAAGGTGTTCCCGGACGCCGCTAT**TAT**GCCGGCTGCGAGAACGTGGACCTGGTCGAGTCCCTGGCGAT

CCAGCGTGCCCGGGAGCTGTTCGGTGCGCAATATGCCGGTGTCCAGTCGCACTCGGCCTCCAGCGCCAACTACCAG

GTGCTCGCCGCCCTGCTCGAACCAGGCGACACGCTACTGGGCATGGCCCTGGATAACGGTGGCCACCTGACCCATG

GCAGCCCCGTGACCTTCTCGGGCACTTACTACAAGGCCATCGGCTATGGCACCACCAAGGAAGGCCTGATCGACTAC
GACGAAGTCCGCAGGCTGGCCCTGGAGCATCGGCCACGACTGATCATCTGCGGTGCCACGGCCTACTCCAGGGTCG
TGGACTTCGAGCGCTTCCGGCAGATCGCCGATGAAGCCGGTGCCATCCTGATGGCCGACATCTCGCATATCGCCGGC
CTGGTGGCGACCGGGCGTCATCCGAGCCCGATCGACGCCGCGCACGTCACCACCACCTGCACCCACAAGCAGCTC
GTAGGCCCCCGTGGCGGCCTGATTCTCTCGGGCCGTGACGCCAATGAAAAAGTCCCCGGCCGCGATGCGACCTTCA
GCCGGGTGCTCGAACTGGCGGTGTTCCCCAGGATGCAAGGCGCGCCGGCCGTCAACATGATGGCCGCCAAGGCCG
CAGCACTGGGTTACGCCATGACCCCGGAATTCGATGCCGAGATGCAACGCATCCGCGACGCGGCCGATGTGATGGC
CAGCGAATTCCAGGCCCGGGACTATGAGGTGGTAGGCGGTCGCAGCGAAAACCACACCATCCTGATTCGCTTGCGT
GCCGCGATGACCGGCGCTATCGCCGAGACCGCGCTGGAGCACTGCGGAATCGTCGTCAACAAGAACCGTGTGCCC
GGCGAAACACGTTCGTCCTTCGTCACCAGCGGCCTGCGCATCGGTACCGGCGCCCTCGCCCAGCGCCATGTCGACG
CGCAGGGATGCCGGCAGATCGTCGACCTGCTCTGCCGGATCCTGGACGAGGTGACTCCGCTCGGCGAGAGCGAGT
TCACCCTGGACCCGGCCCTGCGCAAGCAATTCTGTGCGGAAGCCGAGGCGCTGTGCGTGAAGTATCCGATCGCTGA
CTACCTGGCAATCTGATAAGGATCCGGCTGCTAACAAAGCCCGAAAG

Table S4. Putative functional assignments of biosynthetic genes in this study.

BGC	Protein	Homolog (accession number, organism)	Identified/Proposed function	Coverage/ Identity (%)
xsb				
	XsbA	ScgG (AAL06666.1, *Streptomyces globisporus*)	Fe-S flavin-dependent oxidoreductase	87/44
	XsbB	MchC (APZ78729.1, *Myxococcus* sp. 171)	NRPS	71/26
	XsbC	ScgD5 (AAL06665.1, *Streptomyces globisporus*)	acyl AMP-ligase	98/39
	XsbD	XpzS (WP_038233770.1, *Xenorhabdus szentirmaii* DSM 16338)	3-oxoacyl-ACP synthase	99/83
	XsbE	MenF (ACD75091.1, *Enterobacter cloacae*)	isochorismate synthase	94/47
xpz				
	XpzC	PhzE (AAC64488.1, *Pseudomonas aeruginosa* PAO1)	ADIC synthase	98/43
pbz				
	PbzA	ObaI (AQZ26587.1, *Pseudomonas fluorescens*)	NRPS	92/30
	PbzB	GlyA (WP_078857614.1, *Streptomyces* sp. NRRL F-4474)	serine hydroxymethyltransferase	95/53
	PbzC	ScgG (AAL06666.1, *Streptomyces globisporus*)	Fe-S flavin-dependent oxidoreductase	81/47
	PbzD	Plu3263 (CAE15637.1, *Photorhabdus laumondii subsp. laumondii* TTO1)	NRPS	91/26
	PbzE	ScgD5 (AAL06665.1, *Streptomyces globisporus*)	acyl AMP-ligase	99/43
	PbzF	ScgD (AAL06664.1, *Streptomyces globisporus*)	anthranilate synthase component I	90/36
	PbzG	ScgD1 (AAL06663.1, *Streptomyces globisporus*)	anthranilate synthase component II	95/44
	PbzH	FmoK (BAP16698.1, *Streptomyces* sp. Sp080513GE-23)	transmembrane transporter	100/38
	PbzI	FmoA4 (BAP16697.1, *Streptomyces* sp. Sp080513GE-23)	NRPS	86/23
	PbzJ	(WP_041482455.1, *Bacillus velezensis*)	hypothetical protein	99/52
	PbzK	(WP_101563111.1, *Bacillus velezensis*)	winged helix DNA-binding domain-containing protein	99/58
phz				
	PhzE	(AAC64488.1, *Pseudomonas aeruginosa* PAO1)	ADIC synthase	97/75

Table S5. HR-ESI-MS data of all compounds described in this work.

Compound	Detected mass	Calculated mass	Error (ppm)	Ion formula
benzoxazolinate (**1**)	206.0446 $[M + H]^+$	206.0448 $[M + H]^+$	0.9	$C_{10}H_8NO_4$ $[M + H]^+$
benzobactin A (**2**)	422.1192 $[M – H_2O + H]^+$	422.1194 $[M – H_2O + H]^+$	0.5	$C_{18}H_{20}N_3O_9$ $[M – H_2O + H]^+$
benzobactin B (**3**)	305.0767 $[M – H_2O + H]^+$	305.0768 $[M – H_2O + H]^+$	0.4	$C_{14}H_{13}N_2O_6$ $[M – H_2O + H]^+$
benzobactin C (**4**)	609.1467 $[M – H_2O + H]^+$	609.1464 $[M – H_2O + H]^+$	-0.6	$C_{28}H_{25}N_4O_{12}$ $[M – H_2O + H]^+$
benzobactin-628	628.1666 $[M – H_2O + H]^+$	628.16474 $[M – H_2O + H]^+$	-3.0	$C_{28}H_{28}N_4O_{13}$ $[M – H_2O + H]^+$
benzobactin-1012a (R_t 8.0 min)	1012.2412 $[M + H]^+$	1012.2447 $[M + H]^+$	3.5	$C_{56}H_{36}N_8O_{12}$ $[M + H]^+$
benzobactin-1012b (R_t 8.5 min)	1012.2421 $[M + H]^+$	1012.2447 $[M + H]^+$	2.6	$C_{56}H_{36}N_8O_{12}$ $[M + H]^+$
2-hydroxylmethylserine	136.0604 $[M + H]^+$	136.0604 $[M + H]^+$	0.3	$C_4H_{10}NO_4$ $[M + H]^+$

Table S6. ^{1}H (500 MHz) and ^{13}C (125 MHz) NMR data assignments for benzoxazolinate (**1**) and benzobactin B (**3**) in DMSO-d_6 (for NMR spectra see Figures S15–26).

	No.	benzoxazolinate		benzobactin B	
		δ_H (mult., J)	δ_C, mult.	δ_H (mult., J)	δ_C, mult.
benzoxazolinate					
	2	-	148.2, C	-	148.1, C
	3	-	155.3, C	-	155.5, C
	4	11.35 (s)	-	11.64 (s)	-
	5	-	127.7, C	-	126.4, C
	6	-	111.2, CH	-	111.5, C
	7	7.63 (dd, 7.9, 1.0)	125.3, CH	7.47 (dd, 8.0, 1.0)	123.3, CH
	8	7.06 (t, 8.0)	122.8, CH	7.12 (t. 8.0)	123.2, CH
	9	7.28 (br d, 8.0)	119.5, CH	7.29 (br d, 7.4)	119.1, CH
	10	-	141.5, C	-	141.5, C
	11	5.46 (d, 1.6)<br>5.12 (br s)	98.7, CH_2	5.49 (d. 1.7)<br>5.15 (br s)	99.0, CH_2
	12	-	169.1, C	-	163.2, C
2-hydroxymethylserine					
	13	-	-	4.65 (d, 8.5)<br>4.44 (d, 8.5)	71.3, CH_2
	14	-	-	3.75 (d, 11.0)<br>3.72 (d, 11.0)	64.8, CH_2
	15	-	-	-	79.7, C
	16	-	-	-	172.8, C

Table S7. Structure data for PbzB.

	PbzB (PDB: 7QWZ)
Data collection	
Space group	C121
Cell dimensions	
a, *b*, *c* (Å)	132.4<br>59.64<br>112.12
a, b, g (°)	90<br>109.409<br>90
Wavelength (Å)	0.976254
Resolution (Å)	47.32 - 2.811 (2.912 - 2.811)
R_{merge}	0.1694 (1.057)
I / s*I*	8.67 (1.77)
Completeness (%)	99.61 (98.47)
Redundancy	4.9 (5.0)
CC1/2	0.994 (0.764)
Refinement	
Resolution (Å)	47.32 - 2.811
No. reflections (total)	20299 (1991)
R_{work} / R_{free}	0.235/0.294
No. atoms	6019
Protein	6019
Ligand/ion	0
Water	0
B-factors	75.79
Protein	75.79
Ligand/ion	0
Water	0
R.m.s. deviations	
Bond lengths (Å)	0.006
Bond angles (°)	1.15
Ramachandran	
Favored (%)	96.30
Allowed (%)	3.32
Outliers (%)	0.38

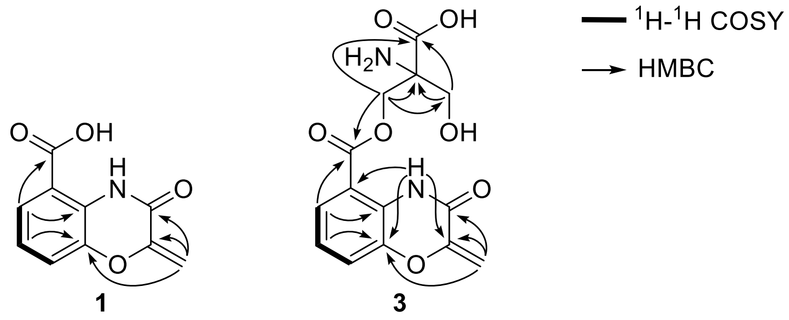

Figure S1. 2D NMR correlations of benzoxazolinate (**1**) and benzobactin B (**3**).

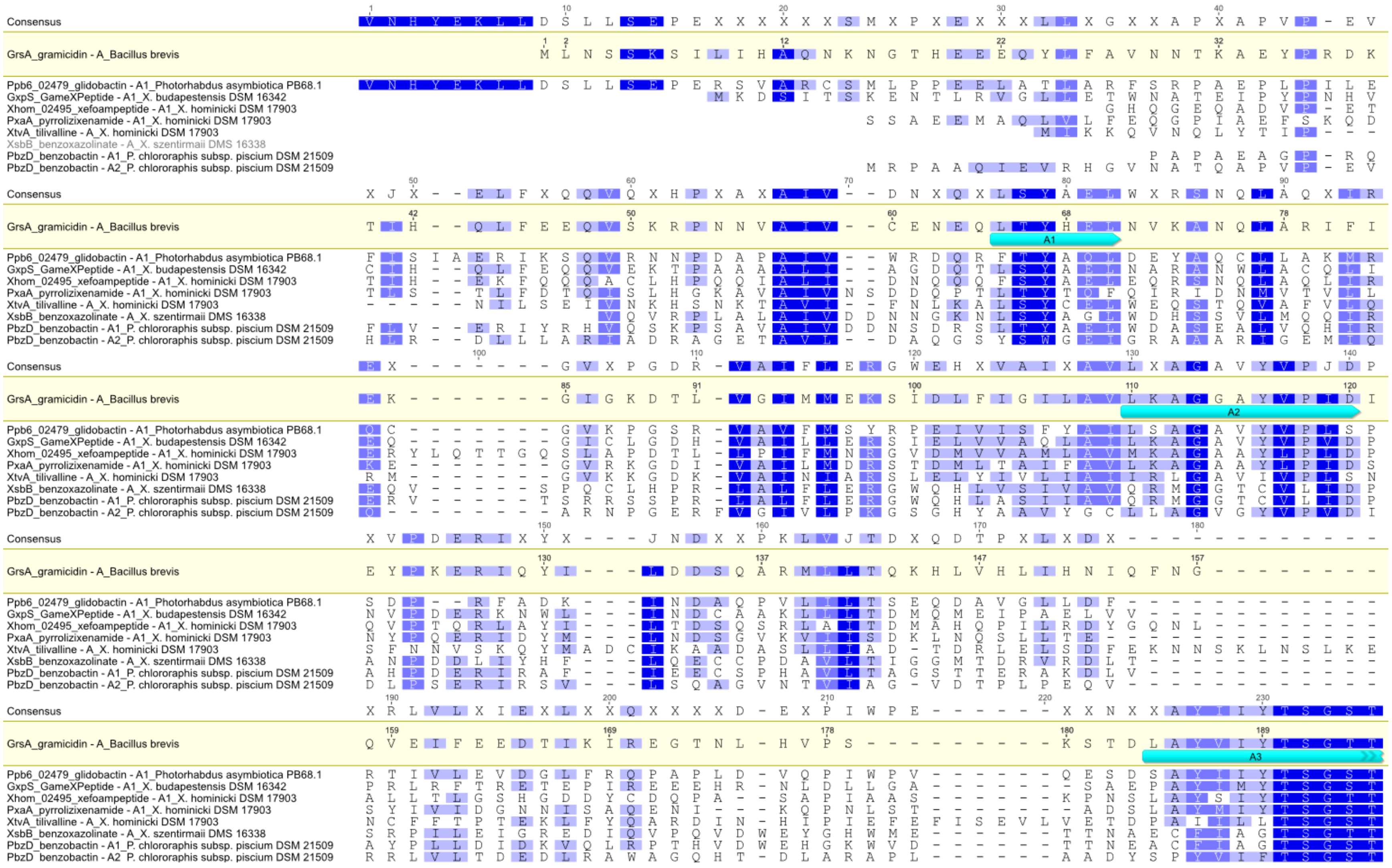
Consensus
GrsA_gramicidin - A_Bacillus brevis
Ppb6_02479_glidobactin - A1_Photorhabdus asymbiotica PB68.1
GxpS_GameXPeptide - A1_X. budapestensis DSM 16342
Xhom_02495_xefoampeptide - A1_X. hominicki DSM 17903
PxaA_pyrrolizixenamide - A1_X. hominicki DSM 17903
XtvA_tilivalline - A_X. hominicki DSM 17903
XsbB_benzoxazolinate - A_X. szentirmaii DMS 16338
PbzD_benzobactin - A1_P. chlororaphis subsp. piscium DSM 21509
PbzD_benzobactin - A2_P. chlororaphis subsp. piscium DSM 21509
A1
A2
A3

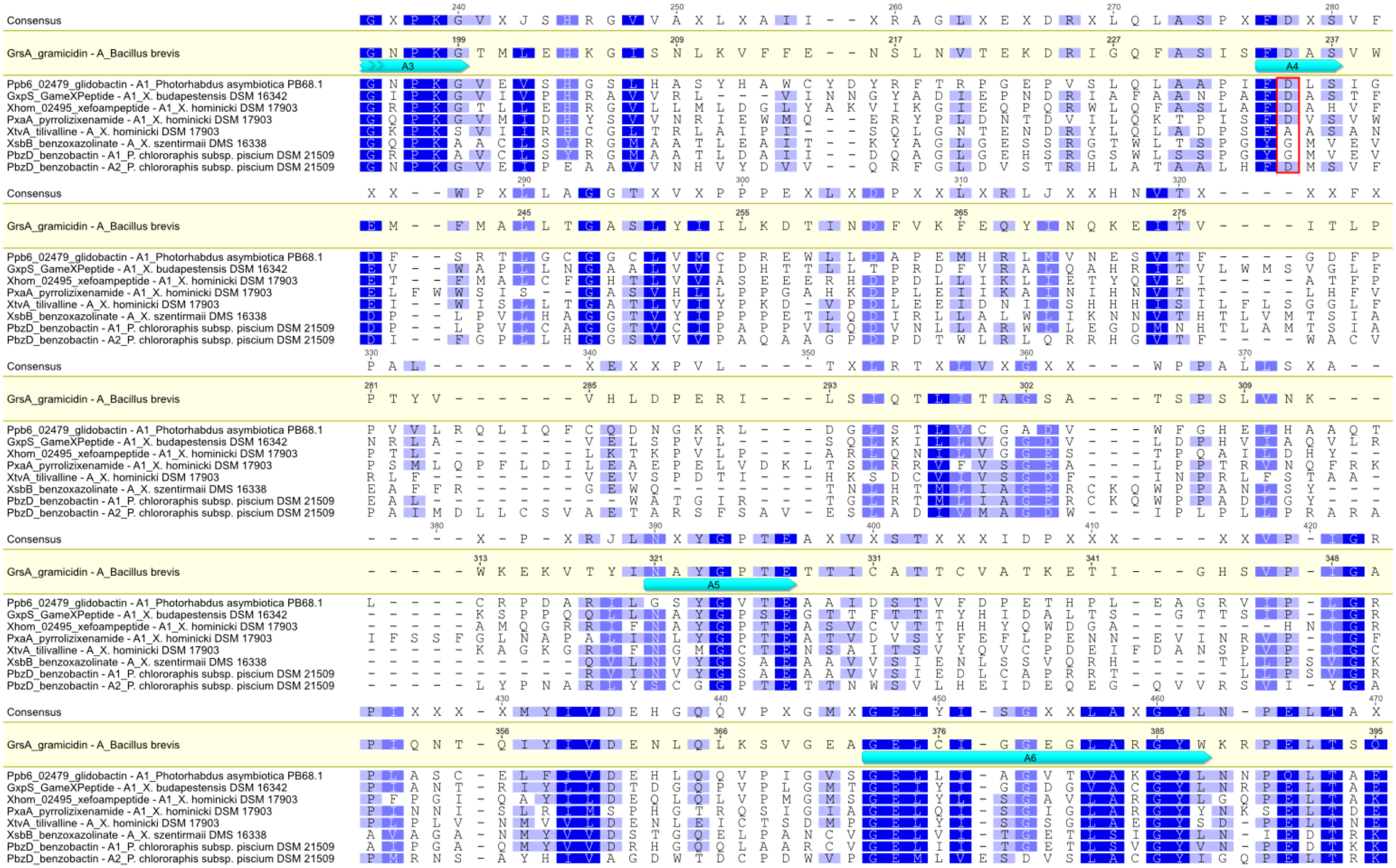

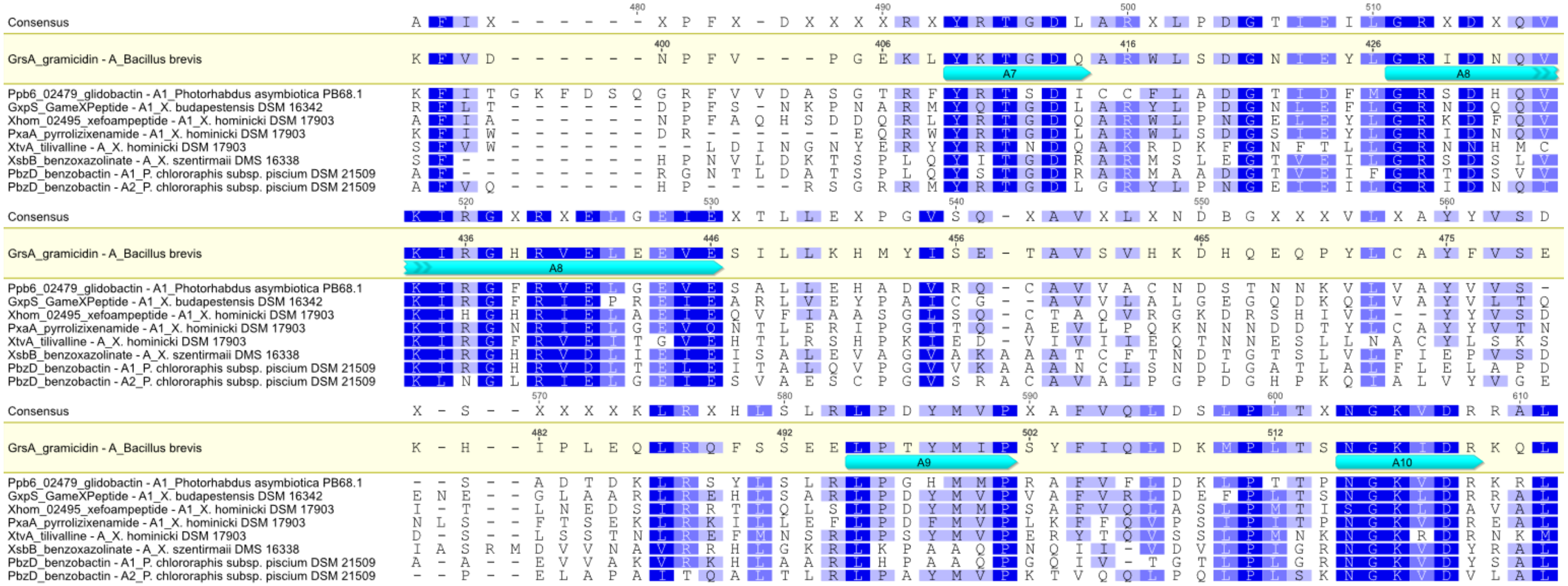

Figure S2. Multiple-sequence alignment of XsbA-A, PbzD-A_1, and PbzD-A_2 with biochemically characterized A domains by Cluster Omega Alignment. Conserved core motifs A1-A10 (ref[25]) are highlighted with arrows and GrsA gramicidin was set as a reference sequence. The A4 motifs of XsbA-A and PbzD-A_1 lack the conserved aspartate (red rectangle) that typically interacts with the α-NH_2 of amino acid substrates. The A domain of tilivalline activates 3-hydroxyanthranilic acid.[26] GrsA (PDB 1AMU) is set as a reference sequence.

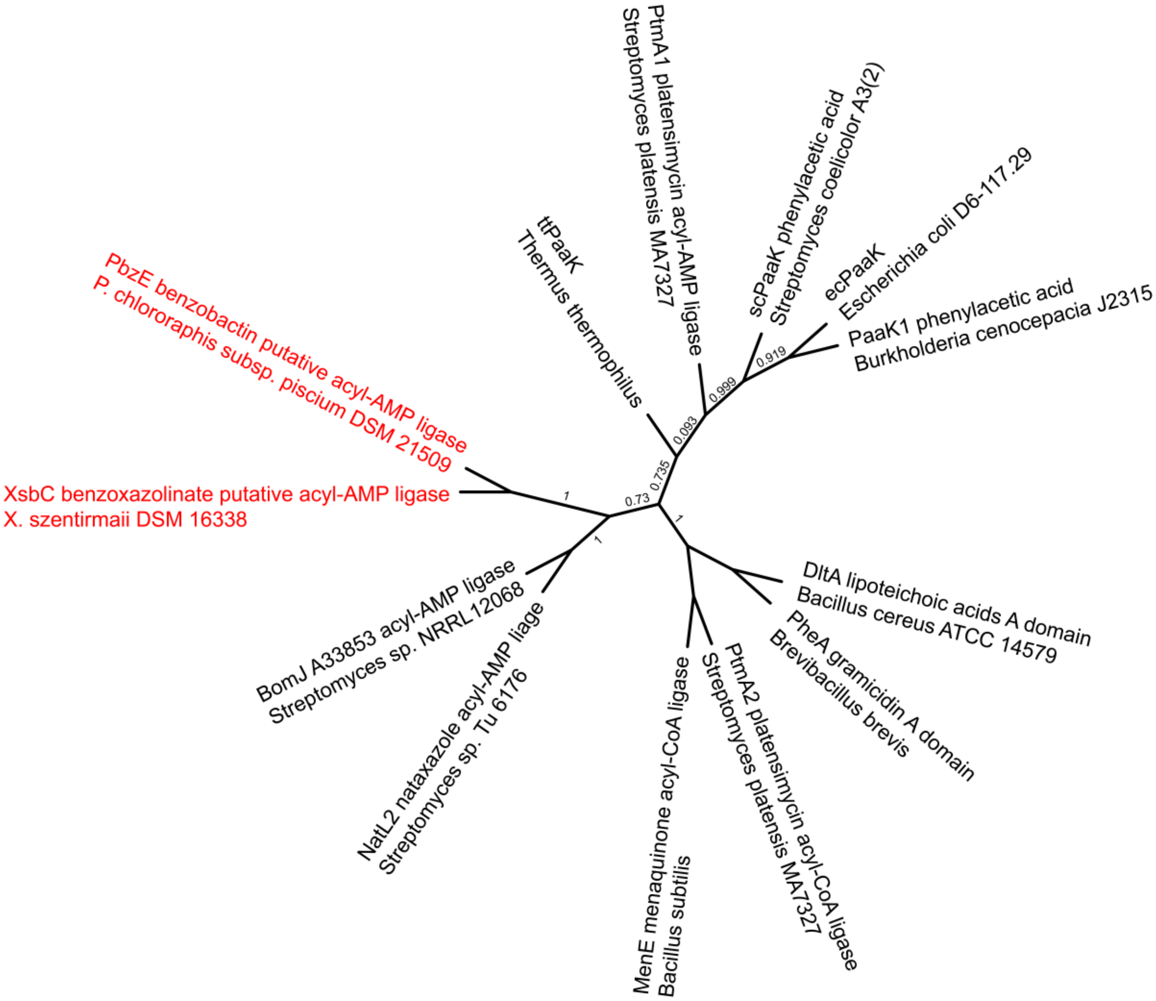

Figure S3. Phylogenetic analysis of XsbC and PbzE with representative members of the ANL superfamily by FastTree2.1.11 (ref[27, 28]). XsbC and PbzE are closely related to NatL2 and BomJ. MenE (PDB ID: 5BUQ), scPaaK (NCBI: accessionWP_011031681.1), ttPaaK (NCBI: WP_172596917.1), and ecPaak (NCBI: CDP76889.1). XsbC and PbzE are highlighted in red.

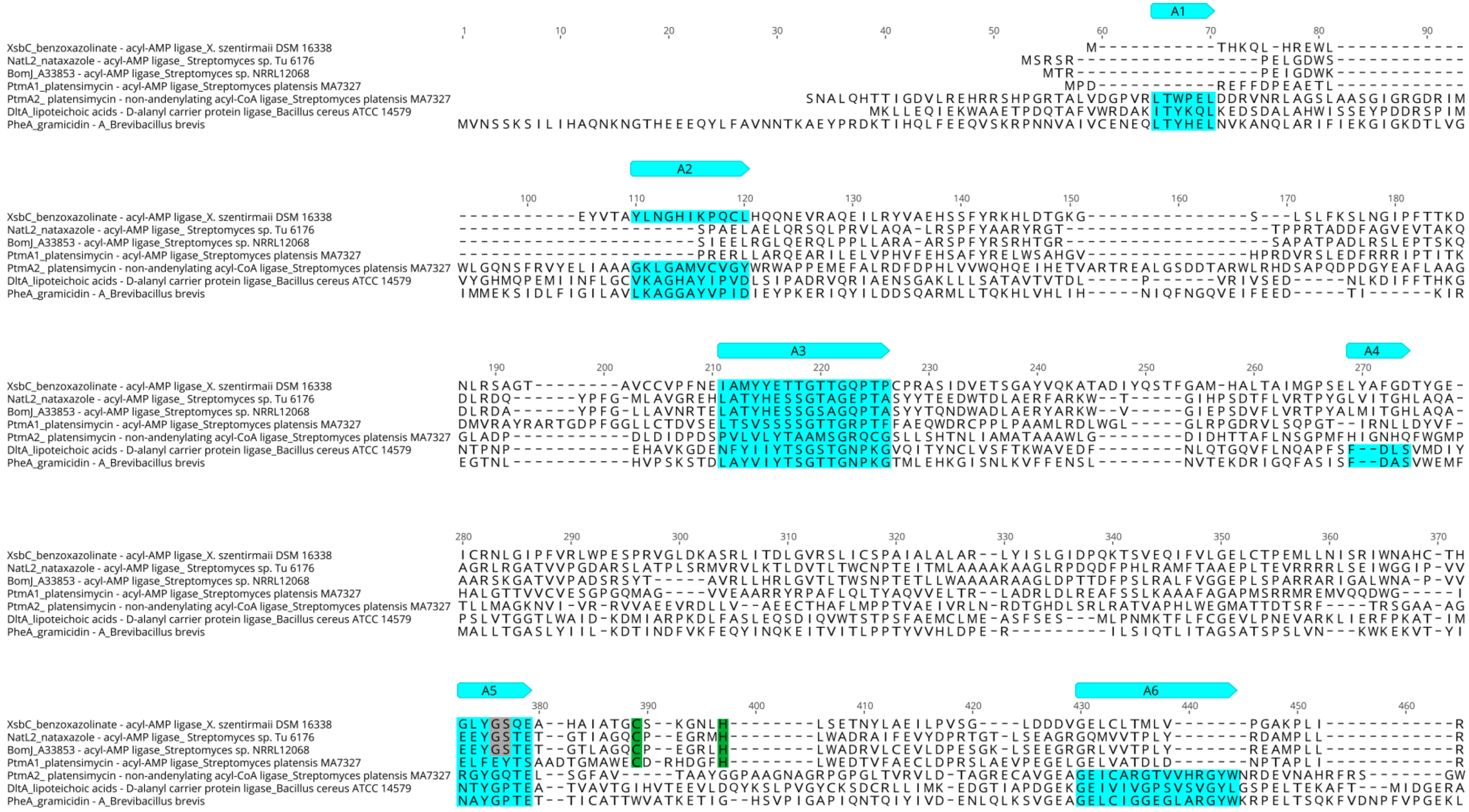
A1
1 10 20 30 40 50 60 70 80 90
XsbC_benzoxazolinate - acyl-AMP ligase_X. szentirmaii DSM 16338 M-----------THKQL-HREWL------------
NatL2_nataxazole - acyl-AMP ligase_ Streptomyces sp. Tu 6176 MSRSR-----------------PELGDWS------------
BomJ_A33853 - acyl-AMP ligase_Streptomyces sp. NRRL12068 MTR-----------------PEIGDWK------------
PtmA1_platensimycin - acyl-AMP ligase_Streptomyces platensis MA7327 MPD-----------REFFDPEAETL------------
PtmA2_ platensimycin - non-andenylating acyl-CoA ligase_Streptomyces platensis MA7327 SNALQHTTIGDVLREHRRSHPGRTALVDGPVRLTWPELDDRVNRLAGSLAASGIGRGDRIM
DltA_lipoteichoic acids - D-alanyl carrier protein ligase_Bacillus cereus ATCC 14579 MKLLEQIEKWAAETPDQTAFVWRDAKITYKQLKEDSDALAHWISSEYPDDRSPIM
PheA_gramicidin - A_Brevibacillus brevis MVNSSKSILIHAQNKNGTHEEEQYLFAVNNTKAEYPRDKTIHQLFEEQVSKRPNNVAIVCENEQLTYHELNVKANQLARIFIEKGIGKDTLVG
A2
100 110 120 130 140 150 160 170 180
XsbC_benzoxazolinate - acyl-AMP ligase_X. szentirmaii DSM 16338 -----------EYVTAYLNGHIKPQCLHQQNEVRAQEILRYVAEHSSFYRKHLDTGKG---------------S---LSLFKSLNGIPFTTKD
NatL2_nataxazole - acyl-AMP ligase_ Streptomyces sp. Tu 6176 ----------------------SPAELAELQRSQLPRVLAQA-LRSPFYAARYRGT------------------TPPRTADDFAGVEVTAKQ
BomJ_A33853 - acyl-AMP ligase_Streptomyces sp. NRRL12068 ----------------------SIEELRGLQERQLPPLLARA-ARSPFYRSRHTGR------------------SAPATPADLRSLEPTSKQ
PtmA1_platensimycin - acyl-AMP ligase_Streptomyces platensis MA7327 ----------------------PRERLLARQEARILELVPHVFEHSAFYRELWSAHGV---------------HPRDVRSLEDFRRRIPTITK
PtmA2_ platensimycin - non-andenylating acyl-CoA ligase_Streptomyces platensis MA7327 WLGQNSFRVYELIAAAGKLGAMVCVGYWRWAPPEMEFALRDFDPHLVVWQHQEIHETVARTREALGSDDTARWLRHDSAPQDPDGYEAFLAAG
DltA_lipoteichoic acids - D-alanyl carrier protein ligase_Bacillus cereus ATCC 14579 VYGHMQPEMIINFLGCVKAGHAYIPVDLSIPADRVQRIAENSGAKLLLSATAVTVTDL-----P------VRIVSED-----NLKDIFFTHKG
PheA_gramicidin - A_Brevibacillus brevis IMMEKSIDLFIGILAVLKAGGAYVPIDIEYPKERIQYILDDSQARMLLTQKHLVHLIH-----NIQFNGQVEIFEED-----TI------KIR
A3 A4
190 200 210 220 230 240 250 260 270
XsbC_benzoxazolinate - acyl-AMP ligase_X. szentirmaii DSM 16338 NLRSAGT--------AVCCVPFNEIAMYYETTGTTGQPTPCPRASIDVETSGAYVQKATADIYQSTFGAM-HALTAIMGPSELYAFGDTYGE-
NatL2_nataxazole - acyl-AMP ligase_ Streptomyces sp. Tu 6176 DLRDQ------YPFG-MLAVGREHLATYHESSGTAGEPTASYYTEEDWTDLAERFARKW--T-----GIHPSDTFLVRTPYGLVITGHLAQA-
BomJ_A33853 - acyl-AMP ligase_Streptomyces sp. NRRL12068 DLRDA------YPFG-LLAVNRTELATYHESSGSAGQPTASYYTQNDWADLAERYARKW--V-----GIEPSDVFLVRTPYALMITGHLAQA-
PtmA1_platensimycin - acyl-AMP ligase_Streptomyces platensis MA7327 DMVRAYRARTGDPFGGLLCTDVSELTSVSSSSGTTGRPTFFAEQWDRCPPLPAAMLRDLWGL-----GLRPGDRVLSQPGT--IRNLLDYVF-
PtmA2_ platensimycin - non-andenylating acyl-CoA ligase_Streptomyces platensis MA7327 GLADP-----------DLDIDPDSPVLVLYTAAMSGRQCGSLLSHTNLIAMATAAAWLG--------DIDHTTAFLNSGPMFHIGNHQFWGMP
DltA_lipoteichoic acids - D-alanyl carrier protein ligase_Bacillus cereus ATCC 14579 NTPNP-----------EHAVKGDENFYIIYTSGSTGNPKGVQITYNCLVSFTKWAVEDF--------NLQTGQVFLNQAPFSF--DLSVMDIY
PheA_gramicidin - A_Brevibacillus brevis EGTNL-----------HVPSKSTDLAYVIYTSGTTGNPKGTMLEHKGISNLKVFFENSL--------NVTEKDRIGQFASISF--DASVWEMF
280 290 300 310 320 330 340 350 360 370
XsbC_benzoxazolinate - acyl-AMP ligase_X. szentirmaii DSM 16338 ICRNLGIPFVRLWPESPRVGLDKASRLITDLGVRSLICSPAIALALAR--LYISLGIDPQKTSVEQIFVLGELCTPEMLLNISRIWNAHC-TH
NatL2_nataxazole - acyl-AMP ligase_ Streptomyces sp. Tu 6176 AGRLRGATVVPGDARSLATPLSRMVRVLKTLDVTLTWCNPTEITMLAAAAKAAGLRPDQDFPHLRAMFTAAEPLTEVRRRRLSEIWGGIP-VV
BomJ_A33853 - acyl-AMP ligase_Streptomyces sp. NRRL12068 AARSKGATVVPADSRSYT-----AVRLLHRLGVTLTWSNPTETLLWAAAARAAGLDPTTDFPSLRALFVGGEPLSPARRARIGALWNA-P-VV
PtmA1_platensimycin - acyl-AMP ligase_Streptomyces platensis MA7327 HALGTTVVCVESGPGQMAG----VVEAARRYRPAFLQLTYAQVVELTR--LADRLDLREAFSSLKAAAFAGAPMSRRMREMVQQDWG-----I
PtmA2_ platensimycin - non-andenylating acyl-CoA ligase_Streptomyces platensis MA7327 TLLMAGKNVI-VR-RVVAEEVRDLLV--AEECTHAFLMPPTVAEIVRLN-RDTGHDLSRLRATVAPHLWEGMATTDTSRF----TRSGAA-AG
DltA_lipoteichoic acids - D-alanyl carrier protein ligase_Bacillus cereus ATCC 14579 PSLVTGGTLWAID-KDMIARPKDLFASLEQSDIQVWTSTPSFAEMCLME-ASFSES---MLPNMKTFLFCGEVLPNEVARKLIERFPKAT-IM
PheA_gramicidin - A_Brevibacillus brevis MALLTGASLYIIL-KDTINDFVKFEQYINQKEITVITLPPTYVVHLDPE-R---------ILSIQTLITAGSATSPSLVN---KWKEKVT-YI
A5 A6
380 390 400 410 420 430 440 450 460
XsbC_benzoxazolinate - acyl-AMP ligase_X. szentirmaii DSM 16338 GLYGSQEA--HAIATGCS--KGNLH--------LSETNYLAEILPVSG----LDDDVGELCLTMLV-------PGAKPLI------------R
NatL2_nataxazole - acyl-AMP ligase_ Streptomyces sp. Tu 6176 EEYGSTET--GTIAGQCP--EGRMH--------LWADRAIFEVYDPRTGT-LSEAGRGQMVVTPLY-------RDAMPLL------------R
BomJ_A33853 - acyl-AMP ligase_Streptomyces sp. NRRL12068 EEYGSTET--GTLAGQCP--EGRLH--------LWADRVLCEVLDPESGK-LSEEGRGRLVVTPLY-------REAMPLL------------R
PtmA1_platensimycin - acyl-AMP ligase_Streptomyces platensis MA7327 ELFEYTSAADTGMAWECD-RHDGFH--------LWEDTVFAECLDPRSLAEVPEGELGELVATDLD-------NPTAPLI------------R
PtmA2_ platensimycin - non-andenylating acyl-CoA ligase_Streptomyces platensis MA7327 RGYGQTEL--SGFAV-----TAAYGGPAAGNAGRPGPGLTVRVLD-TAGRECAVGEAGEICARGTVVHRGYWNRDEVNAHRFRS-------GW
DltA_lipoteichoic acids - D-alanyl carrier protein ligase_Bacillus cereus ATCC 14579 NTYGPTEA--TVAVTGIHVTEEVLDQYKSLPVGYCKSDCRLLIMK-EDGTIAPDGEKGEIVIVGPSVSVGYLGSPELTEKAFT---MIDGERA
PheA_gramicidin - A_Brevibacillus brevis NAYGPTET--TICATTWVATKETIG--HSVPIGAPIQNTQIYIVD-ENLQLKSVGEAGELCIGGEGLARGYWKRPELTSQKFVDNPFVPGEKL

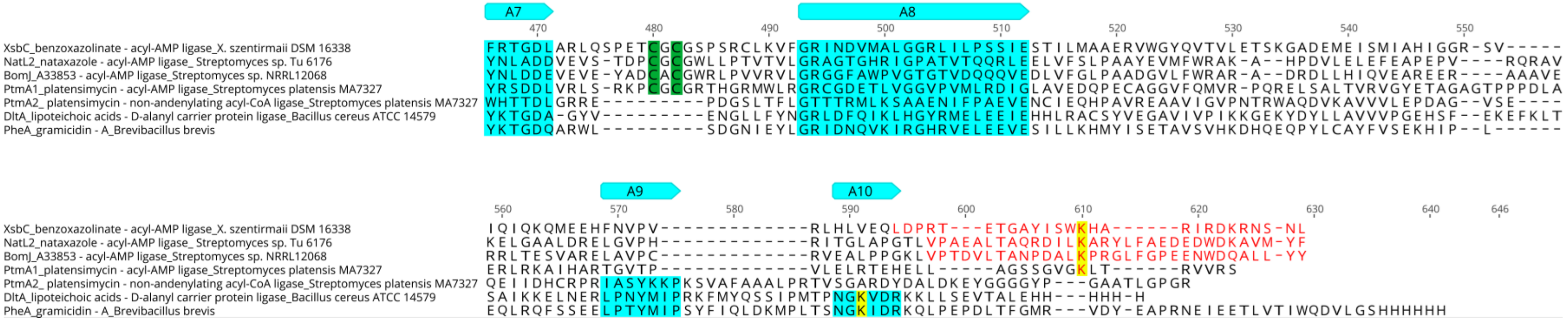

Figure S4. Multiple-sequence alignment of XsbC with biochemically characterized acyl-AMP ligases (NatL2, BomJ, and PtmA1), acyl-CoA ligase (PtmA2), and A domains (DltA and PheA) by Clustal Omega Alignment. Adenylation domain core motifs A1–A10 (turquoise),[25] the catalytic lysine residue (yellow), the Michaelis complex-forming amino acids of the first half (adenylation) reaction (grey), the zinc-binding motif (green), and the characteristic C-terminal extensions (red).

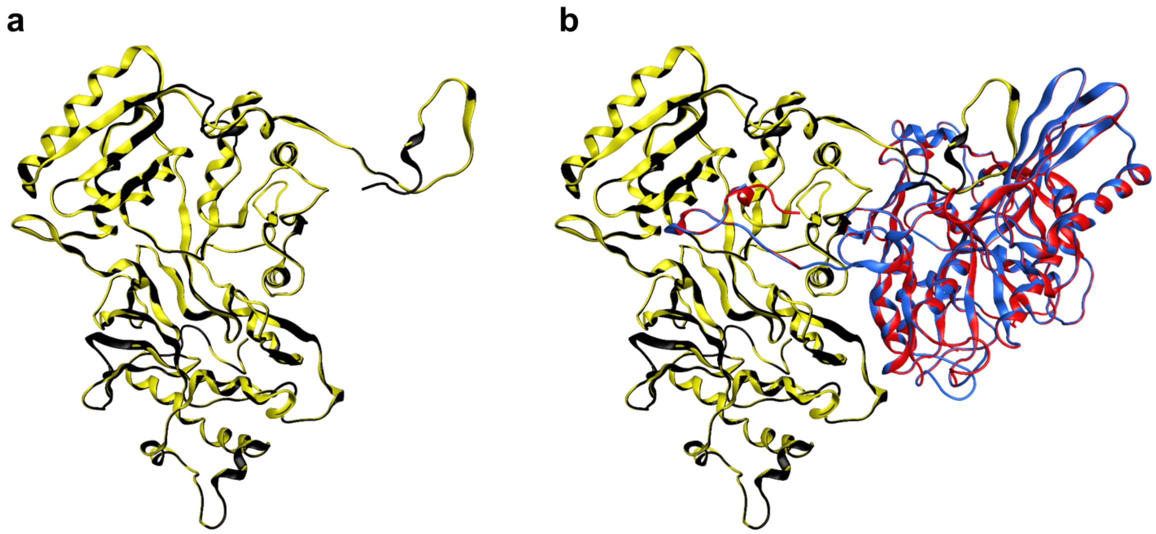

Figure S5. Overlap of a homology model of XsbC with the crystal structure of NatL2 (6SIY). (a) Superimposed monomers of NatL2 (black) and XsbC (yellow) with an RMSD of 0.6 Å over 441 Cα atoms. (b) Superimposed dimers of NatL2 (chain A, black; chain B, red) and XsbC (chain A, yellow; chain B, blue) with an RMSD of 0.8 Å over 882 Cα atoms.

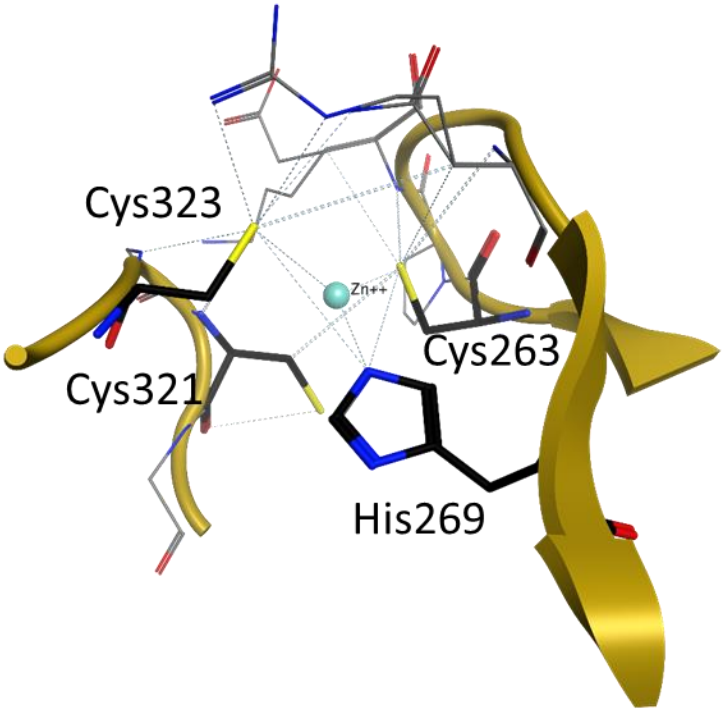

Figure S6. Zinc binding motif in XsbC. A tetrahedrally coordinated (Cys263, His269, Cys321, and Cys323) zinc (Zn^{2+}) is found in the N-terminal domain over 20 Å away from the AMP. Zinc binding and the associated introduced structural rigidity might play a key role in preventing the formation of the second active conformation, involving rotation of the small C-terminal domain relative to the large N-terminal domain by a large angle, as much as 140° as observed for bacterial acetyl-CoA synthetases[29] and 4-chlorobenzoate CoA ligase (4CBL)[30].

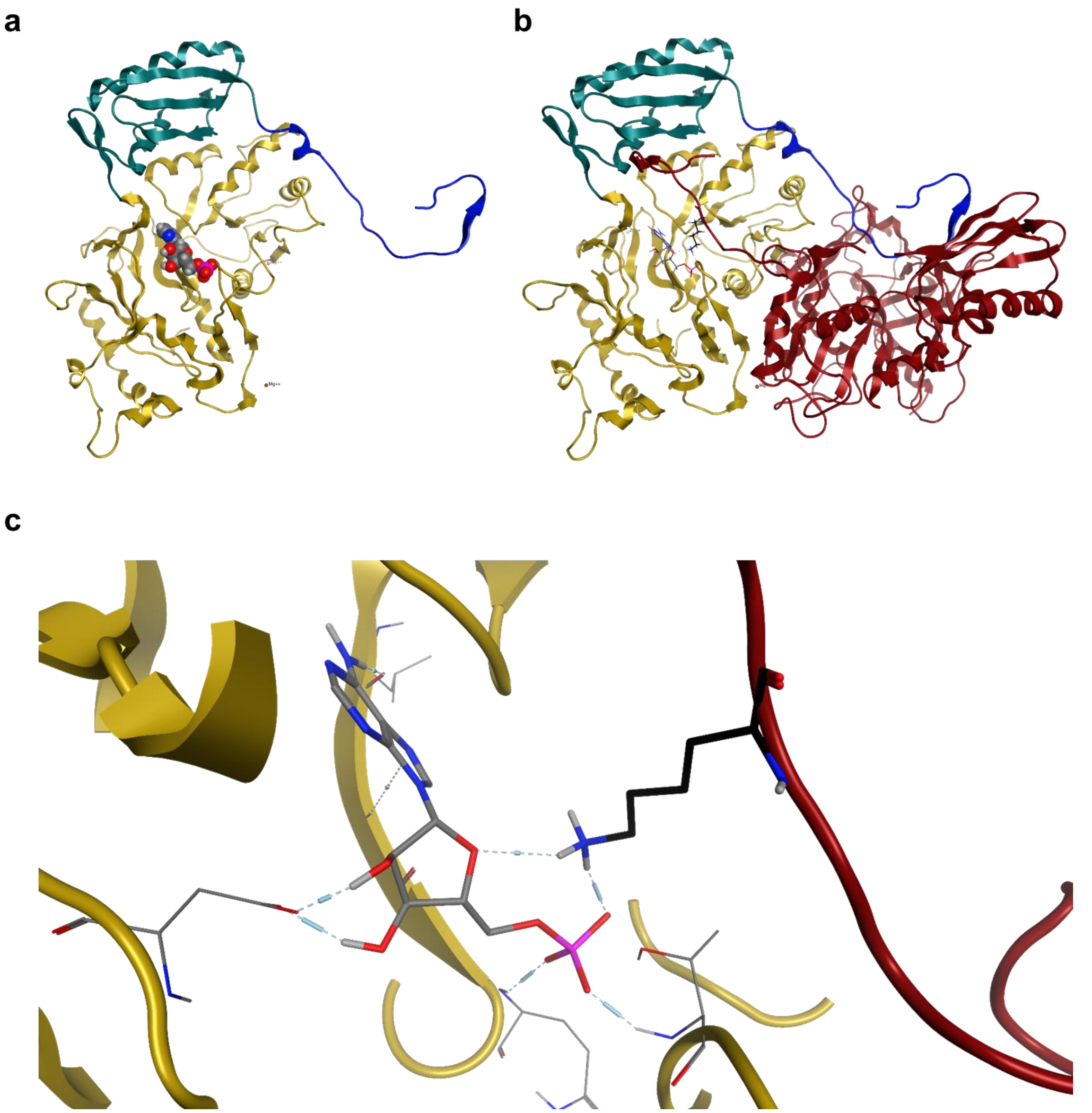

Figure S7. Overall structure and binding site of modeled XsbC. The XsbC N-terminal, C-terminal, and C-terminal extensions are colored yellow, green, and blue, respectively. (a) XsbC monomer (chain A). AMP is shown as spacefill (van-der-Waals surface). (b) XsbC dimer. The second monomer is colored red (chain B). Also depicted are AMP (stick representation) bound within the active site of chain A, and the invariant Lys429 (black stick representation) from the other monomer (chain B). (c) Active site of XsbC chain A. Lys429 (black) in the C-terminal extension from the other monomer (chain B, red) is forming a salt bridge with a bound AMP.

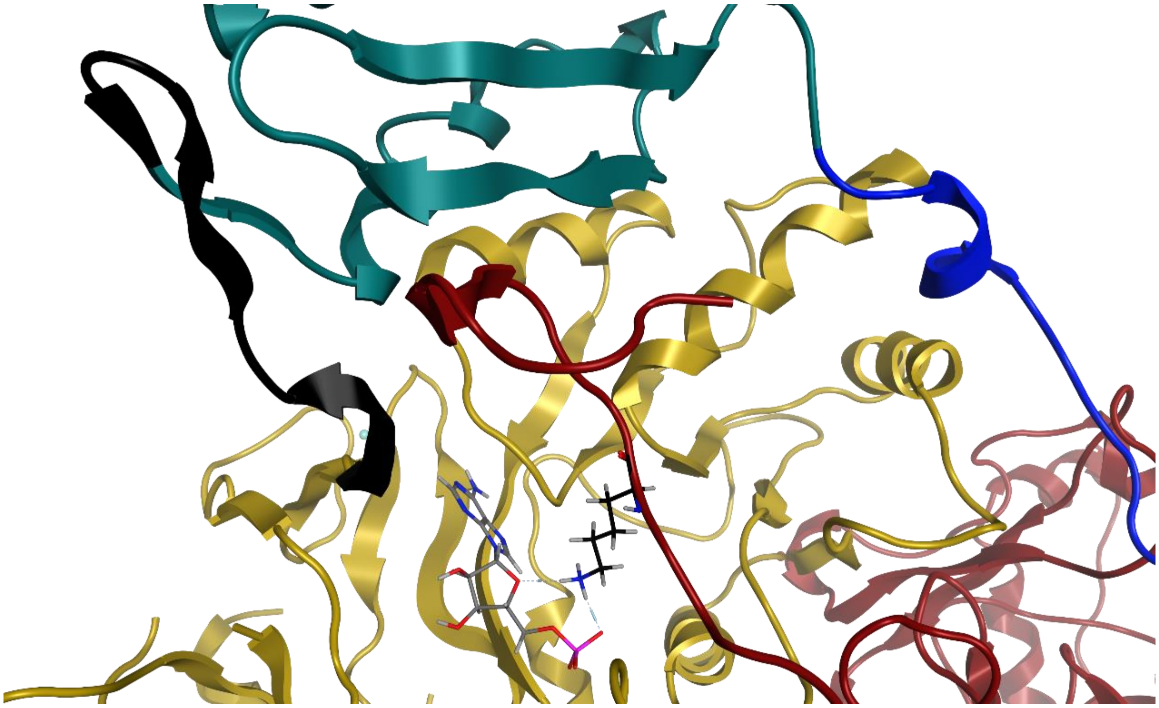

Figure S8. XsbC A8 hinge region. The conserved A domain core motif A8 of chain A (black) is known to serve as a hinge between the C- (yellow) and N-terminal (green) domains. This hinge region enables the catalysis of CoA thioester formation. Whereas fatty acyl-AMP ligases (FAALs) are locked in the ‘closed’ arrangement and are unable to catalyze CoA thioester formation due to insertion into the hinge. NatL2-like enzymes might be locked in the ‘closed’ conformation, or rather prevent access of CoA by the C-terminal extension shielding the catalytic center.

a

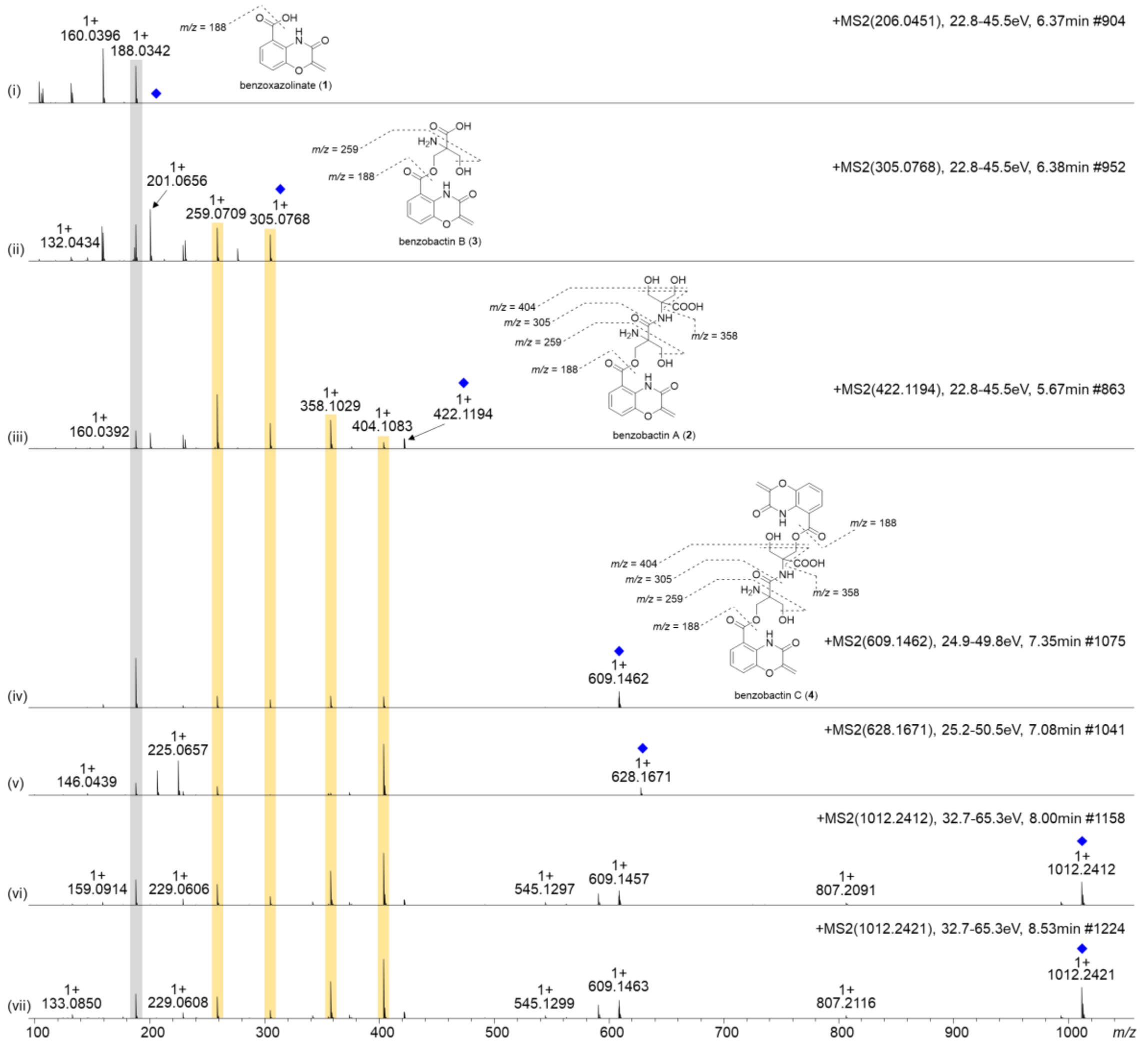
+MS2(206.0451), 22.8-45.5eV, 6.37min #904
+MS2(305.0768), 22.8-45.5eV, 6.38min #952
+MS2(422.1194), 22.8-45.5eV, 5.67min #863
+MS2(609.1462), 24.9-49.8eV, 7.35min #1075
+MS2(628.1671), 25.2-50.5eV, 7.08min #1041
+MS2(1012.2412), 32.7-65.3eV, 8.00min #1158
+MS2(1012.2421), 32.7-65.3eV, 8.53min #1224
benzoxazolinate (1)
benzobactin B (3)
benzobactin A (2)
benzobactin C (4)
m/z = 188
m/z = 259
m/z = 305
m/z = 358
m/z = 404
(i)
(ii)
(iii)
(iv)
(v)
(vi)
(vii)
1+ 160.0396
1+ 188.0342
1+ 201.0656
1+ 132.0434
1+ 259.0709
1+ 305.0768
1+ 160.0392
1+ 358.1029
1+ 404.1083
1+ 422.1194
1+ 609.1462
1+ 225.0657
1+ 146.0439
1+ 628.1671
1+ 159.0914
1+ 229.0606
1+ 545.1297
1+ 609.1457
1+ 807.2091
1+ 1012.2412
1+ 133.0850
1+ 229.0608
1+ 545.1299
1+ 609.1463
1+ 807.2116
1+ 1012.2421
m/z

b

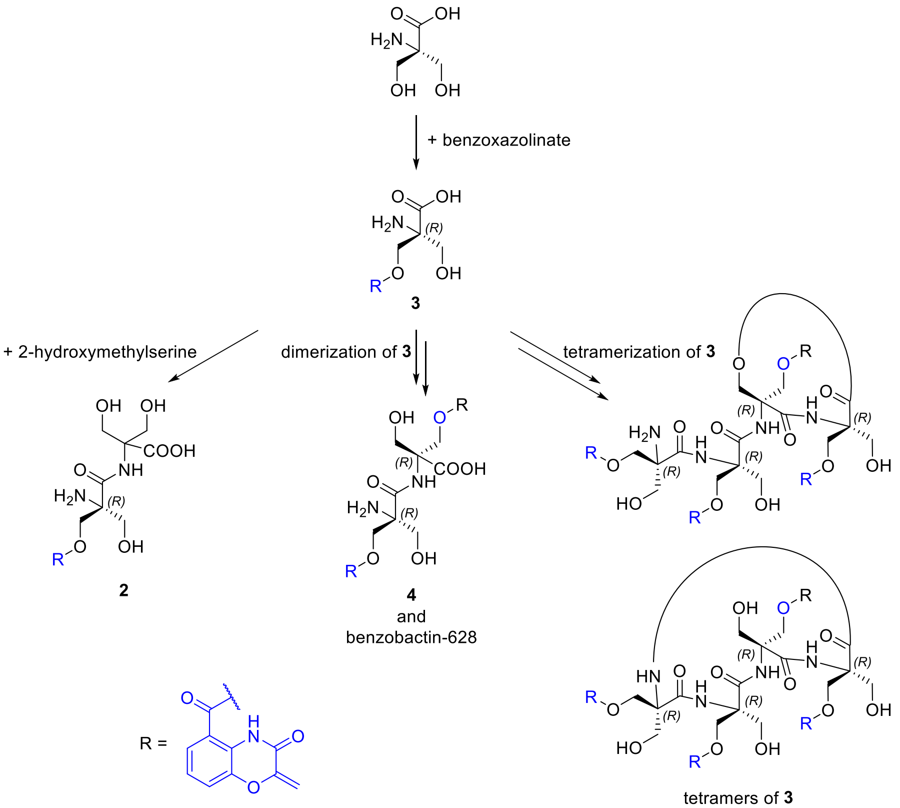

Figure S9. Tandem MS/MS analysis of benzoxazolinate and benzobactins. (a) All detected (ii-vii) benzobactins feature a diagnostic *m/z* = 188 fragment ion (grey), indicating the existence of a (i) benzoxazolinate moiety. Benzobactin C (**4**), as well as the as-yet-uncharacterized benzobactins-628 and 1021, share four diagnostic fragment ions (orange) identical to benzobactins A (**2**) and B (**3**), indicating that benzobactins C (**4**), 628, and 1021 are made up of **2** and/or **3** as structural units. As determined by HRMS, benzobactin-628 has a H_3O unit more than **4**. However, the exact structures of benzobactins-628 and 1021 could not be formulated. The blue diamond indicates the parent ions (M – H_2O + H^+). Representative data from three independent experiments are shown. (b) Proposed formation of benzobactins-628 and 1021. 2-Hydroxymethylserine is symmetrical due to two identical hydroxymethyl groups, and therefore integrations of the building block, as well as dimerization and tetramerization, would not bring diastereoisomers to benzobactin compounds, exemplified by *R*-**3**. This is consistent with the observation of no (diastereo)isomers for compounds **2**, **3**, **4**, and benzobactins-628. Therefore, benzobactins-1012 a and b are highly unlikely to be a pair of stereoisomers. Instead, they are assumed to be structural isomers with a difference in forming linkages between 2-hydroxymethylserines via ester bond(s) or amide bond(s).

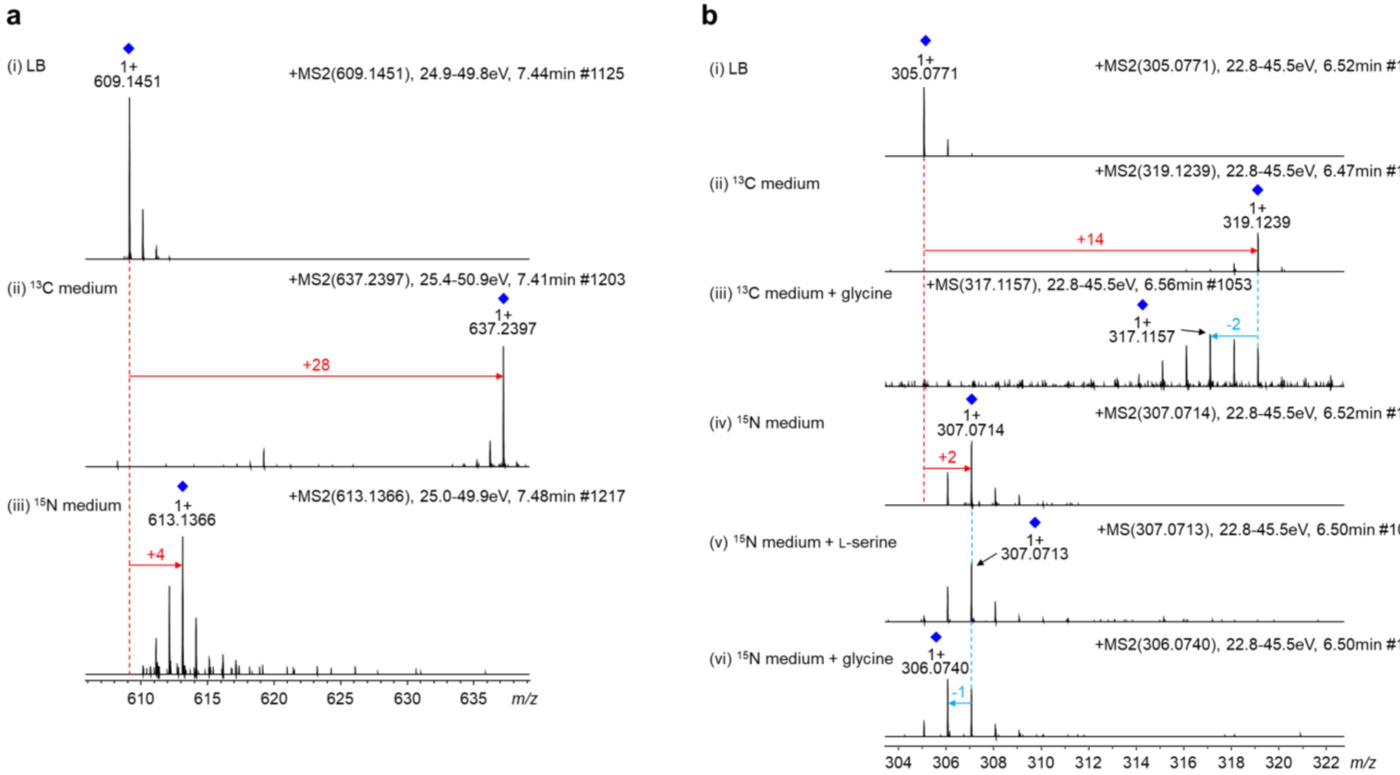

Figure S10. MS identification of benzobactin C (**4**) by isotope labeling experiments and MS verification of 2-hydroxymethylserine derived from glycine as exemplified by benzobactin B (**3**). (a) Structural elucidation of benzobactin C (**4**) by MS analyses of the induced P_{BAD} *pbzA* mutant in (i) LB, (ii) ^{13}C, and (iii) ^{15}N media. A mass shift of 28 Da in (ii) ^{13}C medium and that of 4 Da in (ii) ^{15}N medium indicated that **4** has 28 carbons and four nitrogens. Together with **4** having twice the mass of **3** – H_2O, **4** as a dimer of **3** via an amide bond linkage can be envisaged, which is also supported by the diagnostic MS fragment of 305.0768 $[M + H]^+$ in **4** (Figure S9). The number of carbon and nitrogen atoms was confirmed by (ii) ^{13}C and (iii) ^{15}N labeling media. (b) MS verification of 2-hydroxymethylserine derived from glycine in the induced P_{BAD} *pbzA* mutant by isotope labeling and inverse feeding experiments. Compared to the parent ion in (ii) the ^{13}C medium, a mass shift of (iii) -2 Da in the inverse feeding experiments with glycine in the ^{13}C medium background indicated the incorporation of a glycine residue into **3**. While (iv versus v) no mass shifts were observed in the inverse feeding experiments with L-serine in ^{15}N medium background, a mass shift of (vi) -1 Da in the inverse feeding experiments with glycine in ^{15}N medium background indicated the incorporation of a glycine residue into **3**. These data confirmed glycine being the original building block of 2-hydroxymethylserine. Arrows, dash lines, and numbers indicate positive mass shifts, while those in light blue indicate negative. The blue diamond indicates the parent ions ($M – H_2O + H^+$). Representative data from three independent experiments are shown.

a

	PbzB	AsmD	FmoH
PbzB		72%	74%
AsmD	72%		73%
FmoH	74%	73%	

b

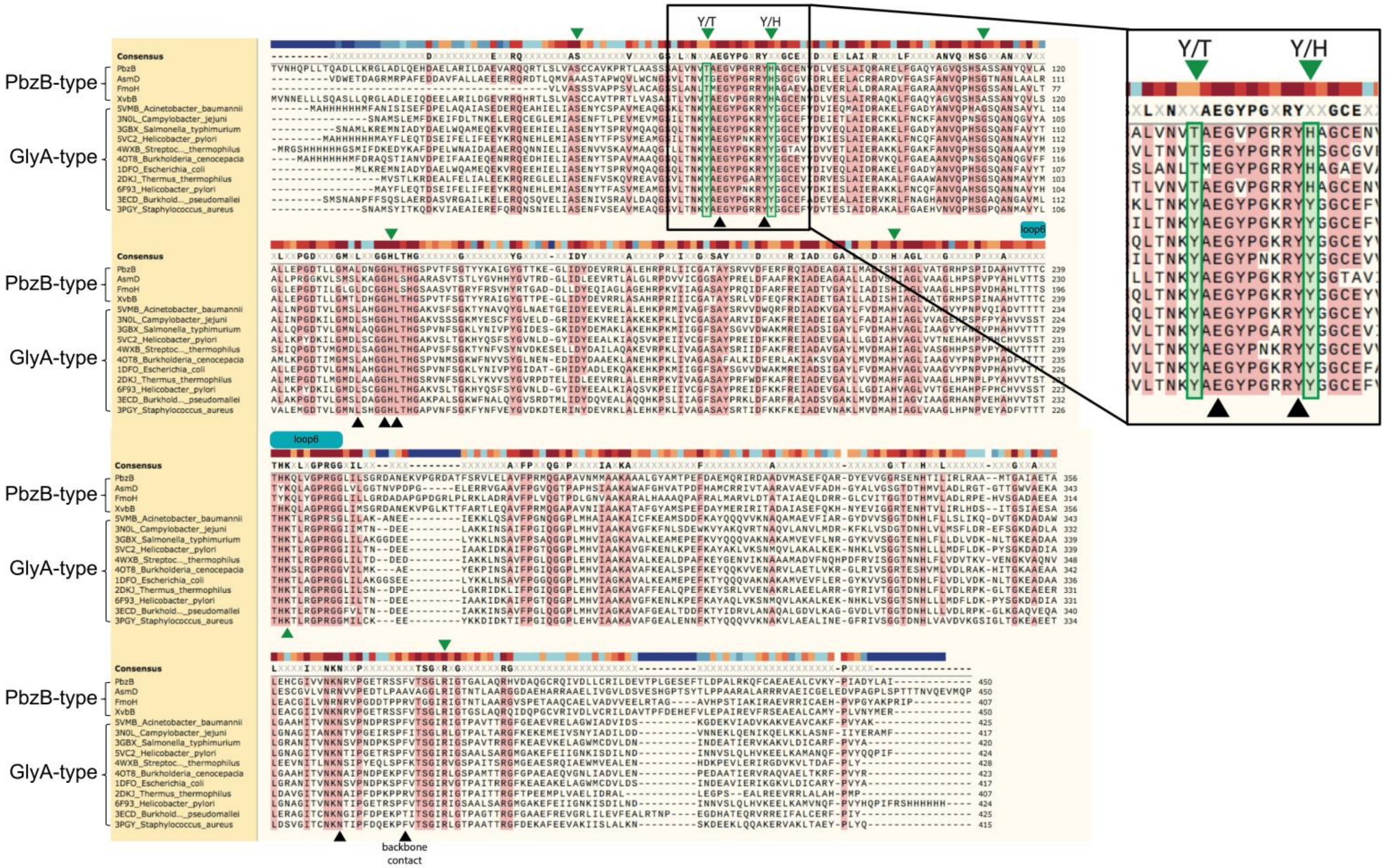

Figure S11. Multiple-sequence alignment of PbzB with other glycine/serine hydroxymethyltransferases. (a) Protein sequence similarity analysis of PbzB, AsmD (QCE20599.1), and FmoH (BAP16692.1) using Geneious Prime Cluster Omega Alignment Blosum45 (threshold = 0) scoring matrix. (b) Multiple sequence alignment of PbzB with its homologs AsmD, FmoH, XvbB, as well as other structurally characterized glycines/serine hydroxymethyltransferases. The used serine hydroxymethyltransferases were hits retrieved from a Dali search using the PbzB apo-structure in this study as the search query. The consensus threshold was set to >85%. The green arrows mark the residues involved in the coordination of the substrate (glycine or serine) bound to the co-factor PLP thereby either forming the external aldimine PLG or PLS. The lysine residue within conserved loop 6 (green arrow) forms a Schiff base with PLP. The black arrows highlight the residues involved in the binding of mTHF. The right panel shows a zoom into the alignment pointing to the difference between the specialized PbzB-type serine hydroxymethyltransferases and other GlyA-type glycine/serine hydroxymethyltransferases involved in the central metabolism of amino acid biosynthesis.

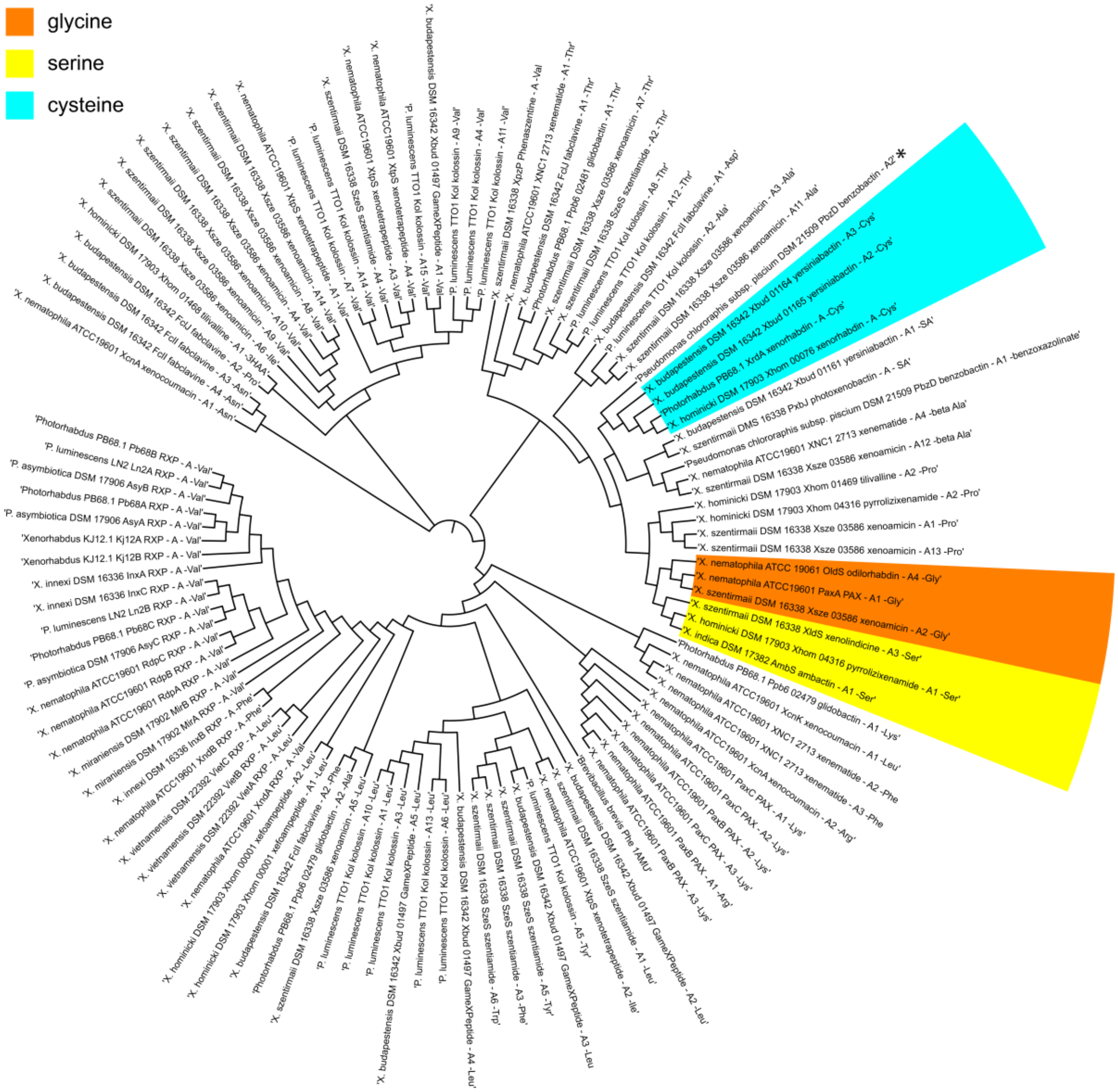

Figure S12. Phylogenetic analysis of PbzD-A_2 with other biochemically characterized A domains from *Xenorhabdus* and *Photorhabdus* strains by FastTree2.1.11 (ref[27, 28]). PbzD-A_2 (asterisk) falls into the clade of A domain with cysteine specificity (light blue) and is separate from those with glycine (orange) or serine (yellow) specificity. The tree is based on protein sequences from core motifs A4 (234) to A5 (331) which are used to determine substrate specificity.[31]

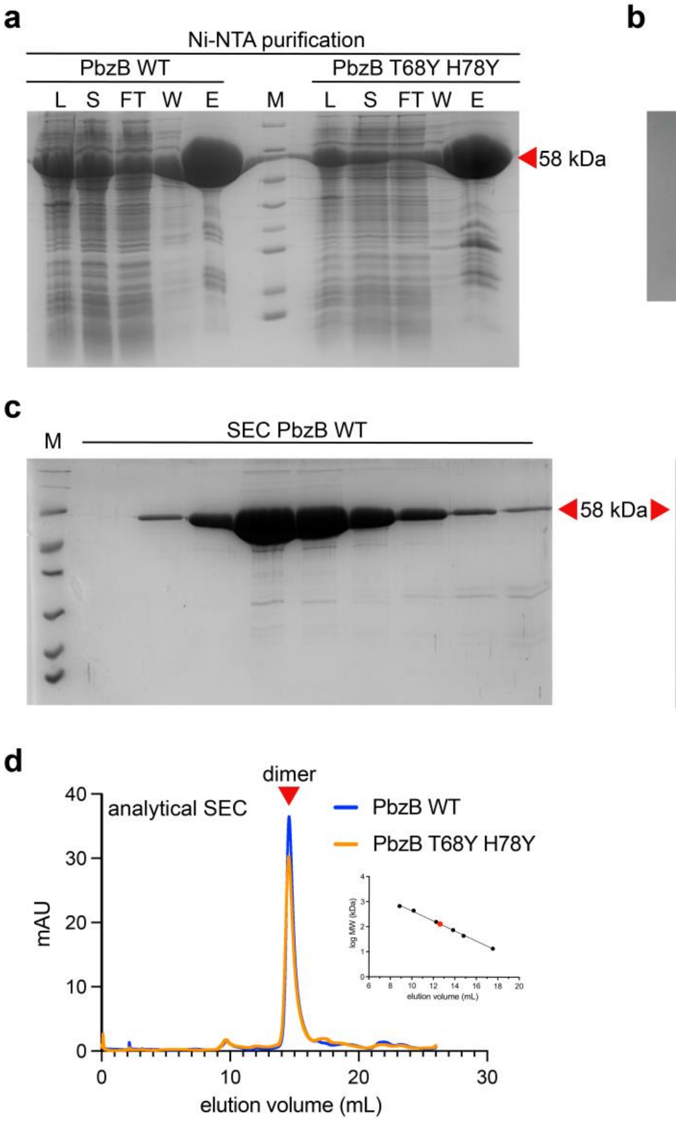

Figure S13. Purification of PbzB wild-type (WT) and PbzB T68Y H79Y mutant. (a) SDS-PAGE analysis of PbzB WT and PbzB T68Y H79Y mutant after purification by Ni-NTA. L, cell lysate; S, supernatant; FT, flow-through; W, wash; E, elution; M, marker. (b) Phenotypes of WT and mutant. The WT shows a yellowish phenotype that was lost in the mutant. Presumably, correct binding/positioning of mTHF was hindered in the mutant. (c) SDS-PAGE analysis of the WT and mutant after purification by size exclusion chromatography. (d) Chromatograms of size exclusion chromatography of the WT and mutant. The expected size of N-terminal His_6-SUMO-tag PbzB (58 kDa) was indicated by a red triangle. Representative data from three independent experiments are shown.

a

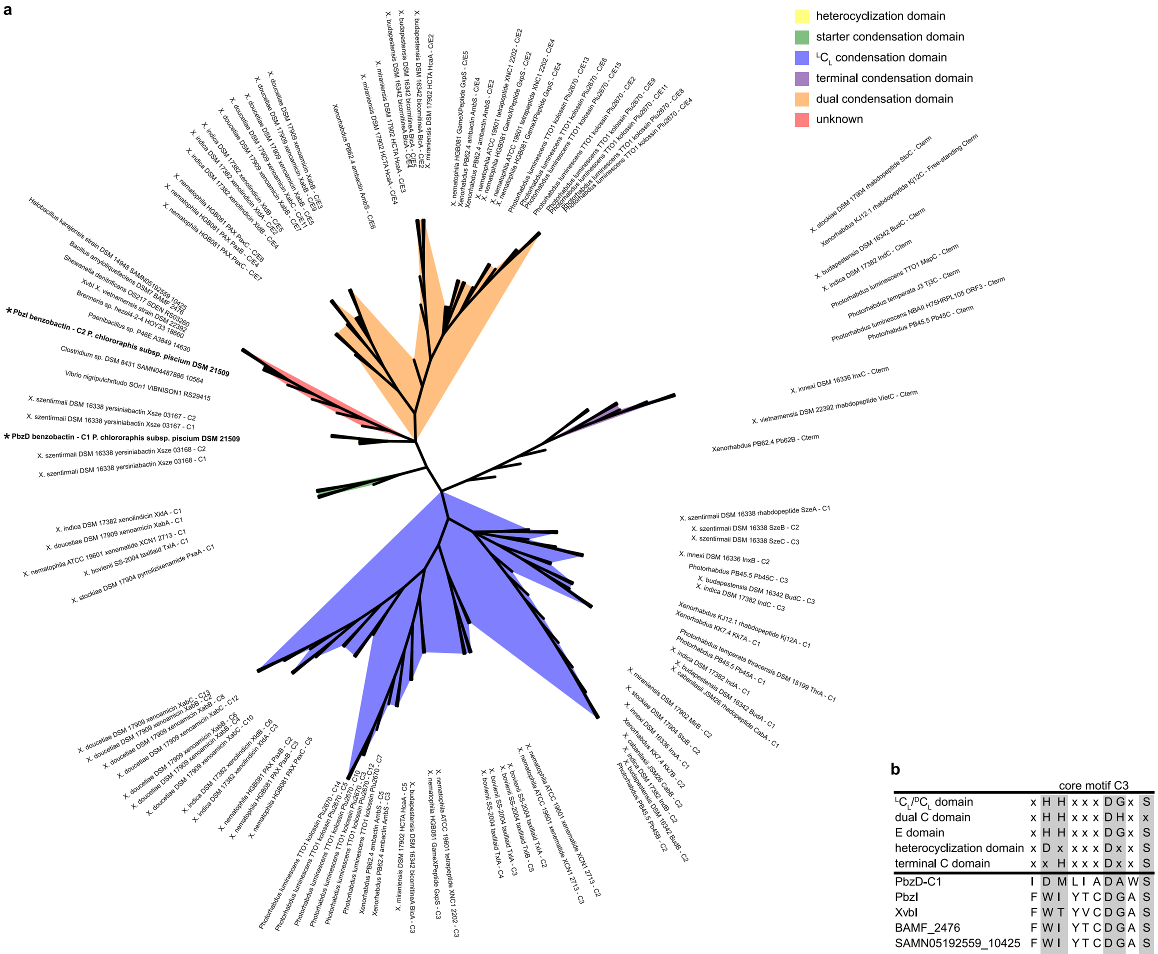

b

	core motif C3									
$^{L}C_{L}/^{D}C_{L}$ domain	x	H	H	x	x	x	D	G	x	S
dual C domain	x	H	H	x	x	x	D	H	x	x
E domain	x	H	H	x	x	x	D	G	x	S
heterocyclization domain	x	D	x	x	x	x	D	x	x	S
terminal C domain	x	x	H	x	x	x	D	x	x	S
PbzD-C1	I	D	M	L	I	A	D	A	W	S
PbzI	F	W	I	Y	T	C	D	G	A	S
XvbI	F	W	T	Y	V	C	D	G	A	S
BAMF_2476	F	W	I	Y	T	C	D	G	A	S
SAMN05192559_10425	F	W	I	Y	T	C	D	G	A	S
HOY33_18660	F	W	V	F	T	C	D	G	A	S
SDEN_RS03260	F	W	I	F	T	C	D	G	A	S
A3849_14630	F	W	I	F	T	C	D	G	A	S
SAMN04487886_10564	F	W	V	F	T	C	D	G	I	S
VIBNISON_ RS29415	F	W	T	F	T	C	D	G	L	S

Figure S14. Phylogenetic analysis and multiple-sequence alignment of PbzD-C_1 and PbzI with other condensation domains/enzymes from *Xenorhabdus* and *Photorhabdus* strains by FastTree2.1.11 (ref[27, 28]). (a) PbzD-C_1 (asterisk) falls into the clade of heterocyclization domains (yellow), while PbzI (asterisk) and its homologs from benzobactin-related BGCs are separate from all other condensation domains/enzymes (red). Heterocyclization domains (yellow) catalyze both peptide bond formation of two amino acids and subsequent intramolecular heterocyclization of cysteine, serine, or threonine. Starter condensation domains (green) acylate the first amino acid with a fatty acid or polyketide moiety. $^{L}C_{L}$ condensation domains (dark blue) catalyze a peptide bond formation between two L-amino acids. Terminal condensation domains (purple) catalyze the release of the T-domains tethered peptidyl chain. Dual condensation domains (orange) catalyze both epimerization and condensation. The tree is based on protein sequences of full-length condensation domains/enzymes. (b) A multiple-sequence alignment shows that PbzI and its homologs lack the conserved histidine or aspartic acid in the first and second positions of core motif C3. Conserved amino acids in the core motif C3 are indicated with shapes of gray.

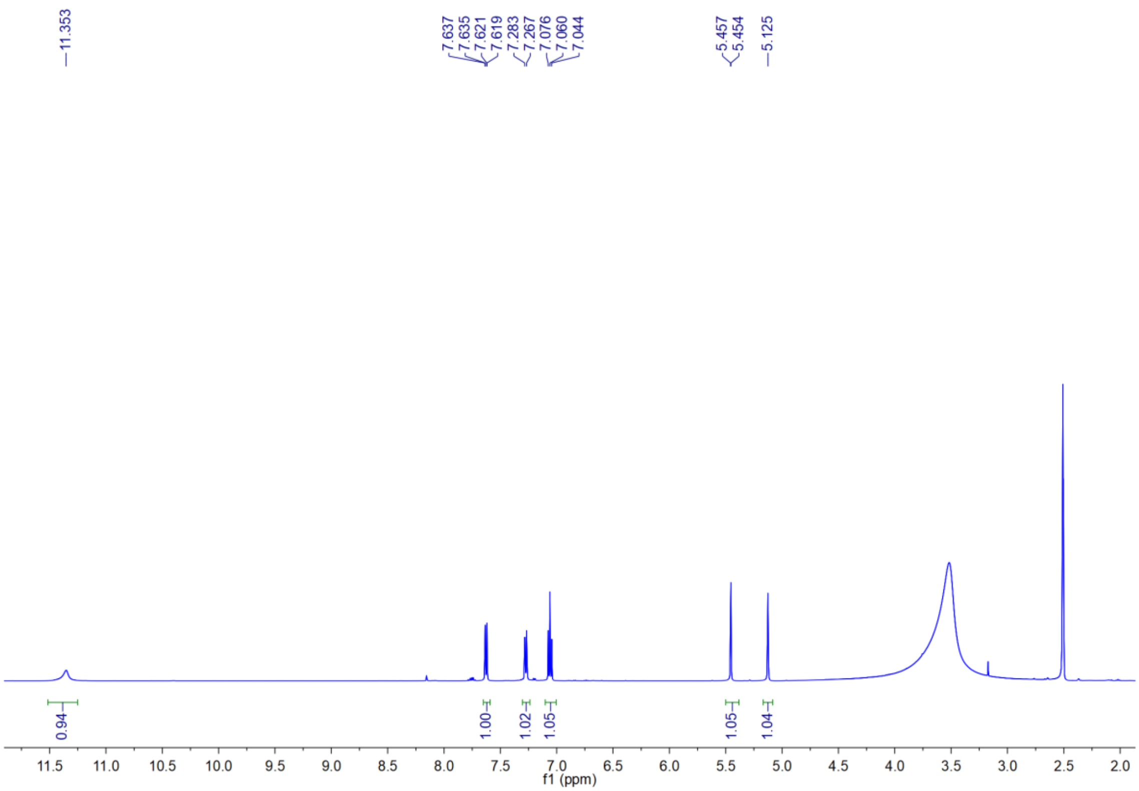

Figure S15. ^{1}H NMR spectrum of benzoxazolinate (**1**) in DMSO-d_6.

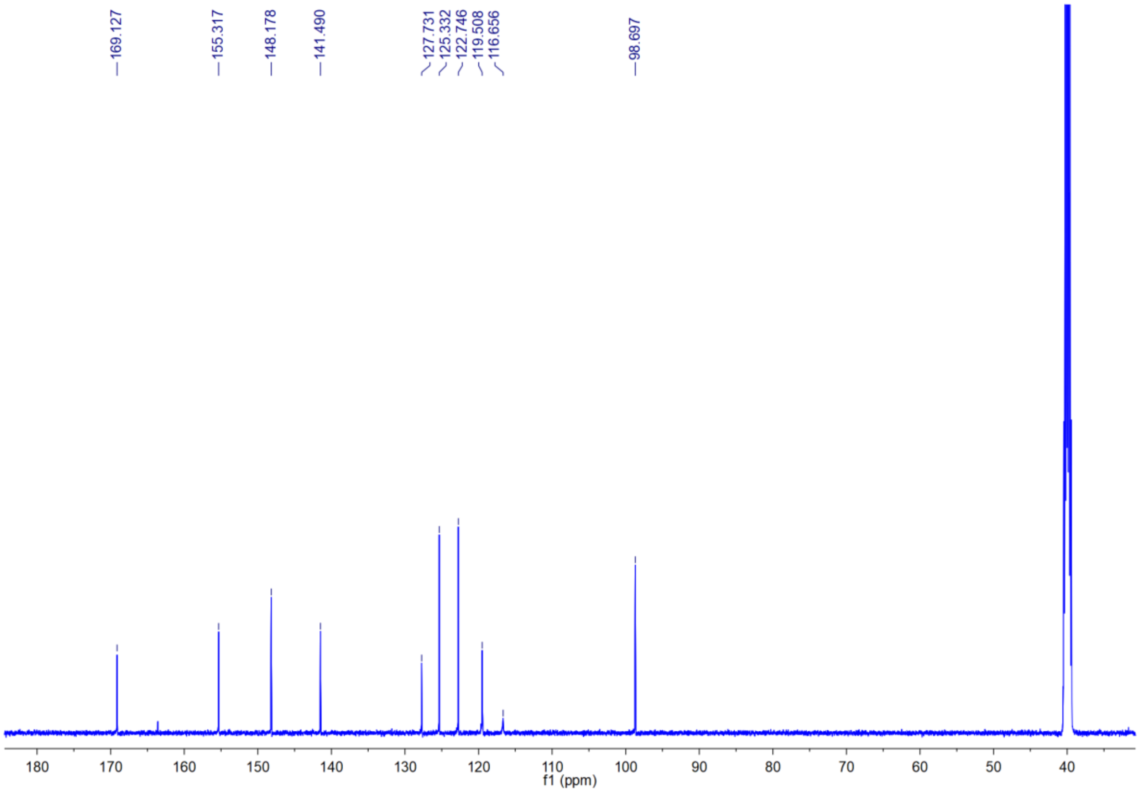

Figure S16. ^{13}C NMR spectrum of benzoxazolinate (**1**) in DMSO-d_6.

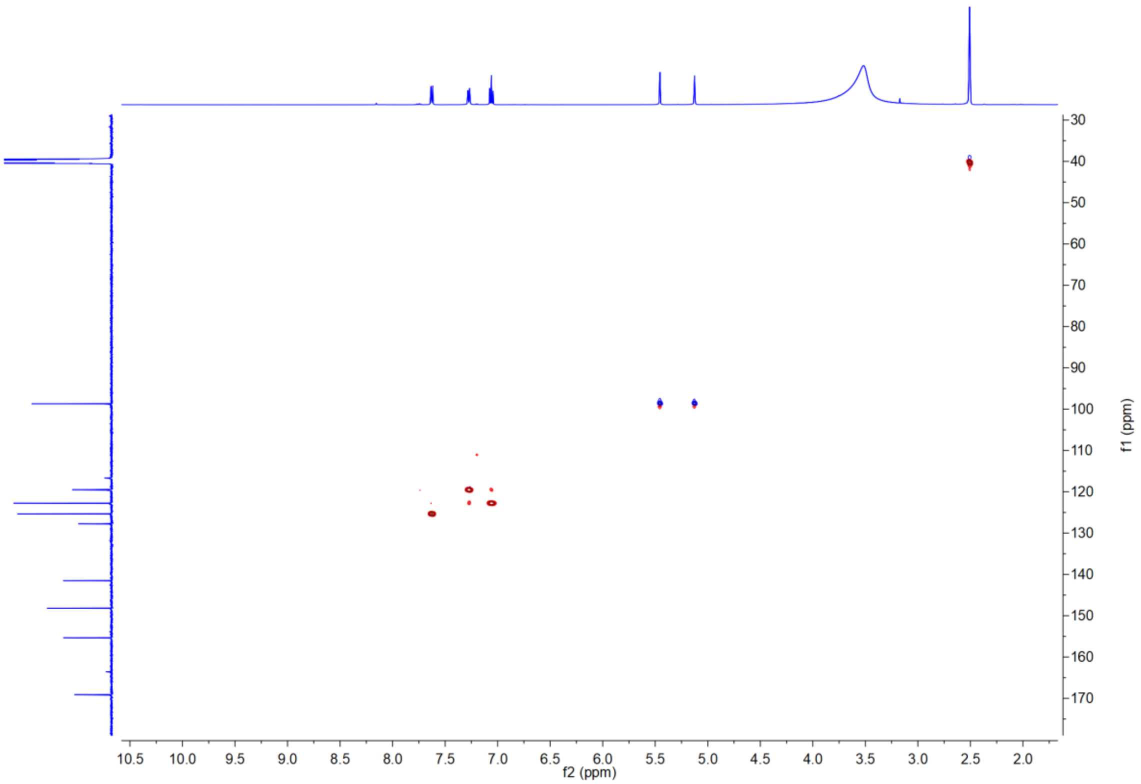

Figure S17. HSQC spectrum of benzoxazolinate (**1**) in DMSO-d_6.

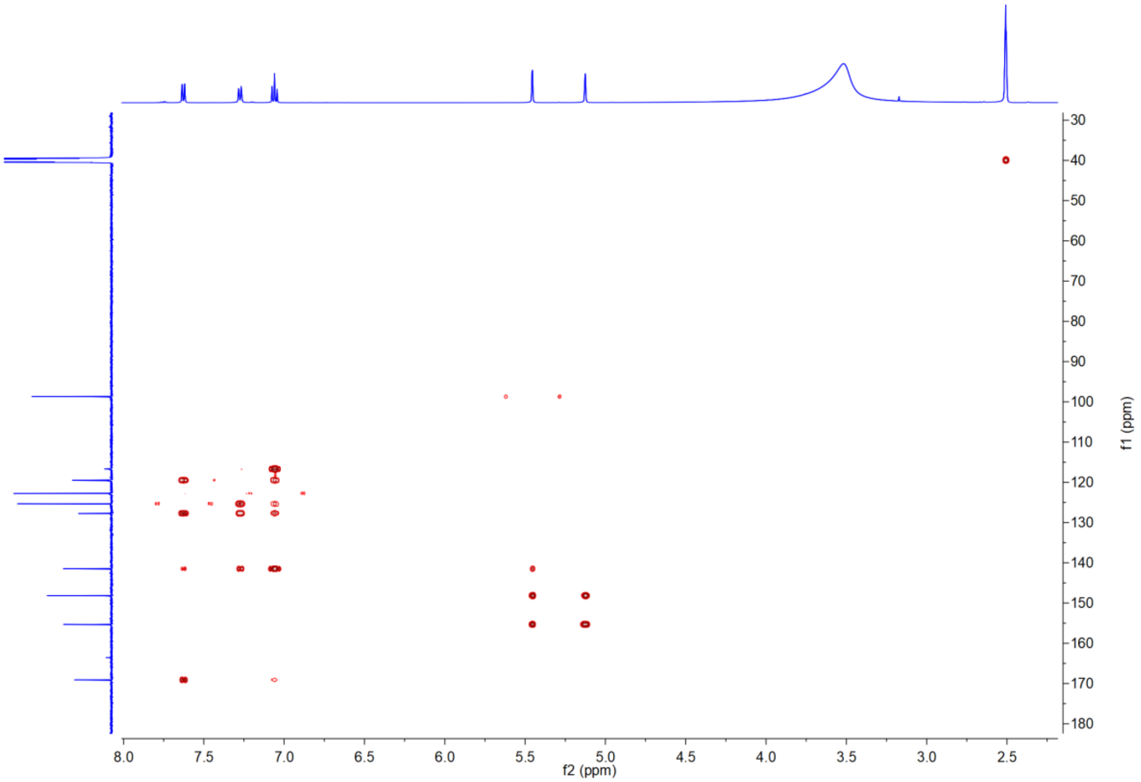

Figure S18. HMBC spectrum of benzoxazolinate (**1**) in DMSO-d_6.

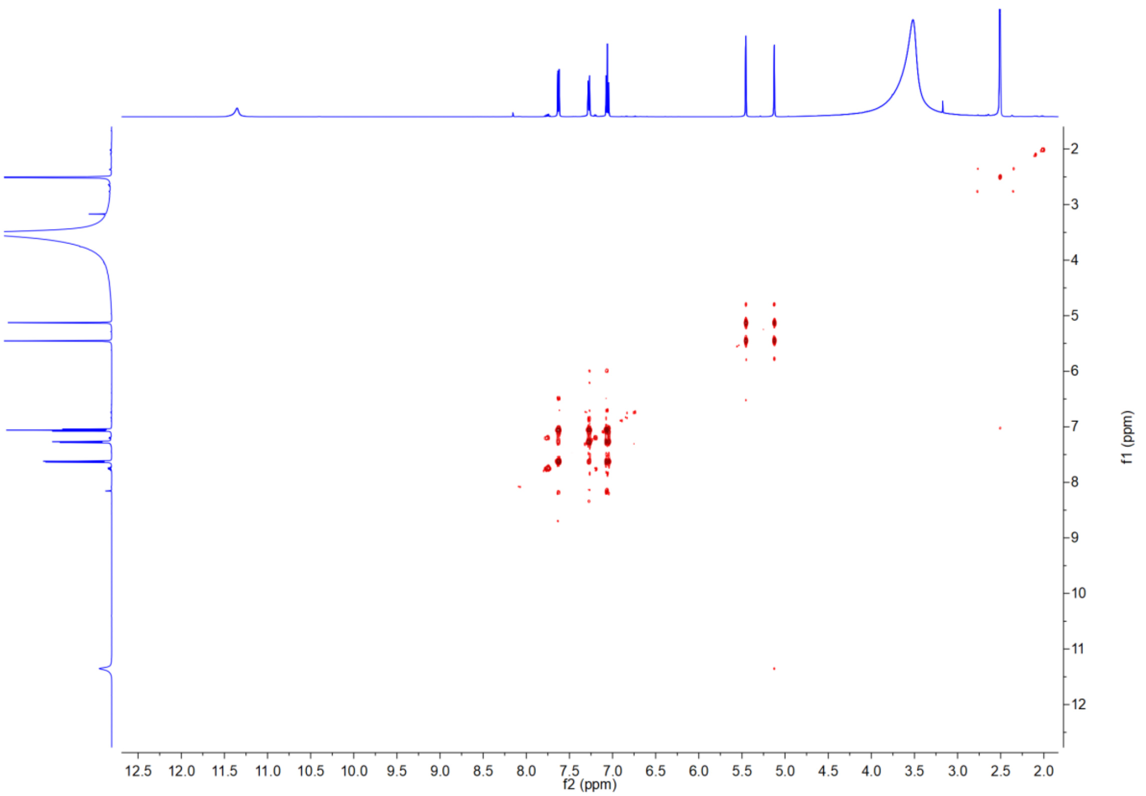

Figure S19. ^1H-^1H COSY spectrum of benzoxazolinate (**1**) in DMSO-d_6.

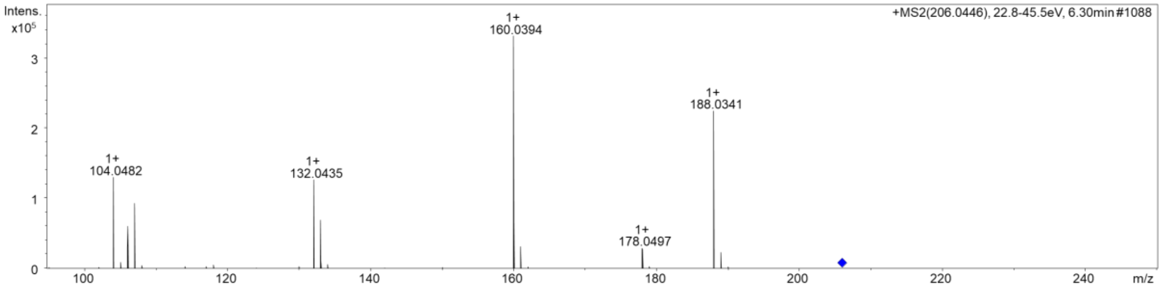

Figure S20. HR-ESI-MS of benzoxazolinate (**1**).

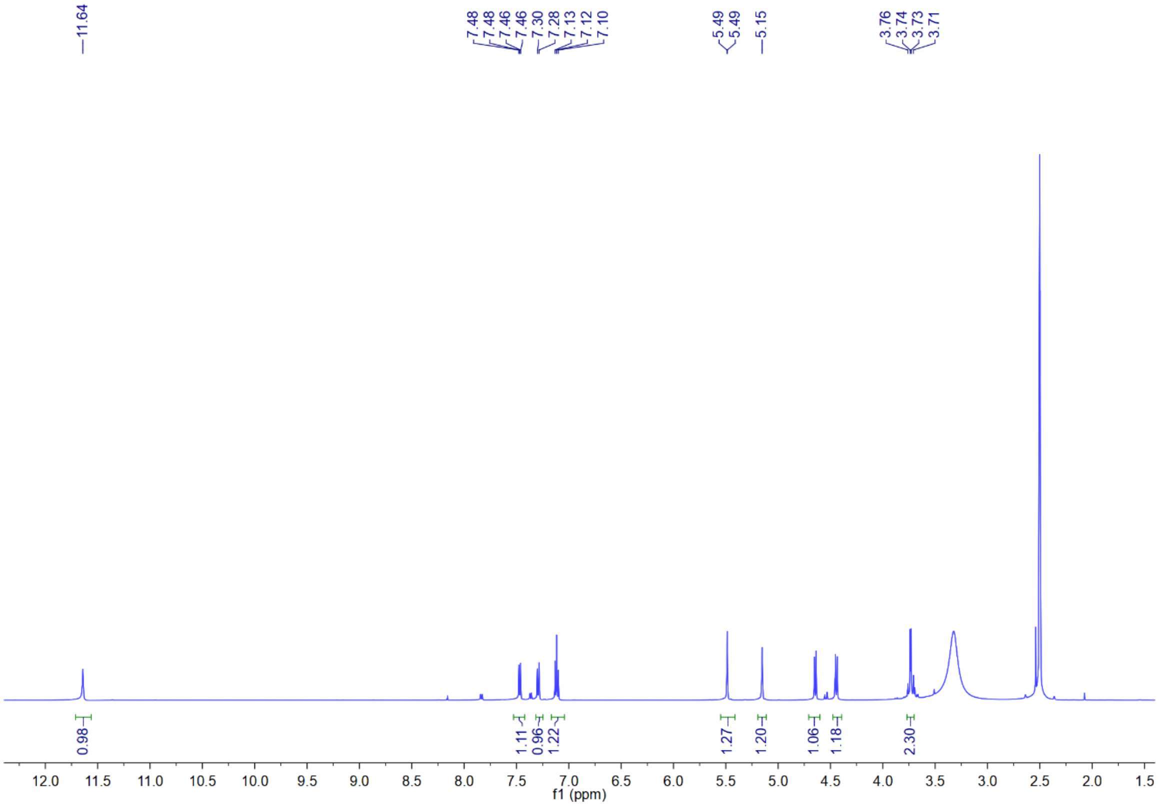

Figure S21. ^{1}H NMR spectrum of benzobactin B (**3**) in DMSO-d_6.

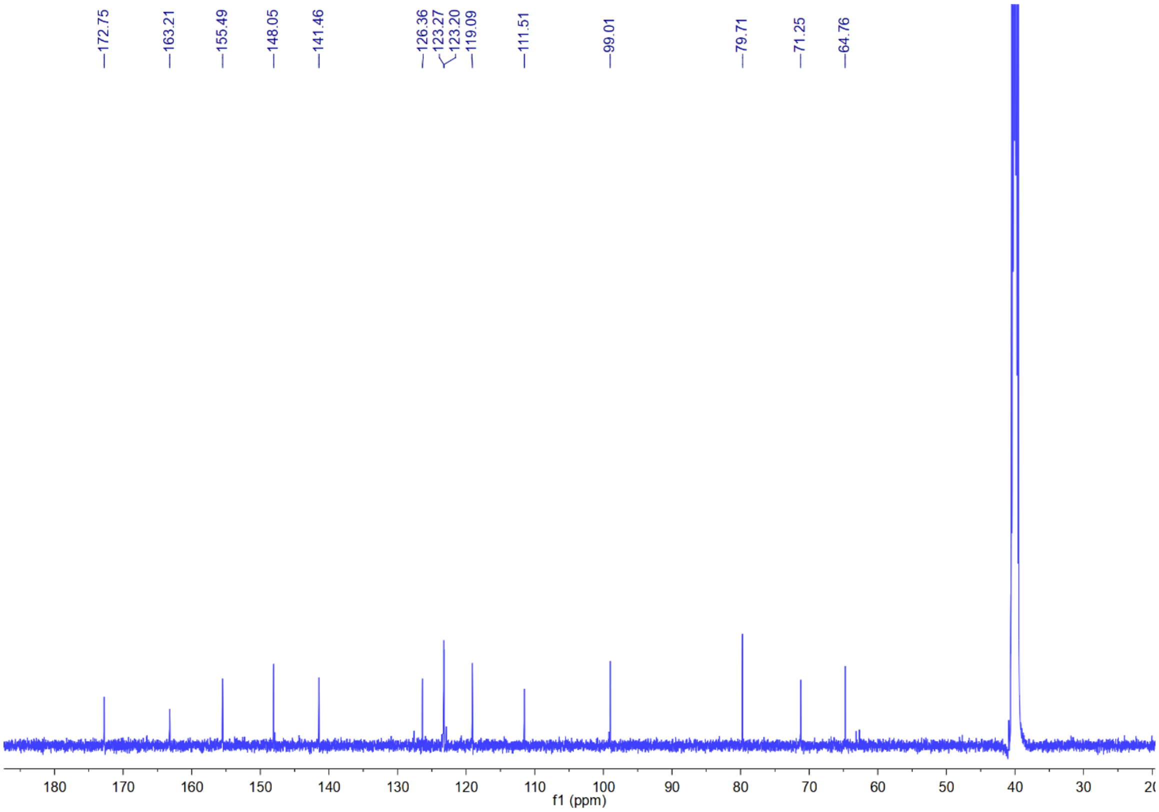

Figure S22. ^{13}C NMR spectrum of benzobactin B (**3**) in DMSO-d_6**.**

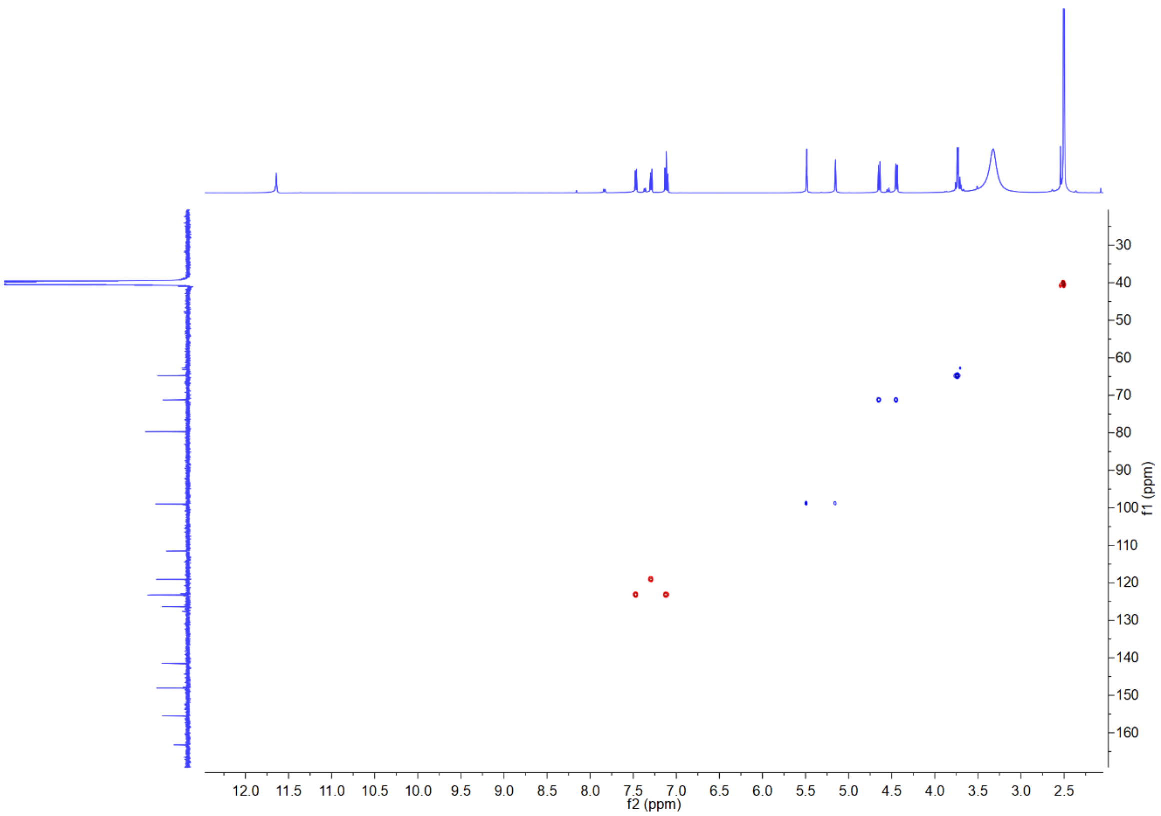

Figure S23. HSQC spectrum of benzobactin B (**3**) in DMSO-d_6.

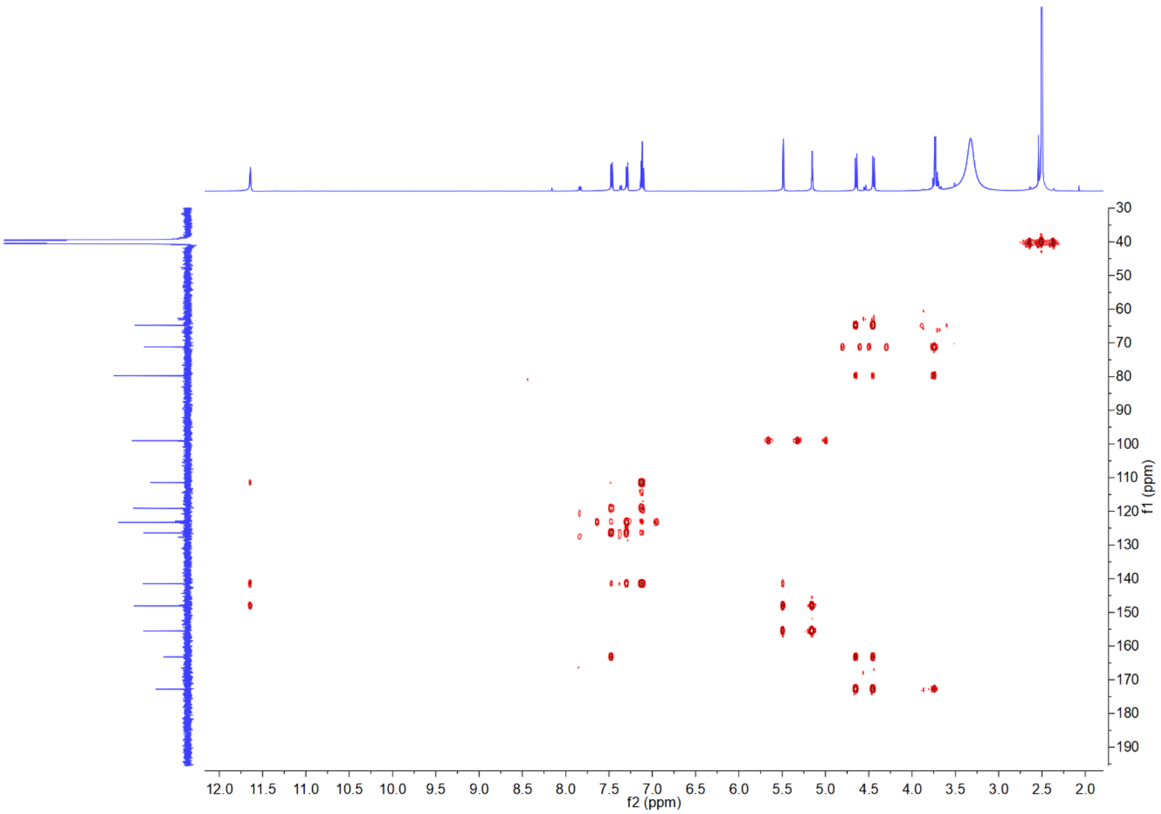

Figure S24. HMBC spectrum of benzobactin B (**3**) in DMSO-d_6.

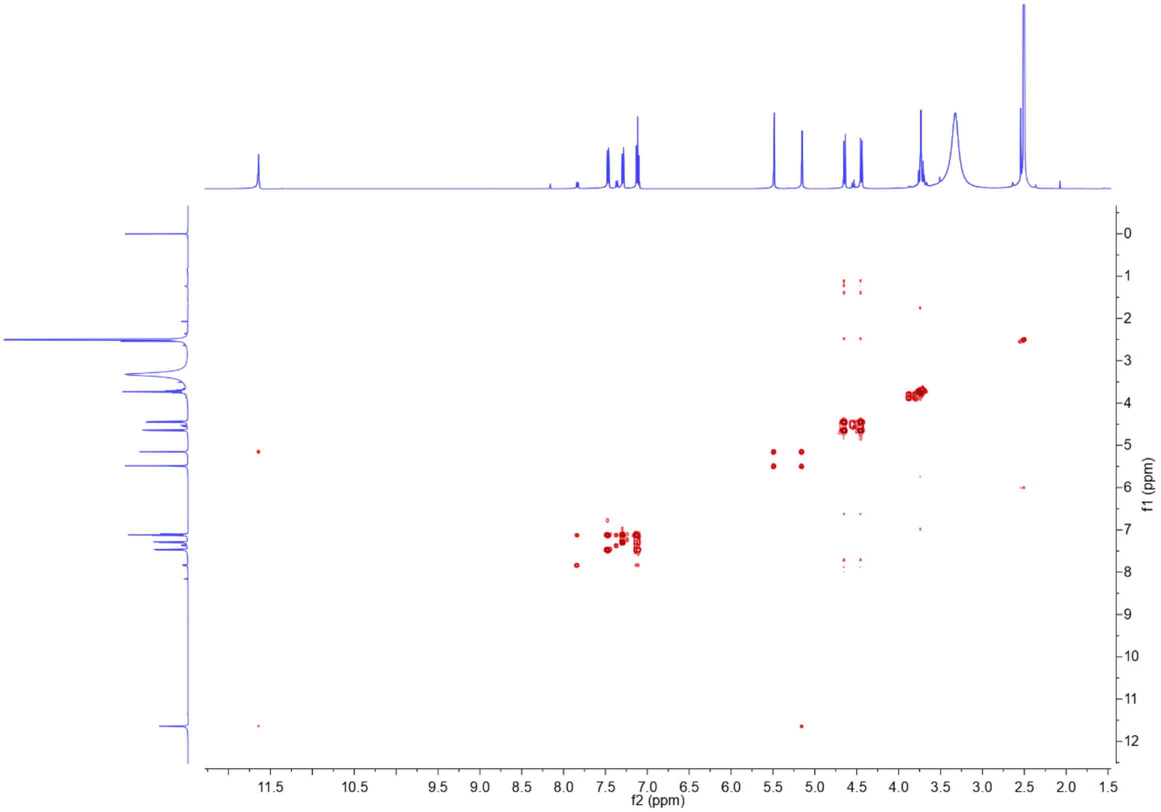

Figure S25. ^{1}H-^{1}H COSY spectrum of benzobactin B (**3**) in DMSO-d_6.

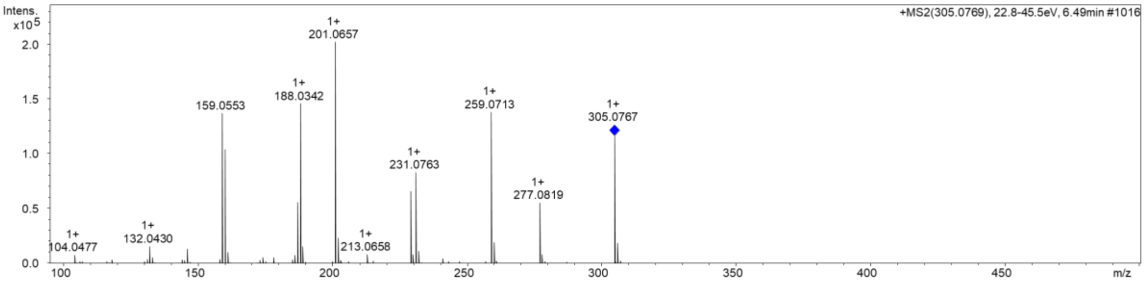

Figure S26. HR-ESI-MS of benzobactin B (**3**).

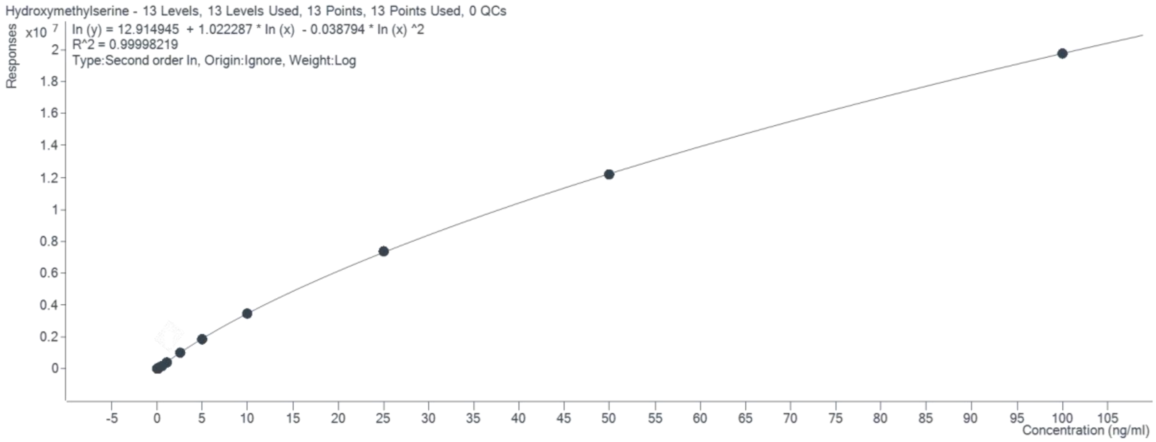

Figure S27. Standard curve of 2-hydroxymethylserine for the determination of PbzB kinetics for D-/L-serine, as well as in vitro assys of PbzB with D-serine and glycine as substrates.

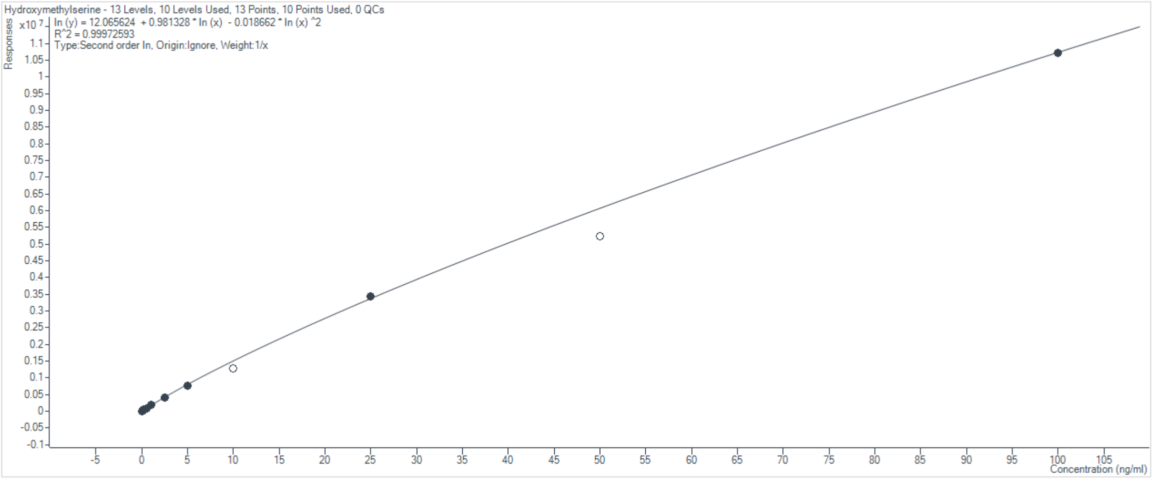

Figure S28. Standard curve of 2-hydroxymethylserine for in vitro assys of PbzB with L-serine as a substrate.

References

[1] R. R. Wick, L. M. Judd, C. L. Gorrie, K. E. Holt, *PLoS Comput. Biol.* **2017**, *13*, e1005595.

[2] K. Blin, S. Shaw, K. Steinke, R. Villebro, N. Ziemert, S. Y. Lee, M. H. Medema, T. Weber, *Nucleic Acids Res.* **2019**, *47*, W81-W87.

[3] E. Bode, A. K. Heinrich, M. Hirschmann, D. Abebew, Y.-N. Shi, T. D. Vo, F. Wesche, Y.-M. Shi, P. Grün, S. Simonyi, N. Keller, Y. Engel, S. Wenski, R. Bennet, S. Beyer, I. Bischoff, A. Buaya, S. Brandt, I. Cakmak, H. Çimen, S. Eckstein, D. Frank, R. Fürst, M. Gand, G. Geisslinger, S. Hazir, M. Henke, R. Heermann, V. Lecaudey, W. Schäfer, S. Schiffmann, A. Schüffler, R. Schwenk, M. Skaljac, E. Thines, M. Thines, T. Ulshöfer, A. Vilcinskas, T. A. Wichelhaus, H. B. Bode, *Angew. Chem. Int. Ed.* **2019**, *58*, 18957-18963.

[4] C. Fu, W. P. Donovan, O. Shikapwashya-Hasser, X. Ye, R. H. Cole, *PLoS One* **2015**, *9*, e115318.

[5] H. Song, C. Rao, Z. Deng, Y. Yu, J. H. Naismith, *Angew. Chem. Int. Ed.* **2020**, *59*, 6054-6061.

[6] N. Wang, J. D. Rudolf, L.-B. Dong, J. Osipiuk, C. Hatzos-Skintges, M. Endres, C.-Y. Chang, G. Babnigg, A. Joachimiak, G. N. Phillips, B. Shen, *Nat. Chem. Biol.* **2018**, *14*, 730-737.

[7] Molecular operating environment (MOE), *2019.01; Chemical Computing Group ULC, 1010 Sherbooke St. West, Suite# 910, Montreal, QC, Canada, H3A 2R7, 2020*.

[8] M. Lv, J. Zhao, Z. Deng, Y. Yu, *Chem. Biol.* **2015**, *22*, 1313-1324.

[9] A. K. Bera, V. Atanasova, S. Gamage, H. Robinson, J. F. Parsons, *Acta Crystallogr. D* **2010**, *66*, 664-672.

[10] E. Conti, T. Stachelhaus, M. A. Marahiel, P. Brick, *EMBO J.* **1997**, *16*, 4174-4183.

[11] M. Levitt, R. Sharon, *Proc. Natl. Acad. Sci. USA* **1988**, *85*, 7557-7561.

[12] T. Fechteler, U. Dengler, D. Schomburg, *J. Mol. Biol.* **1995**, *253*, 114-131.

[13] Y.-M. Shi, M. Hirschmann, Y.-N. Shi, S. Ahmed, D. Abebew, N. J. Tobias, P. Grün, J. J. Crames, L. Pöschel, W. Kuttenlochner, C. Richter, J. Herrmann, R. Müller, A. Thanwisai, S. J. Pidot, T. P. Stinear, M. Groll, Y. Kim, H. B. Bode, *Nat. Chem.* **2022**, *14*, 701-712.

[14] P. Theveneau, R. Baker, R. Barrett, A. Beteva, M. W. Bowler, P. Carpentier, H. Caserotto, D. d. Sanctis, F. Dobias, D. Flot, M. Guijarro, T. Giraud, M. Lentini, G. A. Leonard, M. Mattenet, A. A. McCarthy, S. M. McSweeney, C. Morawe, M. Nanao, D. Nurizzo, S. Ohlsson, P. Pernot, A. N. Popov, A. Round, A. Royant, W. Schmid, A. Snigirev, J. Surr, C. Mueller-Dieckmann, *J. Phys. Conf. Ser.* **2013**, *425*, 012001.

[15] W. Kabsch, *Acta Crystallogr. D* **2010**, *66*, 133-144.

[16] A. McCoy, *Acta Crystallogr. D* **2007**, *63*, 32-41.

[17] P. Emsley, K. Cowtan, *Acta Crystallogr. D* **2004**, *60*, 2126-2132.

[18] D. Liebschner, P. V. Afonine, M. L. Baker, G. Bunkoczi, V. B. Chen, T. I. Croll, B. Hintze, L.-W. Hung, S. Jain, A. J. McCoy, N. W. Moriarty, R. D. Oeffner, B. K. Poon, M. G. Prisant,

R. J. Read, J. S. Richardson, D. C. Richardson, M. D. Sammito, O. V. Sobolev, D. H. Stockwell, T. C. Terwilliger, A. G. Urzhumtsev, L. L. Videau, C. J. Williams, P. D. Adams, *Acta Crystallogr. D* **2019**, *75*, 861-877.

[19] W. L. DeLano, *Schrödinger, LLC* **2002**.

[20] N. W. Moriarty, D. Liebschner, H. E. Klei, N. Echols, P. V. Afonine, J. J. Headd, B. K. Poon, P. D. Adams, *Protein Sci.* **2018**, *27*, 182-194.

[21] M. Gualtieri, J.-C. Ogier, S. Pagès, A. Givaudan, S. Gaudriault, *Genome Announc.* **2014**, *2*, e00190-00114.

[22] S. E. Burr, S. Gobeli, P. Kuhnert, E. Goldschmidt-Clermont, J. Frey, *Int. J. Syst. Evol. Microbiol.* **2010**, *60*, 2753-2757.

[23] E. Bode, A. O. Brachmann, C. Kegler, R. Simsek, C. Dauth, Q. Zhou, M. Kaiser, P. Klemmt, H. B. Bode, *ChemBioChem* **2015**, *16*, 1115-1119.

[24] F. I. Nollmann, A. K. Heinrich, A. O. Brachmann, C. Morisseau, K. Mukherjee, A. M. Casanova-Torres, F. Strobl, D. Kleinhans, S. Kinski, K. Schultz, M. L. Beeton, M. Kaiser, Y. Y. Chu, L. Phan Ke, A. Thanwisai, K. A. Bozhüyük, N. Chantratita, F. Gotz, N. R. Waterfield, A. Vilcinskas, E. H. Stelzer, H. Goodrich-Blair, B. D. Hammock, H. B. Bode, *ChemBioChem* **2015**, *16*, 766-771.

[25] M. A. Marahiel, T. Stachelhaus, H. D. Mootz, *Chem. Rev.* **1997**, *97*, 2651-2674.

[26] E. Dornisch, J. Pletz, R. A. Glabonjat, F. Martin, C. Lembacher-Fadum, M. Neger, C. Högenauer, K. Francesconi, W. Kroutil, K. Zangger, R. Breinbauer, E. L. Zechner, *Angew. Chem. Int. Ed.* **2017**, *56*, 14753-14757.

[27] M. N. Price, P. S. Dehal, A. P. Arkin, *Mol. Biol. Evol.* **2009**, *26*, 1641-1650.

[28] M. N. Price, P. S. Dehal, A. P. Arkin, *PLoS One* **2010**, *5*, e9490.

[29] A. M. Gulick, V. J. Starai, A. R. Horswill, K. M. Homick, J. C. Escalante-Semerena, *Biochemistry* **2003**, *42*, 2866-2873.

[30] A. S. Reger, R. Wu, D. Dunaway-Mariano, A. M. Gulick, *Biochemistry* **2008**, *47*, 8016-8025.

[31] T. Stachelhaus, H. D. Mootz, M. A. Marahiel, *Chem. Biol.* **1999**, *6*, 493-505.
