# Supplementary material for: Genome Mining Enabled by Biosynthetic Characterization Uncovers a Class of Benzoxazolinate‐Containing Natural Products in Diverse Bacteria
Source: Angew Chem Int Ed Engl. 2022 Nov 17;61(51):e202206106. doi: 10.1002/anie.202206106 (PMC10098953; doi:10.1002/anie.202206106)
Supplement: Supplementary file 2 — Supporting Information [file ANIE-61-0-s001.pdf]

Full wwPDB X-ray Structure Validation Report (i)

Nov 30, 2021 – 12:24 pm GMT

PDB ID	:	7QCW
Title	:	Apo-structure of serine hydroxymethyltransferase (PbzB) involved in benzobactin biosynthesis in P. chlororaphis subsp. piscium DSM 21509
Deposited on	:	2021-11-25
Resolution	:	2.81 Å(reported)

This is a Full wwPDB X-ray Structure Validation Report.

This report is produced by the wwPDB biocuration pipeline after annotation of the structure.

We welcome your comments at *validation@mail.wwpdb.org*
A user guide is available at
https://www.wwpdb.org/validation/2017/XrayValidationReportHelp
with specific help available everywhere you see the (i) symbol.

CONFIDENTIAL VALIDATION REPORT

The following versions of software and data (see references (i)) were used in the production of this report:

MolProbity	:	4.02b-467
Xtriage (Phenix)	:	1.13
EDS	:	2.23.2
Percentile statistics	:	20191225.v01 (using entries in the PDB archive December 25th 2019)
Refmac	:	5.8.0267
CCP4	:	7.1.010 (Gargrove)
Ideal geometry (proteins)	:	Engh & Huber (2001)
Ideal geometry (DNA, RNA)	:	Parkinson et al. (1996)
Validation Pipeline (wwPDB-VP)	:	2.23.2

1 Overall quality at a glance (i)

The following experimental techniques were used to determine the structure:
X-RAY DIFFRACTION

The reported resolution of this entry is 2.81 Å.

Percentile scores (ranging between 0-100) for global validation metrics of the entry are shown in the following graphic. The table shows the number of entries on which the scores are based.

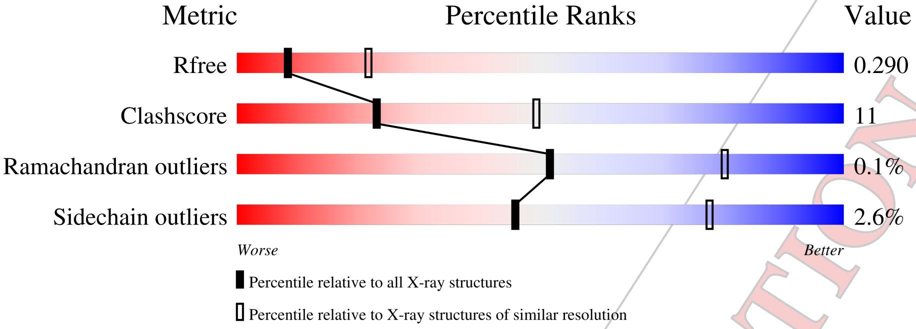

Metric	Whole archive (#Entries)	Similar resolution (#Entries, resolution range(Å))
R_{free}	130704	3617 (2.84-2.80)
Clashscore	141614	4060 (2.84-2.80)
Ramachandran outliers	138981	3978 (2.84-2.80)
Sidechain outliers	138945	3980 (2.84-2.80)

The table below summarises the geometric issues observed across the polymeric chains and their fit to the electron density. The red, orange, yellow and green segments of the lower bar indicate the fraction of residues that contain outliers for >=3, 2, 1 and 0 types of geometric quality criteria respectively. A grey segment represents the fraction of residues that are not modelled. The numeric value for each fraction is indicated below the corresponding segment, with a dot representing fractions <=5%

Mol	Chain	Length	Quality of chain
1	A	460	68% 18% 14%
1	B	460	62% 25% • 11%

CONFIDENTIAL VALIDATION REPORT

2 Entry composition (i)

There is only 1 type of molecule in this entry. The entry contains 6027 atoms, of which 0 are hydrogens and 0 are deuteriums.

In the tables below, the ZeroOcc column contains the number of atoms modelled with zero occupancy, the AltConf column contains the number of residues with at least one atom in alternate conformation and the Trace column contains the number of residues modelled with at most 2 atoms.

- Molecule 1 is a protein called Serine hydroxymethyltransferase.

Mol	Chain	Residues	Atoms					ZeroOcc	AltConf	Trace
			Total	C	N	O	S			
1	A	396	2983	1852	542	572	17	0	0	0
1	B	408	3044	1886	555	585	18	0	0	0

There are 42 discrepancies between the modelled and reference sequences:

Chain	Residue	Modelled	Actual	Comment	Reference
A	-7	SER	-	expression tag	UNP A0A3G7DQ80
A	-6	GLY	-	expression tag	UNP A0A3G7DQ80
A	-5	SER	-	expression tag	UNP A0A3G7DQ80
A	-4	HIS	-	expression tag	UNP A0A3G7DQ80
A	-3	MET	-	expression tag	UNP A0A3G7DQ80
A	-2	THR	-	expression tag	UNP A0A3G7DQ80
A	-1	MET	-	expression tag	UNP A0A3G7DQ80
A	0	GLY	-	expression tag	UNP A0A3G7DQ80
A	23	GLU	GLN	conflict	UNP A0A3G7DQ80
A	27	GLU	LYS	conflict	UNP A0A3G7DQ80
A	30	ARG	GLN	conflict	UNP A0A3G7DQ80
A	55	ARG	LEU	conflict	UNP A0A3G7DQ80
A	245	VAL	ALA	conflict	UNP A0A3G7DQ80
A	270	SER	ALA	conflict	UNP A0A3G7DQ80
A	289	MET	ILE	conflict	UNP A0A3G7DQ80
A	400	ARG	GLN	conflict	UNP A0A3G7DQ80
A	416	LEU	ILE	conflict	UNP A0A3G7DQ80
A	429	LYS	GLN	conflict	UNP A0A3G7DQ80
A	450	ILE	-	expression tag	UNP A0A3G7DQ80
A	451	LEU	-	expression tag	UNP A0A3G7DQ80
A	452	GLU	-	expression tag	UNP A0A3G7DQ80
B	-7	SER	-	expression tag	UNP A0A3G7DQ80
B	-6	GLY	-	expression tag	UNP A0A3G7DQ80
B	-5	SER	-	expression tag	UNP A0A3G7DQ80
B	-4	HIS	-	expression tag	UNP A0A3G7DQ80

Continued on next page...

CONFIDENTIAL VALIDATION REPORT

Continued from previous page...

Chain	Residue	Modelled	Actual	Comment	Reference
B	-3	MET	-	expression tag	UNP A0A3G7DQ80
B	-2	THR	-	expression tag	UNP A0A3G7DQ80
B	-1	MET	-	expression tag	UNP A0A3G7DQ80
B	0	GLY	-	expression tag	UNP A0A3G7DQ80
B	23	GLU	GLN	conflict	UNP A0A3G7DQ80
B	27	GLU	LYS	conflict	UNP A0A3G7DQ80
B	30	ARG	GLN	conflict	UNP A0A3G7DQ80
B	55	ARG	LEU	conflict	UNP A0A3G7DQ80
B	245	VAL	ALA	conflict	UNP A0A3G7DQ80
B	270	SER	ALA	conflict	UNP A0A3G7DQ80
B	289	MET	ILE	conflict	UNP A0A3G7DQ80
B	400	ARG	GLN	conflict	UNP A0A3G7DQ80
B	416	LEU	ILE	conflict	UNP A0A3G7DQ80
B	429	LYS	GLN	conflict	UNP A0A3G7DQ80
B	450	ILE	-	expression tag	UNP A0A3G7DQ80
B	451	LEU	-	expression tag	UNP A0A3G7DQ80
B	452	GLU	-	expression tag	UNP A0A3G7DQ80

CONFIDENTIAL VALIDATION REPORT

3 Residue-property plots (i)

These plots are drawn for all protein, RNA, DNA and oligosaccharide chains in the entry. The first graphic for a chain summarises the proportions of the various outlier classes displayed in the second graphic. The second graphic shows the sequence view annotated by issues in geometry. Residues are color-coded according to the number of geometric quality criteria for which they contain at least one outlier: green = 0, yellow = 1, orange = 2 and red = 3 or more. Stretches of 2 or more consecutive residues without any outlier are shown as a green connector. Residues present in the sample, but not in the model, are shown in grey.

• Molecule 1: Serine hydroxymethyltransferase

Chain A:

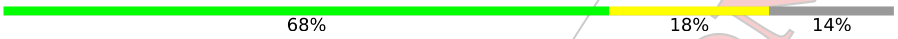

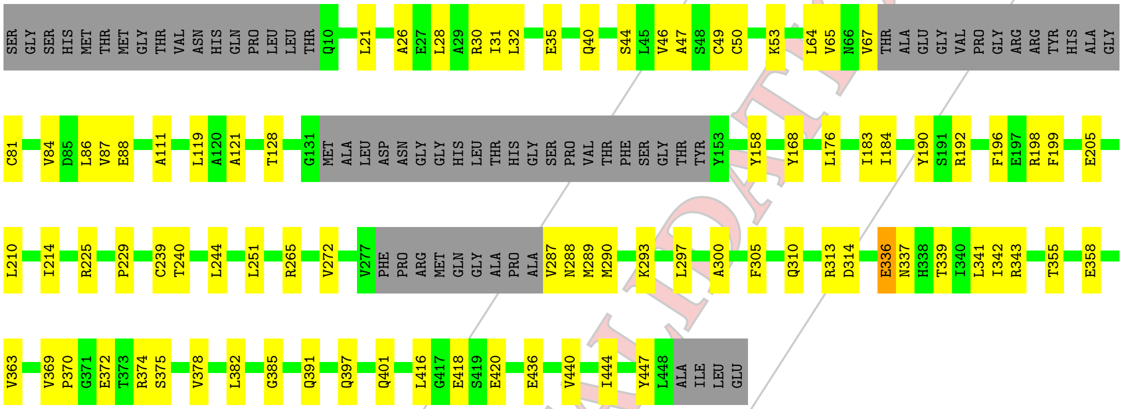

• Molecule 1: Serine hydroxymethyltransferase

Chain B:

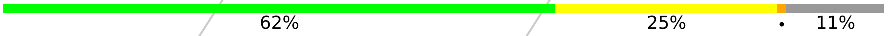

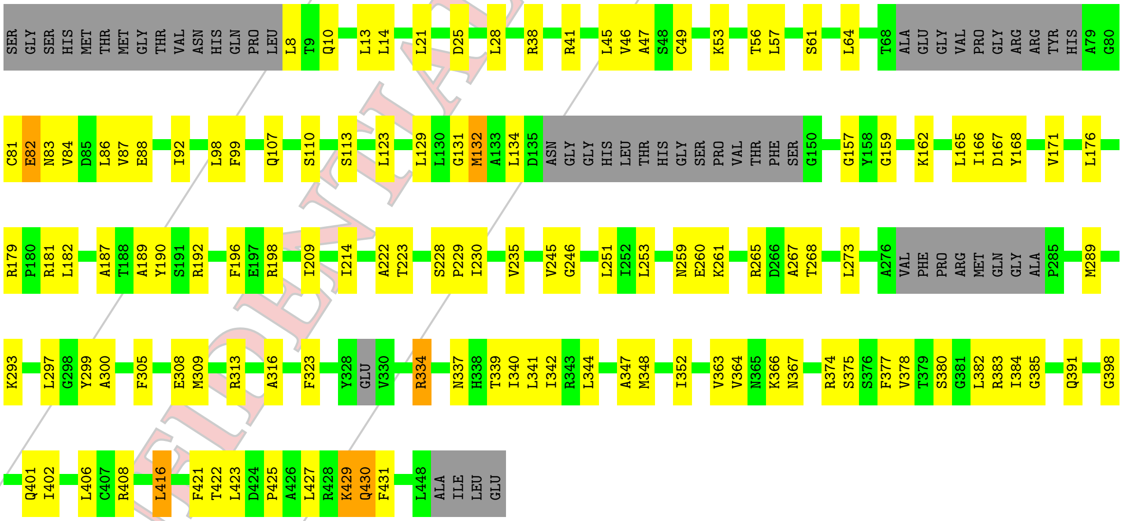

CONFIDENTIAL VALIDATION REPORT

4 Data and refinement statistics (i)

Property	Value	Source
Space group	C 1 2 1	Depositor
Cell constants<br>a, b, c, α, β, γ	132.40Å 59.64Å 112.12Å<br>90.00° 109.41° 90.00°	Depositor
Resolution (Å)	49.21 – 2.81<br>49.21 – 2.81	Depositor<br>EDS
% Data completeness<br>(in resolution range)	99.6 (49.21-2.81)<br>99.7 (49.21-2.81)	Depositor<br>EDS
R_{merge}	(Not available)	Depositor
R_{sym}	(Not available)	Depositor
$< I/\sigma(I) >$ [1]	1.68 (at 2.81Å)	Xtriage
Refinement program	PHENIX (1.18.2_3874: ???)	Depositor
R, R_{free}	0.236 , 0.295<br>0.238 , 0.290	Depositor<br>DCC
R_{free} test set	1016 reflections (5.00%)	wwPDB-VP
Wilson B-factor (Å^2)	68.5	Xtriage
Anisotropy	0.472	Xtriage
Bulk solvent k_{sol}(e/Å^3), B_{sol}(Å^2)	(Not available) , (Not available)	EDS
L-test for twinning[2]	$< \|L\| >= 0.50$, $< L^2 > = 0.33$	Xtriage
Estimated twinning fraction	No twinning to report.	Xtriage
F_o,F_c correlation	0.91	EDS
Total number of atoms	6027	wwPDB-VP
Average B, all atoms (Å^2)	75.0	wwPDB-VP

Xtriage's analysis on translational NCS is as follows: *The largest off-origin peak in the Patterson function is 6.10% of the height of the origin peak. No significant pseudotranslation is detected.*

[1] Intensities estimated from amplitudes.

[2] Theoretical values of $< |L| >$, $< L^2 >$ for acentric reflections are 0.5, 0.333 respectively for untwinned datasets, and 0.375, 0.2 for perfectly twinned datasets.

CONFIDENTIAL VALIDATION REPORT

5 Model quality (i)

5.1 Standard geometry (i)

The Z score for a bond length (or angle) is the number of standard deviations the observed value is removed from the expected value. A bond length (or angle) with $|Z| > 5$ is considered an outlier worth inspection. RMSZ is the root-mean-square of all Z scores of the bond lengths (or angles).

Mol	Chain	Bond lengths		Bond angles	
		RMSZ	#$|Z|$ >5	RMSZ	#$|Z|$ >5
1	A	0.30	0/3020	0.58	2/4088 (0.0%)
1	B	0.37	0/3079	0.64	1/4166 (0.0%)
All	All	0.34	0/6099	0.61	3/8254 (0.0%)

There are no bond length outliers.

All (3) bond angle outliers are listed below:

Mol	Chain	Res	Type	Atoms	Z	Observed(^{o})	Ideal(^{o})
1	A	86	LEU	CB-CG-CD2	-5.22	102.13	111.00
1	A	336	GLU	N-CA-C	-5.12	97.19	111.00
1	B	82	GLU	CA-CB-CG	5.09	124.61	113.40

There are no chirality outliers.

There are no planarity outliers.

5.2 Too-close contacts (i)

In the following table, the Non-H and H(model) columns list the number of non-hydrogen atoms and hydrogen atoms in the chain respectively. The H(added) column lists the number of hydrogen atoms added and optimized by MolProbity. The Clashes column lists the number of clashes within the asymmetric unit, whereas Symm-Clashes lists symmetry-related clashes.

Mol	Chain	Non-H	H(model)	H(added)	Clashes	Symm-Clashes
1	A	2983	0	2988	58	0
1	B	3044	0	3051	83	1
All	All	6027	0	6039	137	1

The all-atom clashscore is defined as the number of clashes found per 1000 atoms (including hydrogen atoms). The all-atom clashscore for this structure is 11.

All (137) close contacts within the same asymmetric unit are listed below, sorted by their clash magnitude.

CONFIDENTIAL VALIDATION REPORT

Atom-1	Atom-2	Interatomic distance (Å)	Clash overlap (Å)
1:B:21:LEU:HD11	1:B:28:LEU:HD23	1.57	0.84
1:B:47:ALA:HA	1:B:385:GLY:HA3	1.64	0.79
1:A:378:VAL:HG21	1:A:418:GLU:HB3	1.64	0.79
1:B:214:ILE:HG21	1:B:229:PRO:HG2	1.64	0.78
1:A:40:GLN:HE22	1:A:444:ILE:H	1.32	0.77
1:A:44:SER:HA	1:A:363:VAL:HG22	1.66	0.76
1:B:367:ASN:HD22	1:B:383:ARG:HD3	1.50	0.74
1:B:98:LEU:HD11	1:B:300:ALA:HB3	1.69	0.73
1:B:308:GLU:OE1	1:B:391:GLN:HG2	1.92	0.70
1:B:398:GLY:HA2	1:B:401:GLN:HG3	1.74	0.70
1:B:64:LEU:HD22	1:B:84:VAL:HG12	1.74	0.69
1:B:189:ALA:HB1	1:B:367:ASN:HD21	1.58	0.68
1:B:14:LEU:HD21	1:B:61:SER:HB2	1.74	0.67
1:B:344:LEU:HB3	1:B:348:MET:HB3	1.77	0.66
1:A:343:ARG:HG3	1:A:369:VAL:HG21	1.78	0.66
1:B:123:LEU:HD21	1:B:182:LEU:HB2	1.76	0.65
1:B:261:LYS:HA	1:B:268:THR:HA	1.79	0.65
1:B:10:GLN:HE21	1:B:14:LEU:HD13	1.62	0.64
1:A:47:ALA:HA	1:A:385:GLY:HA3	1.80	0.64
1:A:46:VAL:HG23	1:A:49:CYS:HB3	1.80	0.64
1:B:176:LEU:HA	1:B:179:ARG:HH11	1.62	0.64
1:B:98:LEU:HD13	1:B:297:LEU:HD13	1.78	0.63
1:B:366:LYS:NZ	1:B:377:PHE:O	2.24	0.63
1:B:21:LEU:HD12	1:B:25:ASP:HB3	1.81	0.62
1:B:88:GLU:O	1:B:92:ILE:HG12	1.98	0.62
1:A:88:GLU:HA	1:A:290:MET:CE	2.30	0.62
1:B:408:ARG:HD2	1:B:431:PHE:HE1	1.65	0.62
1:A:397:GLN:HG2	1:A:401:GLN:HE21	1.65	0.61
1:A:50:CYS:HB2	1:A:391:GLN:HE22	1.65	0.61
1:B:182:LEU:HD12	1:B:209:ILE:HB	1.83	0.60
1:A:121:ALA:HB1	1:A:272:VAL:HG12	1.83	0.60
1:A:64:LEU:HD11	1:A:84:VAL:HG12	1.84	0.58
1:B:352:ILE:HG23	1:B:421:PHE:HB2	1.84	0.58
1:A:26:ALA:O	1:A:30:ARG:HD2	2.04	0.57
1:A:88:GLU:HA	1:A:290:MET:HE2	1.86	0.57
1:B:81:CYS:O	1:B:84:VAL:HG22	2.04	0.57
1:B:416:LEU:HB2	1:B:422:THR:HG21	1.87	0.57
1:B:98:LEU:HD21	1:B:222:ALA:HB1	1.87	0.57
1:A:65:VAL:HG11	1:B:57:LEU:HD21	1.87	0.57
1:A:343:ARG:NH1	1:A:370:PRO:O	2.38	0.57
1:B:46:VAL:HG13	1:B:363:VAL:HG12	1.87	0.56
1:A:65:VAL:HB	1:A:288:ASN:HD21	1.71	0.56

Continued on next page...

CONFIDENTIAL VALIDATION REPORT

Continued from previous page...

Atom-1	Atom-2	Interatomic distance (Å)	Clash overlap (Å)
1:B:347:ALA:HA	1:B:374:ARG:NH1	2.20	0.56
1:A:196:PHE:CG	1:A:229:PRO:HB3	2.41	0.56
1:B:53:LYS:HE3	1:B:299:TYR:HE2	1.70	0.56
1:A:225:ARG:HH21	1:A:310:GLN:HB2	1.71	0.56
1:A:128:THR:HB	1:A:183:ILE:HG23	1.88	0.55
1:B:110:SER:HB3	1:B:113:SER:H	1.71	0.54
1:B:123:LEU:HD22	1:B:181:ARG:HG2	1.90	0.54
1:A:21:LEU:HD11	1:A:28:LEU:HD23	1.90	0.54
1:A:40:GLN:NE2	1:A:444:ILE:H	2.04	0.54
1:A:190:TYR:CE2	1:A:192:ARG:HB2	2.43	0.54
1:B:10:GLN:HE21	1:B:14:LEU:CD1	2.21	0.53
1:B:53:LYS:HG3	1:B:299:TYR:CE2	2.44	0.53
1:A:436:GLU:O	1:A:440:VAL:HG23	2.10	0.52
1:A:119:LEU:HD21	1:A:184:ILE:HD11	1.90	0.52
1:A:168:TYR:HB2	1:A:198:ARG:CZ	2.40	0.52
1:B:196:PHE:CE2	1:B:229:PRO:HG3	2.45	0.51
1:B:165:LEU:HD21	1:B:192:ARG:NE	2.24	0.51
1:A:251:LEU:HD11	1:A:297:LEU:HD21	1.92	0.51
1:A:287:VAL:HG12	1:A:289:MET:H	1.74	0.50
1:B:375:SER:HB2	1:B:378:VAL:HG12	1.92	0.50
1:B:408:ARG:HD2	1:B:431:PHE:CE1	2.45	0.50
1:A:313:ARG:NH1	1:A:336:GLU:HA	2.27	0.50
1:B:159:GLY:O	1:B:166:ILE:HA	2.12	0.50
1:A:397:GLN:HG2	1:A:401:GLN:NE2	2.27	0.49
1:B:38:ARG:HA	1:B:41:ARG:HE	1.78	0.49
1:A:50:CYS:HB2	1:A:391:GLN:NE2	2.28	0.48
1:A:35:GLU:OE2	1:B:83:ASN:HB2	2.13	0.48
1:B:300:ALA:HA	1:B:305:PHE:CG	2.49	0.47
1:A:31:ILE:HG21	1:B:87:VAL:HG23	1.95	0.47
1:A:64:LEU:HD21	1:A:87:VAL:HG11	1.97	0.47
1:A:447:TYR:C	1:B:8:LEU:HD11	2.35	0.47
1:B:168:TYR:CD1	1:B:198:ARG:HD3	2.49	0.47
1:B:82:GLU:O	1:B:86:LEU:HD22	2.13	0.47
1:B:162:LYS:HB2	1:B:165:LEU:HB2	1.97	0.47
1:A:214:ILE:HG21	1:A:229:PRO:HG3	1.96	0.47
1:A:265:ARG:HH12	1:A:272:VAL:HG11	1.80	0.47
1:B:235:VAL:HG22	1:B:273:LEU:HD11	1.96	0.47
1:B:45:LEU:HB2	1:B:364:VAL:HG12	1.97	0.46
1:B:223:THR:HG21	1:B:309:MET:HG3	1.96	0.46
1:B:374:ARG:NH2	1:B:380:SER:OG	2.47	0.46
1:A:64:LEU:O	1:A:67:VAL:HG22	2.16	0.46

Continued on next page...

CONFIDENTIAL VALIDATION REPORT

Continued from previous page...

Atom-1	Atom-2	Interatomic distance (Å)	Clash overlap (Å)
1:B:99:PHE:O	1:B:230:ILE:HG13	2.15	0.46
1:B:189:ALA:HB1	1:B:367:ASN:ND2	2.29	0.46
1:B:38:ARG:HA	1:B:41:ARG:NE	2.31	0.45
1:B:167:ASP:O	1:B:171:VAL:HG23	2.17	0.45
1:A:28:LEU:HG	1:A:32:LEU:HD23	1.99	0.45
1:B:56:THR:HG21	1:B:246:GLY:HA2	1.98	0.45
1:A:28:LEU:O	1:A:32:LEU:HD23	2.17	0.45
1:A:343:ARG:CG	1:A:369:VAL:HG21	2.47	0.45
1:B:251:LEU:HD22	1:B:253:LEU:HG	1.99	0.44
1:A:199:PHE:HB3	1:A:210:LEU:HD11	1.99	0.44
1:A:176:LEU:HD21	1:A:205:GLU:HG3	1.99	0.44
1:A:336:GLU:O	1:A:337:ASN:HB2	2.18	0.44
1:B:347:ALA:HA	1:B:374:ARG:HH12	1.82	0.44
1:B:88:GLU:OE2	1:B:107:GLN:NE2	2.38	0.44
1:A:293:LYS:O	1:A:297:LEU:HG	2.18	0.44
1:B:430:GLN:H	1:B:430:GLN:HG3	1.64	0.44
1:B:10:GLN:HE22	1:B:13:LEU:HD23	1.82	0.43
1:B:182:LEU:CD1	1:B:209:ILE:HB	2.48	0.43
1:A:111:ALA:HB2	1:A:239:CYS:HB2	2.01	0.43
1:A:342:ILE:HG12	1:A:382:LEU:HG	2.00	0.43
1:A:416:LEU:O	1:A:420:GLU:HB2	2.19	0.43
1:B:342:ILE:HG12	1:B:382:LEU:HB3	2.01	0.42
1:A:32:LEU:HD13	1:A:32:LEU:HA	1.91	0.42
1:B:265:ARG:HG3	1:B:267:ALA:H	1.84	0.42
1:A:128:THR:HG22	1:A:158:TYR:HD1	1.83	0.42
1:A:355:THR:O	1:A:358:GLU:HG2	2.20	0.42
1:A:240:THR:O	1:A:244:LEU:HB2	2.19	0.42
1:A:313:ARG:HD2	1:A:313:ARG:HA	1.79	0.42
1:B:131:GLY:O	1:B:132:MET:C	2.59	0.42
1:B:168:TYR:CG	1:B:198:ARG:HD3	2.55	0.42
1:B:313:ARG:HD2	1:B:313:ARG:HA	1.82	0.41
1:B:45:LEU:HB3	1:B:384:ILE:HG23	2.02	0.41
1:A:53:LYS:HA	1:A:53:LYS:HD3	1.81	0.41
1:B:323:PHE:HZ	1:B:406:LEU:HD23	1.85	0.41
1:B:344:LEU:HD23	1:B:344:LEU:HA	1.86	0.41
1:B:46:VAL:HG23	1:B:49:CYS:HB3	2.02	0.41
1:B:53:LYS:HG3	1:B:299:TYR:CD2	2.55	0.41
1:B:132:MET:HA	1:B:157:GLY:HA3	2.03	0.41
1:B:429:LYS:HB3	1:B:429:LYS:HE3	1.73	0.41
1:A:372:GLU:OE2	1:A:374:ARG:HB2	2.20	0.41
1:A:81:CYS:O	1:A:84:VAL:HG22	2.21	0.41

Continued on next page...

CONFIDENTIAL VALIDATION REPORT

Continued from previous page...

Atom-1	Atom-2	Interatomic distance (Å)	Clash overlap (Å)
1:B:83:ASN:HA	1:B:86:LEU:HD23	2.03	0.41
1:B:364:VAL:HG21	1:B:382:LEU:HD21	2.02	0.41
1:A:300:ALA:HA	1:A:305:PHE:CG	2.55	0.41
1:A:310:GLN:O	1:A:314:ASP:N	2.46	0.41
1:B:334:ARG:HD3	1:B:334:ARG:N	2.36	0.40
1:B:289:MET:O	1:B:293:LYS:HG3	2.21	0.40
1:B:21:LEU:O	1:B:21:LEU:HG	2.21	0.40
1:B:228:SER:HA	1:B:229:PRO:HD3	1.83	0.40
1:B:10:GLN:O	1:B:14:LEU:HD13	2.21	0.40
1:B:187:ALA:HB3	1:B:190:TYR:HB2	2.02	0.40
1:B:316:ALA:HA	1:B:340:ILE:HD11	2.04	0.40
1:B:398:GLY:O	1:B:402:ILE:HG13	2.20	0.40
1:A:375:SER:HB3	1:A:378:VAL:HG12	2.04	0.40

All (1) symmetry-related close contacts are listed below. The label for Atom-2 includes the symmetry operator and encoded unit-cell translations to be applied.

Atom-1	Atom-2	Interatomic distance (Å)	Clash overlap (Å)
1:B:260:GLU:OE2	1:B:261:LYS:CD[2_655]	1.75	0.45

5.3 Torsion angles (i)

5.3.1 Protein backbone (i)

In the following table, the Percentiles column shows the percent Ramachandran outliers of the chain as a percentile score with respect to all X-ray entries followed by that with respect to entries of similar resolution.

The Analysed column shows the number of residues for which the backbone conformation was analysed, and the total number of residues.

Mol	Chain	Analysed	Favoured	Allowed	Outliers	Percentiles	
1	A	388/460 (84%)	377 (97%)	11 (3%)	0	100	100
1	B	398/460 (86%)	383 (96%)	14 (4%)	1 (0%)	41	70
All	All	786/920 (85%)	760 (97%)	25 (3%)	1 (0%)	51	80

All (1) Ramachandran outliers are listed below:

CONFIDENTIAL VALIDATION REPORT

Mol	Chain	Res	Type
1	B	425	PRO

5.3.2 Protein sidechains (i)

In the following table, the Percentiles column shows the percent sidechain outliers of the chain as a percentile score with respect to all X-ray entries followed by that with respect to entries of similar resolution.

The Analysed column shows the number of residues for which the sidechain conformation was analysed, and the total number of residues.

Mol	Chain	Analysed	Rotameric	Outliers	Percentiles	
1	A	308/358 (86%)	306 (99%)	2 (1%)	86	95
1	B	312/358 (87%)	298 (96%)	14 (4%)	27	59
All	All	620/716 (87%)	604 (97%)	16 (3%)	46	78

All (16) residues with a non-rotameric sidechain are listed below:

Mol	Chain	Res	Type
1	A	339	THR
1	A	341	LEU
1	B	129	LEU
1	B	132	MET
1	B	134	LEU
1	B	245	VAL
1	B	259	ASN
1	B	334	ARG
1	B	337	ASN
1	B	339	THR
1	B	341	LEU
1	B	416	LEU
1	B	423	LEU
1	B	427	LEU
1	B	429	LYS
1	B	430	GLN

Sometimes sidechains can be flipped to improve hydrogen bonding and reduce clashes. All (7) such sidechains are listed below:

Mol	Chain	Res	Type
1	A	40	GLN
1	A	401	GLN

Continued on next page...

CONFIDENTIAL VALIDATION REPORT

Continued from previous page...

Mol	Chain	Res	Type
1	B	10	GLN
1	B	83	ASN
1	B	337	ASN
1	B	397	GLN
1	B	401	GLN

5.3.3 RNA ⓘ

There are no RNA molecules in this entry.

5.4 Non-standard residues in protein, DNA, RNA chains ⓘ

There are no non-standard protein/DNA/RNA residues in this entry.

5.5 Carbohydrates ⓘ

There are no monosaccharides in this entry.

5.6 Ligand geometry ⓘ

There are no ligands in this entry.

5.7 Other polymers ⓘ

There are no such residues in this entry.

5.8 Polymer linkage issues ⓘ

There are no chain breaks in this entry.

CONFIDENTIAL VALIDATION REPORT

6 Fit of model and data (i)

6.1 Protein, DNA and RNA chains (i)

Unable to reproduce the depositors R factor - this section is therefore empty.

6.2 Non-standard residues in protein, DNA, RNA chains (i)

Unable to reproduce the depositors R factor - this section is therefore empty.

6.3 Carbohydrates (i)

Unable to reproduce the depositors R factor - this section is therefore empty.

6.4 Ligands (i)

Unable to reproduce the depositors R factor - this section is therefore empty.

6.5 Other polymers (i)

Unable to reproduce the depositors R factor - this section is therefore empty.

CONFIDENTIAL VALIDATION REPORT
